# Cyclopentadienone Iron Tricarbonyl Complexes-Catalyzed Hydrogen Transfer in Water

**DOI:** 10.3390/molecules25020421

**Published:** 2020-01-20

**Authors:** Daouda Ndiaye, Sébastien Coufourier, Mbaye Diagne Mbaye, Sylvain Gaillard, Jean-Luc Renaud

**Affiliations:** 1 Normandie Univ., LCMT, ENSICAEN, UNICAEN, CNRS, 6 boulevard du Maréchal Juin, 14050 Caen, France; 2 Université Assane Seck de Ziguinchor, BP 523, Ziguinchor, Senegal

**Keywords:** iron complexes, hydrogen transfer, reductive amination, alcohols, amines

## Abstract

The development of efficient and low-cost catalytic systems is important for the replacement of robust noble metal complexes. The synthesis and application of a stable, phosphine-free, water-soluble cyclopentadienone iron tricarbonyl complex in the reduction of polarized double bonds in pure water is reported. In the presence of cationic bifunctional iron complexes, a variety of alcohols and amines were prepared in good yields under mild reaction conditions.

## 1. Introduction

The reduction of polarized C=X bonds is an important process, both in industry and in academia, for the synthesis of fine chemicals, perfumes, agrochemicals, and pharmaceuticals [1,2,3,4,5,6,7,8]. To avoid the use of stoichiometric amounts of borohydrides or aluminium hydrides, metal-catalyzed pathways to amines and alcohols have been introduced [9,10,11,12,13,14]. These procedures consist of hydrogenation, hydrosilylation, and transfer hydrogenation (with *iso*-propanol or formic acid) and involve mainly platinum complexes [9,10], but recent contributions highlighted the rise of Earth-abundant metals for such reductions [11,12,13,14]. Hydrogenation is the most atom economical approach, but requires hydrogen gas handling and consequently implies some safety issues. Hydrogen transfer (TH) is an alternative pathway and a more practical tool. Alcohols and formic acid (or formates) are among the most advantageous hydride donors.

**Figure 1:**
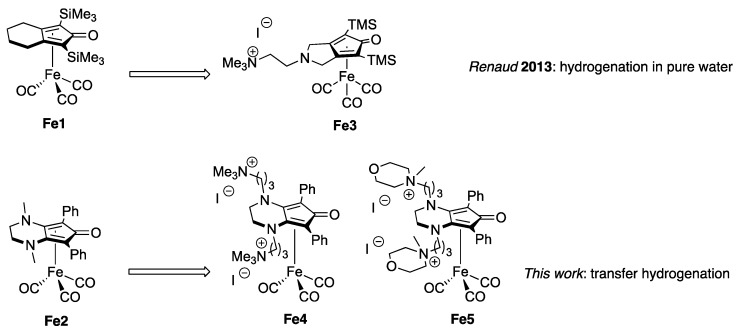
Previous water-soluble cyclopentadienone iron complex and new complexes for application reduction.

Water is a non-toxic, non-flammable, non-explosive and also an economically relevant solvent [15,16]. Water-soluble organometallic complexes have attracted some interest because of the environmentally acceptable process, the simple product separation and, in some reactions, the possibility to control the selectivity by adjusting the pH [15,16]. Despite these advantages, the use of water in catalysis, and more specifically, in reduction, still constitutes a challenge and is underexplored compared to organic solvent [17,18]. Hydrogenation of ketones and imines [19], and reductive amination [20] in water have been reported with few iron complexes. As an example; our group has disclosed the first water-soluble and well-defined cyclopentadienone iron complex able to catalyze the reduction of aldehydes, ketones, and 2-substituted dihydroisoquinolines in pure water at 85–100 °C under hydrogen pressure (Figure 1, for the first synthesis of a water-soluble cyclopentadienone iron complex, see [19]). Little is known on the transfer hydrogenation with Earth-abundant complexes, while formates are used by enzymes for enantioselective reduction. To the best of our knowledge, excepted the hydrogen transfer reduction of heterocyclic compounds with formic acid catalyzed by a cobalt-phosphines complex [20], no reduction of polarized C=X bonds (aldehydes, ketones, and imines) with formic acid derivatives has been yet reported. Toward this objective, we thought of developing new water-soluble non-phosphine ligand iron complexes for the reduction of polarized bonds in the presence of formates or formic acid in pure water.

In 2015, we introduced in catalysis the tricarbonyl iron complex **Fe2** bearing a diaminocyclopentadienone ligand [21]. Compared to other cyclopentadienone iron carbonyl complexes, this phosphine-free iron complex has, to the best of our knowledge, the highest catalytic activities to date in reductive amination [21], in chemoselective reduction of α,β-unsaturated ketones [22], in the hydrogenation of carbon dioxide [23], in alkylation of ketones [24,25,26,27], amines [25,28], oxindoles [29], indoles [25,30] and alcohols [31,32]. In our ongoing interest in reduction and alkylation, we thought that a water-soluble analog of **Fe2** would be more active than our previous water-soluble cyclopentadienone iron complex **Fe3** (Figure 1). In this work, we report on the synthesis and application of two water-soluble cyclopentadienone iron complexes in the reduction of aldehydes and in reductive amination in pure water.

## 2. Results and Discussion

### 2.1. Synthesis of Complexes

**Scheme 1:**
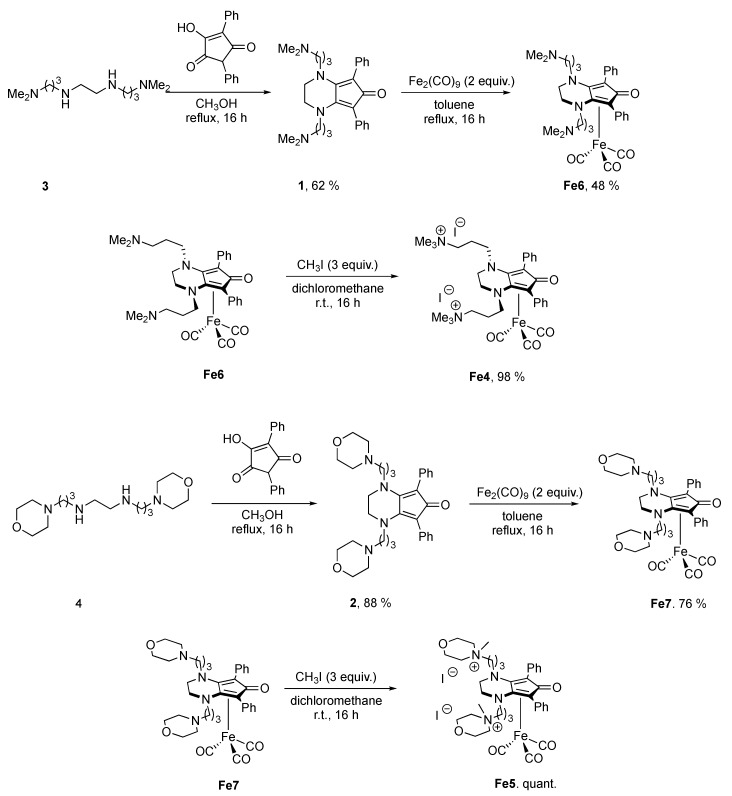
Synthesis of the iron complexes **Fe4**, **Fe5**, **Fe6,** and **Fe7**.

To develop water-soluble iron complexes, we selected a diaminocyclopentadienone ligand bearing ammonium functionalities [33]. The tetraamines **2** and **4** were prepared from diethyloxalate and *N*,*N*-dimethylpropylenediamine and *N*-aminopropylenemorpholine via an amidation followed by a reduction in good overall yield (93 and 95%, respectively). The corresponding aminocyclopentadienone ligands **1** and **2** were then prepared by reacting the amines **2** and **4** with the cyclopentatrienone in refluxing methanol for 16 h and were isolated in moderate yield (62% and 88%, respectively, Scheme 1). The complexes **Fe6** and **Fe7** were synthesized in 48% and 76% yield by simple heating of the corresponding amino ligand with [Fe_2_(CO)_9_] in refluxing toluene (Scheme 1). Finally, the water-soluble bifunctional iron complexes **Fe4** and **Fe5** bearing ionic frameworks were obtained in almost quantitative yields after a subsequent alkylation of the pendant amines with iodomethane (Scheme 1) [33]. These complexes were fully characterized by ^1^H-, ^13^C-NMR, and IR spectroscopies (see Supplementary Materials). These analyses showed that complexes **Fe2** and **Fe4-Fe7** have similar features. The back donation from the metal center to the CO ligands is more significant than in the Knölker’s complex **Fe1** [34,35]. Thus, the CO stretching frequencies were at 2032, 1961, and 1919 cm^−1^ and at 2015 and 1967 cm^−1^ in the neutral complexes **Fe6** and **Fe7**, respectively, at 2038 and 1955 cm^−1^ and at 2034 and 1957 cm^−1^ in the ionic complexes **Fe4** and **Fe5**, respectively. These frequencies are comparable to those of the analog **Fe2** (2027, 1962 and 1947 cm^−1^) and lower than those of the Knölker’s complex **Fe1** (2061, 2053, and 1987 cm^−1^) or its water-soluble analog **Fe3** (2066, 2016, and 1996 cm^−1^) [19].

### 2.2. Iron-Catalyzed Reduction of Carbonyl Compounds

With these complexes in hands, we evaluated their catalytic activities in the reduction of 4-methoxybenzaldehyde as a benchmark reaction. Various methods of activation can be used with the cyclopentadienone iron carbonyl complexes [34,35,36,37,38,39,40,41,42,43,44]. We applied in this work the activation with Me_3_NO as oxidant [34,35,36,37,38,39,40,41,42]. Our first attempt with formic acid, in the presence of 2 mol % of **Fe4** and 2.5 mol % of Me_3_NO at 100 °C for 24 h in 2 mL of pure water (concentration of 0.5 M), was unsuccessful as no reduction was noticed (entry 1, Table 1). In sharp contrast, in the same reaction conditions, complete conversions were obtained with different formate salts (entries 2–5, Table 1). Without a hydride donor or iron complex, no reduction occurred (entries 6–7, Table 1). Decreasing the reaction time (entries 8 and 11, Table 1), the catalyst loading (entry 13) and the amount of formate (entry 14) led to a drop in the conversion. No variation of the conversion was noticed by lowering the temperature to 80 °C (entries 3 and 9, Table 1), while, at 60 °C, the conversion was only 75% (entry 12). To our surprise, the first generation water-soluble cyclopentadienone iron complex **Fe3** did not catalyze the reduction of 4-methoxybenzaldehyde in these conditions, while **Fe5** appeared as active as **Fe4** (entries 10–15, Table 1). Finally, the best conditions for the reduction of 4-methoxybenzaldehyde into the corresponding alcohol **4a** were: 1 mmol of aldehyde, in the presence of five equivalents of sodium formate in 2 mL of water, 2 mol % of **Fe4** or **Fe5** and 2.5 mol % of Me_3_NO at 80 °C for 24 h.

Having established the optimized conditions, we delineated the scope of the carbonyl derivatives (Table 2). Both electron-donating (methoxy, methyl, acetal substituents) and electron-withdrawing (nitro, nitrile, and ester substituents) groups were tolerated in this reduction. The corresponding alcohols **4a**–**m** were isolated in excellent yields in all examples (91–99%, Table 2). No reduction of halogen-carbon bonds in the substituted phenyl group (compounds **4i**–**j**) was observed. Other reducible functions, such as ester or nitrile, were preserved in these conditions. Heteroaromatic derivatives, such as pyridine or thiophene carboxaldehyde, furfural, did not impede the catalytic activity and provided the alcohols **4n**–**r** in 75–98%yield (Table 2). Finally, to extend the scope, aliphatic aldehydes were also engaged in this reduction and the corresponding alcohols **4s**–**v** were isolated in 92–99% yield (Table 2). It is worth to mention that (i) ethanol was used as a co-solvent with some substrates to facilitate the solubility and consequently enhanced the reactivity; and (ii) no reaction occurred in a mixture of water and ethanol without sodium formate.

### 2.3. Iron-Catalyzed Reductive Amination

Having established a simple protocol for the reduction of aldehydes in water, we thought to extend this work to the synthesis of amines. Amines are usually prepared via the reduction of C=N bonds either in catalytic conditions under hydrogen pressure or in stoichiometric conditions in the presence of aluminum/boron hydride [45]. However, imines are not always easily prepared and cannot be stable. Reductive amination of aldehydes constitutes a direct route to amines, without requiring any purification of the imine intermediate. Many efforts have been devoted to the development of reductive amination [46,47,48]. For example, in iron chemistry, Bhanage described that a combination of iron sulfate and ethylenediaminetetraacetic acid (EDTA) catalyzed a reductive amination under hydrogen pressure (400 psi) in water at elevated temperatures (150 °C) [20]. Beller reported a reductive amination with anilines catalyzed by Fe_2_(CO)_9_ under high hydrogen pressure and elevate temperature [49]. We have reported that cyclopentadienone iron tricarbonyl complexes [21,34,35] or cyclopentadienyl iron(II) tricarbonyl complex [50] were able to catalyzed the reductive alkylation of various amines and carbonyl derivatives under 5 bar of hydrogen, at 40–70 °C and even room temperature. To avoid the use of a large amount of hydride (and the concomitant formation of wastes) or the handling of gas, hydrogen transfer with formate derivatives appears as a simple and versatile procedure. The reductive alkylation of *N*-methylbenzylamine with citronellal was chosen as a model reaction for the optimization of the reaction conditions. Three formate salts were tested, and the cation appeared to be crucial for the catalytic activity (entries 1–4, Table 3). Indeed, the ammonium favored both the condensation and the reduction (via the formation of an iminium intermediate). **Fe4** and **Fe5** provided the alkylated amines with the same conversion and selectivities (entries 1–2, Table 3). Both water-soluble complexes **Fe4** and **Fe5** could then be used in this reaction (as it was also mentioned in the reduction of aldehydes), but for the rest of the study, we will use **Fe5** as pre-catalyst as the overall conversion in reductive amination is somewhat higher. Without a hydride donor, no reduction occurred (entries 5, Table 3), and only the imine was obtained. Decreasing the temperature was detrimental to the catalytic activity as a drop of the conversion in amine was noticed (entries 6–8, Table 3). Finally, an increase of the amount of ammonium formate to 6.5 equivalent furnished the alkylated amine in 70% isolated yield (entry 9, Table 3).

With the optimized conditions in hands, we evaluated some aliphatic and benzylic amines with aromatic and aliphatic aldehydes (Table 4). Whatever the benzylic amine used with citronellal, the isolated yield was good (**5a**–**b**, 61–70%), while the alkylated amine **5c** was obtained in a low yield (21%) from the 2-phenylethylamine (Table 4). Amines **5d**–**k** were prepared in 11–64% yield from various benzaldehydes. First, as observed previously, no reduction of halogen-carbon bonds in the substituted phenyl group was observed, and the corresponding alkylated amines **5f**–**h** were obtained in around 50% yield. The reductive alkylation of *N*-methylbenzylamine with thiophene carboxaldehyde furnished the corresponding amine in a 64% yield. The reductive amination with electron-rich benzaldehyde and *N*-methylbenzylamine led to the alkylated amine **5j** in very modest yields (13%, Table 4).

### 2.4. Recycling of the Water-Soluble Iron Complex

One of the main goals, when reactions are carried out in the water, is the study of the recyclability of the complex used. Due to the high solubility of the iron complexes, **Fe4**–**5** in water, separation and recycling should be possible to perform. **Fe5**-catalyzed, the reductive alkylation of *N*-methylbenzylamine with citronellal, was chosen as a model reaction for this study. At the end of the first run, ethyl acetate was added under an argon atmosphere to extract the organic compounds, and the aqueous phase was re-engaged directly in another run after the addition of ammonium formate, amine, and aldehyde (Table 5). As showed in Table 5, catalytic activity was maintained after five runs without any decrease in the conversion.

## 3. Materials and Methods

All air- and moisture-sensitive manipulations were carried out using standard vacuum line Schlenk tubes techniques. All solvent and substrates were degassed prior to use by bubbling argon gas directly in the reaction medium. Other solvents and chemicals were purchased from different suppliers and used as received. Deuterated solvents for NMR spectroscopy were purchased from Sigma Aldrich (Saint-Quentin Fallavier, France) and used as received. NMR spectra were recorded on a 500 MHz Bruker spectrometer. Proton (^1^H) NMR information is given in the following format: multiplicity (s, singlet; d, doublet; t, triplet; q, quartet; m, multiplet), coupling constant(s) (*J*) in Hertz (Hz), number of protons, type. The prefix *app* is occasionally applied when the true signal multiplicity was unresolved, and *br* indicates the signal in question broadened. Carbon (^13^C) NMR spectra are reported in ppm (δ) relative to CDCl_3_ unless noted otherwise. Infrared spectra were recorded over a PerkinElmer Spectrum 100 FT-IR Spectrometer using neat conditions. HRMS analyses were performed by using the Laboratoire de Chimie Moléculaire et Thioorganique analytical Facilities.

### 3.1. General Procedure for the Reduction of Aldehydes

In a dried flamed Schlenk tube under argon, the corresponding aldehyde (1 equiv.) and sodium formate (5 equiv.) were mixed in water (0.5 M solution). The iron complex **Fe4** (2 mol %) and Me_3_NO (2.5 mol %) were then added. The mixture was stirred and heated at 80 °C for 24 h. After cooling down to room temperature, the resulting solution was quenched with a saturated aqueous solution of sodium bicarbonate and extracted three times with ethyl acetate. The organic phase was dried over MgSO_4_, filtrated, and concentrated under vacuum to afford the crude product. Purification by flash chromatography on silica gel furnished the alcohol.

### 3.2. General Procedure for the Reductive Amination of Aldehydes

In a dried flamed Schlenk tube under argon, the aldehyde (1 equiv.), the amine (2 equiv.) and ammonium formate (6.5 equiv.) were mixed in water (0.5 M solution). The iron complex **Fe5** (2 mol %) and Me_3_NO (2.5 mol %) were then added. The mixture was stirred and heated at 90 °C for 24–48 h. After cooling down to room temperature, the resulting solution was quenched with a saturated aqueous solution of sodium bicarbonate and extracted three times with ethyl acetate. The organic phase was dried over MgSO_4_, filtrated, and concentrated under vacuum to afford the crude product. Purification by flash chromatography on silica gel furnished the amine.

## 4. Conclusions

In conclusion, we have described the application of water-soluble cyclopentadienone iron tricarbonyl complexes in the reduction of aldehydes and in reductive amination under hydride transfer conditions in pure water. Recyclability of iron complex **Fe5** was also demonstrated in a model reductive amination. This system tolerated a variety of functional groups such as halides, ethers, heteroaromatic derivatives without impeding the chemical yields. These water-soluble iron complexes allow an efficient, green, and practical procedure for the synthesis of amines and alcohols.

## Figures and Tables

**Table 1:** Optimization of the reaction conditions for the aldehyde reduction ^a^.

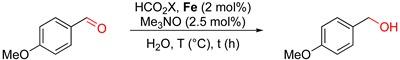					
Entry	HCO_2_X	Fe	Temperature (°C)	Time (h)	Conv. (%) ^b^
1	HCO_2_H	**Fe4**	100	24	0
2	HCO_2_H/Et_3_N (1/1)	**Fe4**	100	24	100
3	HCO_2_Na	**Fe4**	100	24	100
4	HCO_2_K	**Fe4**	100	24	100
5	HCO_2_Cs	**Fe4**	100	24	100
6	-	**Fe4**	100	24	0
7	HCO_2_Na	-	100	24	0
8	HCO_2_Na	**Fe4**	100	16	83
9	HCO_2_Na	**Fe4**	80	24	100 (99%) ^c^
10	HCO_2_Na	**Fe3**	80	24	0
11	HCO_2_Na	**Fe4**	80	16	81
12	HCO_2_Na	**Fe4**	60	24	75
13 ^d^	HCO_2_Na	**Fe4**	80	24	53
14 ^e^	HCO_2_Na	**Fe4**	80	24	86
15	HCO_2_Na	**Fe5**	80	24	100 (98%) ^c^

^a^ General conditions: HCO_2_X (5 mmol, 5 equiv.), 4-methoxybenzaldehyde (1 mmol), pre-catalyst (2 mol %), Me_3_NO (2.5 mol %), water (2 mL). ^b^ Conversion was determined by ^1^H-NMR spectroscopy analysis. ^c^ Isolated yield in the bracket. ^d^ **Fe** 0(1 mol %), Me_3_NO (1.25 mol %) were used. ^e^ HCO_2_Na (3 mmol, three equiv.) were used.

**Table 2:** Iron-catalyzed reduction of aldehydes with sodium formate ^a^.

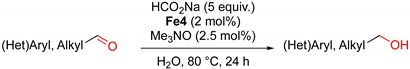
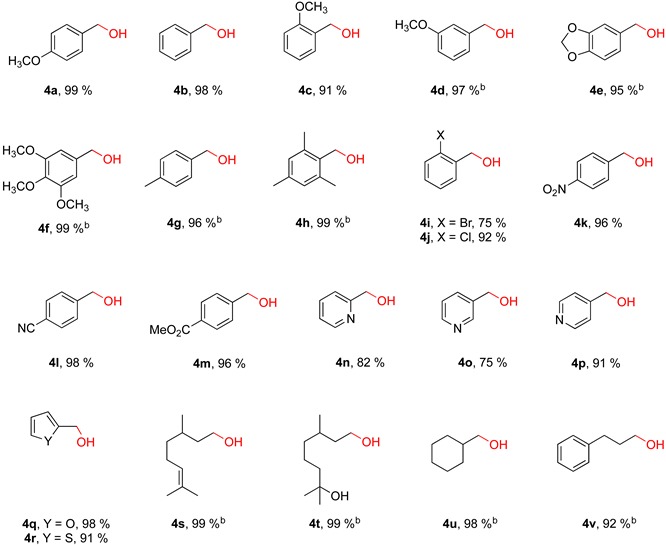

^a^ General conditions: aldehyde (1 mmol), HCO_2_Na (5 mmol, 5 equiv.), pre-catalyst **Fe4** (2 mol %), Me_3_NO (2.5 mol %), water (2 mL). ^b^ H_2_O/EtOH 1/1.

**Table 3:** Optimization of the reaction conditions for the reductive amination ^a^.

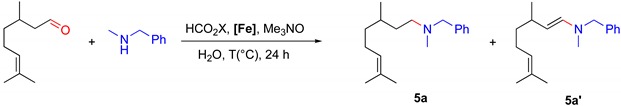					
Entry	HCO_2_X (equiv.)	[Fe]	T (°C)	Conv. (%) ^b^	Selectivity (5a)/(5a’) ^b^
1	HCO_2_NH_4_ (5)	**Fe4**	90	93	77/23
2	HCO_2_NH_4_ (5)	**Fe5**	90	95	77/23
3	HCO_2_K (5)	**Fe5**	90	94	60/40
4	HCO_2_Cs (5)	**Fe5**	90	93	40/60
5	-	**Fe5**	90	100	0/100
6	HCO_2_NH_4_ (5)	**Fe5**	85	91	67/33
7	HCO_2_NH_4_ (5)	**Fe5**	80	83	69/31
8	HCO_2_NH_4_ (5)	**Fe5**	40	80	52.5/47.5
9	HCO_2_NH_4_ (6.5)	**Fe5**	90	96	91/9 (70) ^c^

^a^ General conditions: HCO_2_X (5 mmol, 5 equiv.), citronellal (1 mmol), *N*-methylbenzylamine (2 equiv.), pre-catalyst **Fe** (2 mol %), Me_3_NO (2.5 mol %), water (2 mL). ^b^ Conversion and selectivity were determined by ^1^H-NMR spectroscopy analysis. ^c^ Isolated yield in the bracket.

**Table 4:** Iron-catalyzed reductive amination with ammonium formate ^a^.

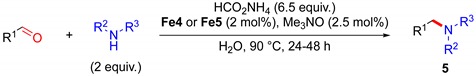
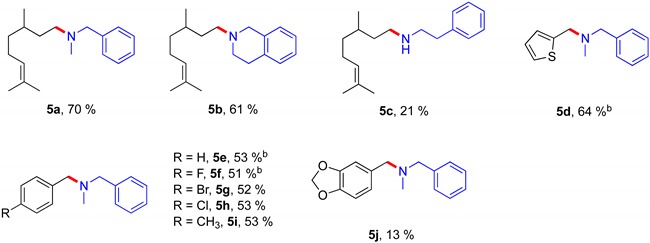

^a^ General conditions: aldehyde (1 mmol), amine (2 equiv.), HCO_2_NH_4_ (6.5 mmol, 6.5 equiv.), pre-catalyst **Fe4** or **Fe5** (2 mol %), Me_3_NO (2.5 mol %), water (2 mL), 90 °C for 24 h. ^b^ for 48 h.

**Table 5:** Recycling of the pre-catalyst **Fe5** ^a,b^.

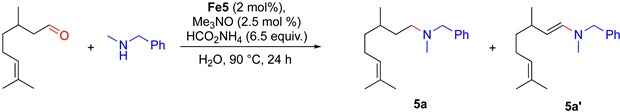			
Entry	Run	Conv. (%) ^c^	Selectivity (5a)/(5a’) ^c^
1	1	96	91/9
2	2	95	96/4
3	3	95	96/4
4	4	96	97/3
5	5	98	96/4

^a^ General conditions for the initial run: citronellal (1 mmol), *N*-methylbenzylamine (2 equiv.), HCO_2_NH_4_ (6.5 equiv.), pre-catalyst **Fe5** (2 mol %), Me_3_NO (2.5 mol %), water (2 mL), 90 °C for 24 h. ^b^ General conditions for run 2–5: citronellal (1 mmol), *N*-methylbenzylamine (2 equiv.), HCO_2_NH_4_ (6.5 equiv.) were added to the former solution, and the mixture was heated to 90 °C. ^c^ Conversion and selectivity were determined by ^1^H-NMR spectroscopy analysis.

## Supplementary Materials

The following are available online. Table S1: Optimization of the reaction conditions for aldehyde reduction by hydride transfer. Table S2: Optimization of the reaction conditions for reductive amination by hydride transfer. Figure S1–S64: The ^1^H, ^13^C, and ^19^F-NMR spectra of compounds.
