# Supplementary material for: Cyclopentadienone Iron Tricarbonyl Complexes-Catalyzed Hydrogen Transfer in Water
Source: Molecules. 2020 Jan 20;25(2):421. doi: 10.3390/molecules25020421 (PMC7024363; doi:10.3390/molecules25020421)
Supplement: Supplementary file 1 [file molecules-25-00421-s001.pdf]

Supporting Information

Cyclopentadienone Iron Tricarbonyl Complexes-Catalyzed Hydrogen Transfer in Water.

Daouda Ndiaye [1,2], Sébastien Coufourier [1], Mbaye Diagne Mbaye [2], Sylvain Gaillard [1] and Jean-Luc Renaud [1,*]

[1] Normandie Univ., LCMT, ENSICAEN, UNICAEN, CNRS, 6 boulevard du Maréchal Juin, 14050 Caen, France; jean-luc.renaud@ensicaen.fr

[2] Université Assane Seck de Ziguinchor, BP 523, Ziguinchor, Sénégal.

Table of contents

Part 1: Scope of the reaction for chemoselective aldehyde reduction and reductive amination by hydride transfer reaction with sodium formate.

Part 2: Synthesis of the water-soluble iron complexes

Part 3: Copies of NMR spectra.

General Considerations: All reactions were carried out using Schlenk or autoclave under an atmosphere of dry Argon. Water was degassed prior to use by bubbling argon gas directly in the solvent. Other solvents were purchased from Carlo Erba and degassed prior to use by freeze-pump thaw procedure (3 times). NMR spectra were recorded on a 400 MHz and 500 MHz Brücker spectrometer. Proton (^{1}H) NMR information is given in the following format: multiplicity: s, singlet; d, doublet; t, triplet; q, quartet; quint, quintuplet; sept, septet; m, multiplet), coupling constant(s) (J) in Hertz (Hz), number of protons. The prefix app is occasionally applied when the true signal multiplicity was unresolved and br indicates the signal in question broadened. Carbon NMR spectra are reported in ppm (δ) relative to $CDCl_3$ (δ 77.16) unless noted otherwise. HRMS analyses were performed by using the LCMT analytical facilities. Neutral activated aluminium oxide was purchased from Alfa Aesar (Brockmann Grade I, 58 Angstroms, -60 Mesh Powder, S.A. 150 m^2/g) and from Merck (Grade I, 90 Angstroms, 70-230 Mesh ASTM). Basic aluminium oxide was purchased from Alfa Aesar (Brockmann GradeI, 58 Angstroms, -60 Mesh Powder, S.A. 150 m^2/g). NMR solvents were filtered through a pad of basic alumina for NMR analysis of iron complexes.

Part 1: Scope of the reaction for reduction of aldehydes and reductive amination by hydride transfer reaction with formate.

- **General procedure A for reduction of aldehydes by hydrogen transfer reaction.**

In a dried flamed Schlenk tube under argon, the corresponding aldehyde (1 equiv.) and sodium formate (170 mg, 5 equiv.) were mixed in water (0.5 M). The iron complex **Fe4** (5 mg, 2 mol %) and Me_3NO (1 mg, 2.5 mol %) were then added. The mixture was stirred and heated at 80 °C for 24 h. After cooling-down to room temperature, the resulting solution was quenched with a saturated aqueous solution of sodium bicarbonate and extracted three times with ethyl acetate. The organic phase was dried over $MgSO_4$, filtrated and concentrated under vacuum to afford the crude product. A purification by flash chromatography on silica gel furnished the alcohol.

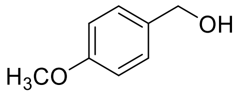

$C_8H_{10}O_2$
Mw: 138.17 g.mol^{-1}

(4-methoxyphenyl)methanol (4a)

Following the general procedure A, starting from 4'-methoxybenzaldehyde (61 µL, 0.5 mmol), alcohol **4a** was obtained as a colorless oil (68 mg, 99 %) after purification by flash column chromatography on silica (eluent: pentane/AcOEt [8:2]).

^{1}H-NMR ($CDCl_3$, 500 MHz) δ 7.30 (d, *J* = 8.3 Hz, 2H), 6.90 (d, *J* = 8.3 Hz, 2H), 4.62 (s, 2H), 3.81 (s, 3H) ppm (OH signal not observed). **^{13}C-NMR ($CDCl_3$, 126 MHz)** δ 159.2, 133.1, 128.7, 114.0, 65.1, 55.3 ppm. Data are in accordance with the literature.[1]

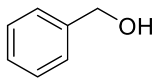

C_7H_8O
Mw: 108.14 g.mol^{-1}

Benzyl alcohol (4b)

Following the general procedure A, starting from benzaldehyde (51 µL, 0.5 mmol), alcohol **4b** was obtained as a colorless oil (52 mg, 96 %) after purification by flash column chromatography on silica (eluent: pentane/AcOEt [8:2]).

^{1}H-NMR ($CDCl_3$, 500 MHz) δ 7.37 (d, *J* = 4.4 Hz, 4H), 7.33-7.29 (m, 1H), 4.70 (s, 2H), 1.70 (s, 1H) ppm. **^{13}C-NMR ($CDCl_3$, 126 MHz)** δ 140.9, 128.6, 127.7, 127.0, 65.4 ppm. Data are in accordance with the literature.[1]

---

[1] Yang, Z.; Zhu, Z.; Luo, R.; Qiu, X.; Liu, J.-T.; Yang, J.-K.; Tang, W. *Green Chem*. **2017**, *19*, 3296.

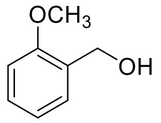

$C_8H_{10}O_2$
Mw: 138.17 g.mol^{-1}

(2-methoxyphenyl)methanol (4c)

Following the general procedure A, starting from 2-methoxybenzaldehyde (68 mg, 0.5 mmol) in an equimolar mixture H_2O/EtOH, alcohol **4c** was obtained as a colorless oil (63 mg, 91 %) after purification by flash column chromatography on silica (eluent: pentane/AcOEt [9:1] then [7:3]).

^{1}H-NMR ($CDCl_3$, 500 MHz) *δ* 7.30-7.26 (m, 2H), 6.95 (t, *J* = 7.4 Hz, 1H), 6.90 (d, *J* = 8.0 Hz, 1H), 4.69 (d, *J* = 5.0 Hz, 2H), 3.88 (s, 3H), 2.30 (s, 1H) ppm. **^{13}C-NMR ($CDCl_3$, 126 MHz)** *δ* 157.5, 129.0, 128.8, 120.7, 110.2, 62.3, 55.3 ppm. Data are in accordance with the literature.[2]

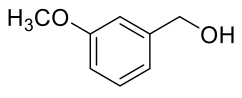

$C_8H_{10}O_2$
Mw: 138.17 g.mol^{-1}

(3-methoxyphenyl)methanol (4d)

Following the general procedure A, starting from 3-methoxybenzaldehyde (61 µL, 0.5 mmol) in an equimolar mixture H_2O/EtOH, alcohol **4d** was obtained as a colorless liquid (67 mg, 97 %) after purification by flash column chromatography on silica (eluent: pentane/AcOEt [9:1]).

^{1}H-NMR ($CDCl_3$, 500 MHz) *δ* 7.29 (t, *J* = 7.9 Hz, 1H), 6.94 (d, *J* = 7.2 Hz, 2H), 6.84 (d, *J* = 8.3 Hz, 1H), 4.68 (s, 2H), 3.82 (s, 3H), 1.66 (s, 1H) ppm. **^{13}C-NMR ($CDCl_3$, 126 MHz)** *δ* 159.9, 142.5, 129.6, 119.1, 113.3, 112.3, 65.3, 55.3 ppm. Data are in accordance with the literature.[2]

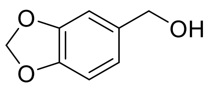

$C_8H_8O_3$
Mw: 152.15 g.mol^{-1}

Piperonyl alcohol (4e)

Following the general procedure A, starting from piperonal (75 mg, 0.5 mmol) in an equimolar mixture H_2O/EtOH, alcohol **4e** was obtained as a white solid (72 mg, 95 %) after purification by flash column chromatography on silica (eluent: pentane/AcOEt [8:2]).

^{1}H-NMR ($CDCl_3$, 500 MHz) *δ* 6.88 (s, 1H), 6.80 (q, *J* = 7.9 Hz, 2H), 5.96 (s, 2H), 4.59 (s, 2H) ppm (OH signal not observed). **^{13}C-NMR ($CDCl_3$, 126 MHz)** *δ* 147.9, 147.1, 134.9, 120.5, 108.2, 107.9, 101.0, 65.3 ppm. Data are in accordance with the literature.[3]

[2] Wu, X.; Corcoran, C.; Yang, S.; Xiao. J. *ChemSusChem* **2008**, *1*, 71.
[3] Ovian, J. M.; Kelly, C. B.; Pistritto, V. A.; Leadbeatter, N. E. *Org. Lett.*, **2017**, *19*, 1286-1289.

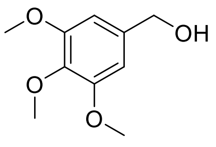

$C_{10}H_{14}O_4$
Mw: 198.22 g.mol^{-1}

(3,4,5-trimethoxyphenyl)methanol (4f)

Following the general procedure A, starting from (3,4,5-trimethoxy)benzaldehyde (98 mg, 0.5 mmol) in an equimolar mixture H_2O/EtOH, alcohol **4f** was obtained as a thick colorless oil (98 mg, 99 %) purification by flash column chromatography on silica (eluent: pentane/AcOEt [8:2]).

^{1}H-NMR ($CDCl_3$, 500 MHz) δ 6.60 (s, 2H), 4.64 (s, 2H), 3.87 (s, 6H), 3.84 (s, 3H), 1.72 (s, 1H) ppm. **^{13}C-NMR ($CDCl_3$, 126 MHz)** δ 153.4, 137.3, 136.6, 103.8, 65.6, 60.9, 56.1 ppm. Data are in accordance with the literature.[4]

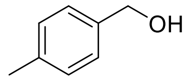

$C_8H_{10}O$
Mw: 122.17 g.mol^{-1}

***p*-tolylmethanol (4g)**

Following the general procedure A, starting from 4-methylbenzaldehyde (59 µL, 0.5 mmol) in an equimolar mixture H_2O/EtOH, alcohol **4g** was obtained as a white solid (59 mg, 96 %) purification by flash column chromatography on silica (eluent: pentane/AcOEt [8:2]).

^{1}H-NMR ($CDCl_3$, 500 MHz) δ 7.19 (d, J = 7.6 Hz, 2H), 7.10 (d, J = 7.6 Hz, 2H), 4.58 (s, 2H), 2.28 (s, 3H) ppm (OH signal not observed). **^{13}C-NMR ($CDCl_3$, 126 MHz)** δ 137.9, 137.4, 129.3, 127.1, 65.3, 21.2 ppm. Data are in accordance with the literature.[2]

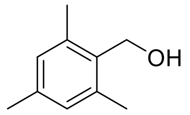

$C_{10}H_{14}O$
Mw: 150.22 g.mol^{-1}

(2,4,6-trimethylphenyl)methanol (4h)

Following the general procedure A, starting from (2,4,6-trimethyl)benzaldehyde (74 µL, 0.5 mmol) in an equimolar mixture H_2O/EtOH, alcohol **4h** was obtained as a white solid (74 mg, 99 %) purification by flash column chromatography on silica (eluent: pentane/AcOEt [8:2]).

^{1}H-NMR ($CDCl_3$, 500 MHz) δ 6.87 (s, 2H), 4.71 (s, 2H), 2.40 (s, 6H), 2.27 (s, 3H), 1.18 (s, 1H) ppm. **^{13}C-NMR ($CDCl_3$, 126 MHz)** δ 137.8, 137.3, 133.7, 129.2, 59.2, 21.0, 19.4 ppm. Data are in accordance with the literature.[5]

---

[4] Elangovan, S.; Garbe, M.; Jiao, H.; Spanneberg, A.; Junge, K.; Beller, M. *Angew. Chem. Int. Ed.* **2016**, *55*, 15364-15368.

[5] Plank, T. N.; Drake, J. L.; Kim, D. K.; Funk, T. W. *Adv. Synth. Catal.* **2012**, *354*, 597.

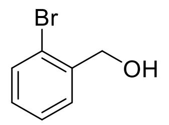

C_7H_7BrO
Mw: 187.04 g.mol^{-1}

(2-bromophenyl)methanol (4i)

Following the general procedure A, starting from 2-bromobenzaldehyde (58 µL, 0.5 mmol), alcohol **1i** was obtained as a white powder (70 mg, 75 %) after purification by flash column chromatography on silica (eluent: pentane/AcOEt [7:3]).

^{1}H-NMR ($CDCl_3$, 500 MHz) *δ* 7.55 (d, *J* = 7.6 Hz, 1H), 7.48 (d, *J* = 7.6 Hz, 1H), 7.34 (t, *J* = 7.6 Hz, 1H), 7.17 (t, *J* = 7.6 Hz, 1H), 4.76 (d, *J* = 5.8 Hz, 2H), 1.97 (t, *J* = 5.8 Hz, 1H) ppm. **^{13}C-NMR ($CDCl_3$, 126 MHz)** *δ* 139.7, 132.6, 129.2, 128.9, 127.7, 122.6, 65.1 ppm. Data are in accordance with the literature.[6]

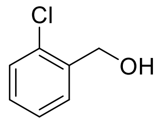

C_7H_7ClO
Mw: 142.58 g.mol^{-1}

(2-chlorophenyl)methanol (4j)

Following the general procedure A, starting from 2-chlorobenzaldehyde (56 µL, 0.5 mmol), alcohol **4j** was obtained as a white powder (66 mg, 92 %) after purification by flash column chromatography on silica (eluent: pentane/AcOEt [7:3]).

^{1}H-NMR ($CDCl_3$, 500 MHz) *δ* 7.48 (d, *J* = 7.4 Hz, 1H), 7.37 (d, *J* = 7.4 Hz, 1H), 7.29 (t, *J* = 7.4 Hz, 1H), 7.24 (t, *J* = 7.4 Hz, 1H), 4.79 (d, *J* = 6.2 Hz, 2H), 1.93 (t, *J* = 6.2 Hz, 1H) ppm. **^{13}C-NMR ($CDCl_3$, 126 MHz)** *δ* 138.2, 132.8, 129.4, 128.9, 128.8, 127.1, 62.9 ppm. Data are in accordance with the literature.[6]

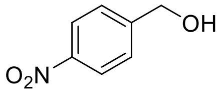

$C_7H_7NO_3$
Mw: 153.14 g.mol^{-1}

(4-nitrophenyl)methanol (4k)

Following the general procedure A, starting from 4-nitrobenzaldehyde (75 mg, 0.5 mmol), alcohol **4k** was obtained as a yellow powder (74 mg, 96 %) after purification by flash column chromatography on silica (eluent: pentane/AcOEt [7:3]).

^{1}H-NMR ($CDCl_3$, 500 MHz) *δ* 8.22 (d, *J* = 8.4 Hz, 2H), 7.54 (d, *J* = 8.4 Hz, 2H), 4.84 (d, *J* = 5.6 Hz, 2H), 1.91 (t, *J* = 5.6 Hz, 1H) ppm. **^{13}C-NMR ($CDCl_3$, 126 MHz)** *δ* 148.1, 147.3, 127.0, 123.8, 64.1 ppm. Data are in accordance with the literature.[6]

[6] Widegren, M. B.; Harkness, G. J.; Slawin, A. M. Z.; Cordes, D. B.; Clarke, M. L. *Angew. Chem. Int. Ed.* **2017**, *56*, 5825.

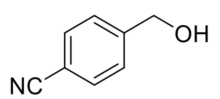

C_8H_7NO
Mw: 133.15 g.mol^{-1}

(4-cyanophenyl)methanol (4l)

Following the general procedure A, starting from 4-cyanobenzaldehyde (65 mg, 0.5 mmol), alcohol **4l** was obtained as a white powder (65 mg, 98 %) after purification by flash column chromatography on silica (eluent: pentane/AcOEt [7:3]).

^{1}H-NMR ($CDCl_3$, 500 MHz) *δ* 7.65 (d, *J* = 8.0 Hz, 2H), 7.48 (d, *J* = 8.0 Hz, 2H), 4.79 (d, *J* = 3.3 Hz, 2H), 1.94 (s, 1H) ppm. **^{13}C-NMR ($CDCl_3$, 126 MHz)** *δ* 146.1, 132.4, 127.0, 118.9, 111.2, 64.3 ppm. Data are in accordance with the literature.[5]

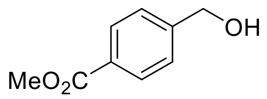

$C_9H_{10}O_3$
Mw: 166.18 g.mol^{-1}

methyl 4-(hydroxymethyl)benzoate (4m)

Following the general procedure A, starting from methyl 4-formylbenzoate (82 mg, 0.5 mmol), alcohol **4m** was obtained as a colorless oil (80 mg, 96 %) after purification by flash column chromatography on silica (eluent: pentane/Et_2O [1:1]).

^{1}H-NMR ($CDCl_3$, 500 MHz) *δ* 8.03 (d, *J* = 8.3 Hz, 2H), 7.43 (d, *J* = 8.3 Hz, 1H), 4.77 (d, *J* = 4.7 Hz, 2H), 3.93 (s, 3H) ppm (OH signal not observed). **^{13}C-NMR ($CDCl_3$, 126 MHz)** *δ* 166.9, 145.9, 129.9, 129.4, 126.5, 64.7, 52.1 ppm. Data are in accordance with the literature.[7]

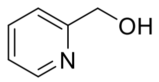

C_6H_7NO
Mw: 109.13 g.mol^{-1}

Pyridin-2-ylmethanol (4n)

Following the general procedure A, starting from 2-pyridinecarboxaldehyde (48 µL, 0.5 mmol), alcohol **4n** was obtained as a colorless oil (45 mg, 82 %) after purification by flash column chromatography on silica (eluent: AcOEt to AcOEt/MeOH [95:5]).

^{1}H-NMR ($CDCl_3$, 500 MHz) *δ* 8.55 (d, *J* = 4.7 Hz, 1H), 7.68 (t, *J* = 7.8 Hz, 1H), 7.25 (d, *J* = 7.8 Hz, 1H), 7.20 (t, J = 4.7 Hz, 1H), 4.76 (s, 2H), 3.95 (s, 1H) ppm. **^{13}C-NMR ($CDCl_3$, 126 MHz)** *δ* 159.0, 148.5, 136.7, 122.4, 120.5, 64.1 ppm. Data are in accordance with the literature.[7]

---

[7] Zhao, M.; Xie, W.; Cui, C. *Chem. Eur. J.* **2014**, *20*, 9259.

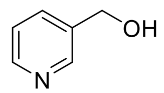

C_6H_7NO
Mw: 109.13 g.mol^{-1}

Pyridin-3-ylmethanol (4o)

Following the general procedure A, starting from 3-pyridinecarboxaldehyde (47 µL, 0.5 mmol), alcohol **4o** was obtained as a colorless oil (41 mg, 75 %) after purification by flash column chromatography on silica (eluent: AcOEt to AcOEt/MeOH [95:5]).

^{1}H-NMR ($CDCl_3$, 500 MHz) δ 8.55 (s, 1H), 8.50 (d, *J* = 3.8 Hz, 1H), 7.73 (d, *J* = 7.7 Hz, 1H), 7.29 (dd, *J* = 7.5, 5.0 Hz, 1H), 4.73 (s, 2H) (OH signal not observed) ppm. **^{13}C-NMR ($CDCl_3$, 126 MHz)** δ 148.8, 148.4, 136.4, 134.9, 123.6, 62.6 ppm. Data are in accordance with the literature.[6]

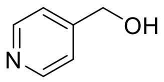

C_6H_7NO
Mw: 109.13 g.mol^{-1}

Pyridin-4-ylmethanol (4p)

Following the general procedure A, starting from 4-pyridinecarboxaldehyde (47 µL, 0.5 mmol), alcohol **1p** was obtained as a white powder (50 mg, 91 %) after purification by flash column chromatography on silica (eluent: AcOEt to AcOEt/MeOH [9:1]).

^{1}H-NMR ($CDCl_3$, 500 MHz) δ 8.49 (d, *J* = 4.8 Hz, 2H), 7.29 (d, *J* = 4.8 Hz, 2H), 4.74 (s, 2H) ppm (OH signal not observed). **^{13}C-NMR ($CDCl_3$, 126 MHz)** δ 150.8, 149.5, 121.2, 63.1 ppm. Data are in accordance with the literature.[8]

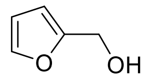

$C_5H_6O_2$
Mw: 98.10 g.mol^{-1}

Furan-2-ylmethanol (4q)

Following the general procedure A, starting from furfural (41 µL, 0.5 mmol), alcohol **4q** was obtained as a yellow oil (48 mg, 98 %) after purification by flash column chromatography on silica (eluent: pentane/AcOEt [7:3]).

^{1}H-NMR ($CDCl_3$, 500 MHz) δ 7.40 (dd, *J* = 1.9, 0.8 Hz, 1H), 6.34 (dd, *J* = 3.2, 1.9 Hz, 1H), 6.30 (d, *J* = 3.2 Hz, 1H), 4.61 (s, 2H), 1.76 (s, 1H) ppm. **^{13}C-NMR ($CDCl_3$, 126 MHz)** δ 153.9, 142.6, 110.4, 107.8, 57.5 ppm. Data are in accordance with the literature.[1]

---

[8] Shi, L.; Liu, Y.; Liu, Q.; Wei, B.; Zhang, G. *Green Chem.* **2012**, *14*, 1372.

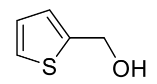

C_5H_6OS
Mw: 114.16 g.mol^{-1}

Thiophen-2-ylmethanol (4r)

Following the general procedure A, starting from 2-thiophenecarboxaldehyde (47 µL, 0.5 mmol), alcohol **4r** was obtained as a colorless oil (52 mg, 91 %) after purification by flash column chromatography on silica (eluent: pentane/AcOEt [7:3]).

^{1}H-NMR ($CDCl_3$, 500 MHz) *δ* 7.29 (d, *J* = 5.0 Hz, 1H), 7.02 (s, 1H), 6.98 (t, *J* = 4.0 Hz, 1H), 4.84 (s, 2H), 1.83 (s, 1H) ppm. **^{13}C-NMR ($CDCl_3$, 126 MHz)** *δ* 144.0, 126.9, 125.7, 125.5, 60.1 ppm. Data are in accordance with the literature.[1]

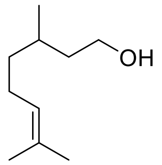

$C_{10}H_{20}O$
Mw: 156.27 g.mol^{-1}

Citronellol (4s)

Following the general procedure A, starting from citronellal (90 µL, 0.5 mmol in an equimolar mixture H_2O/EtOH, alcohol **4s** was obtained as a colorless oil (77 mg, 99 %) without purification.

^{1}H-NMR ($CDCl_3$, 500 MHz) *δ* 5.09 (*app* t, *J* = 7.2 Hz, 1H), 3.67-3.55 (m, 2H), 2.00-1.82 (m, 2H), 1.62 (s, 3H), 1.60-1.45 (m, 5H), 1.38-1.22 (m, 2H), 1.19-1.06 (m, 2H), 0.90 (d, *J* = 7.2 Hz, 3H) ppm. **^{13}C-NMR ($CDCl_3$, 126 MHz)** *δ* 131.3, 124.7, 61.2, 39.9, 37.2, 29.2, 25.7, 25.5, 19.5, 17.7 ppm. Data are in accordance with the literature.[9]

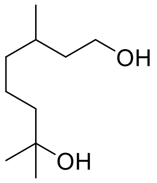

$C_{10}H_{22}O_2$
Mw: 174.28 g.mol^{-1}

3,7-dimethyloctane-1,7-diol (4t)

Following the general procedure A, starting from 7-hydroxycitronellal (93 µL, 0.5 mmol), in an equimolar mixture H_2O/EtOH, diol **4t** was obtained as a thick colorless oil (85 mg, 98 %) without purification.

^{1}H-NMR ($CDCl_3$, 500 MHz) *δ* 3.73-3.64 (m, 2H), 1.64-1.57 (m, 2H), 1.44-1.31 (m, 8H), 1.21 (s, 6H), 1.17-1.13 (m, 1H), 0.90 (d, *J* = 6.6 Hz, 3H) ppm. **^{13}C-NMR ($CDCl_3$, 126 MHz)** *δ* 71.1, 61.2, 44.1, 39.9, 37.6, 29.5, 29.4, 29.2, 21.7, 19.6 ppm. Data are in accordance with the literature.[10]

[9] Mérel, D. S.; Elie, M.; Lohier, J.-F.; Gaillard, S.; Renaud, J.-L. *ChemCatChem*. **2013**, *5*, 2939.
[10] Wang, D.; Shuler, W. G.; Pierce, C. J.; Hilinski, M. K. *Org. Lett.*, **2016**, *18*, 3826.

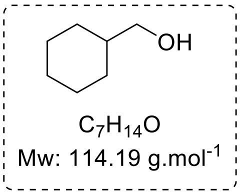

Cyclohexylmethanol (4u)

Following the general procedure A, starting from cyclohexane carboxaldehyde (60 µL, 0.5 mmol) in an equimolar mixture H_2O/EtOH, alcohol **1u** was obtained as a clear yellow oil (56 mg, 98 %) without purification.

^{1}H-NMR ($CDCl_3$, 500 MHz) *δ* 3.44 (d, *J* = 6.4 Hz, 2H), 1.76-1.72 (m, 4H), 1.69-1.66 (m, 1H), 1.51-1.43 (m, 1H), 1.27-1.14 (m, 3H), 0.97-0.88 (m, 2H) ppm (OH signal not observed). **^{13}C-NMR ($CDCl_3$, 126 MHz)** *δ* 68.8, 40.5, 29.6, 26.6, 25.8 ppm. Data are in accordance with the literature.[2]

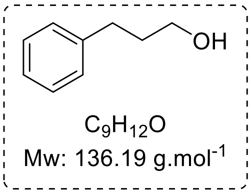

3-phenylpropan-1-ol (4v)

Following the general procedure A, starting from hydrocinnamaldehyde (66 µL, 0.5 mmol) in an equimolar mixture H_2O/EtOH, alcohol **1v** was obtained as a colorless oil (63 mg, 92 %) after purification by flash column chromatography on silica (eluent: pentane/AcOEt [9:1] to [7:3]).

^{1}H-NMR ($CDCl_3$, 500 MHz) *δ* 7.31-7.28 (m, 2H), 7.22-7.18 (m, 3H), 3.69 (*app* t, *J* = 6.1 Hz, 2H), 2.72 (*app* t, *J* = 6.1 Hz, 2H), 1.93-1.88 (m, 2H), 1.25 (s, 1H) ppm. **^{13}C-NMR ($CDCl_3$, 126 MHz)** *δ* 141.8, 128.5, 128.4, 125.9, 62.3, 34.2, 32.1 ppm. Data are in accordance with the literature.[4]

- **General procedure B for reductive amination by hydrogen transfer reaction.**

In a dried flamed Schlenk tube under argon, the aldehyde (1 equiv.), the amine (2 equiv.) and ammonium formate (205 mg, 6.5 equiv.) were mixed in water (0.5 M). The iron complex **Fe5** (5 mg, 2 mol %) and Me_3NO (1 mg, 2.5 mol %) were then added. The mixture was stirred and heated at 90 °C for 24-48 h. After cooling-down to room temperature, the resulting solution was quenched with a saturated aqueous solution of sodium bicarbonate and extracted three times with ethyl acetate. The organic phase was dried over $MgSO_4$, filtrated and concentrated under vacuum to afford the crude product. A purification by flash chromatography on silica gel furnished the amine.

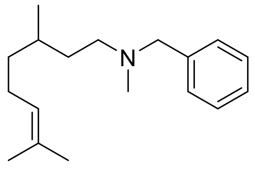

$C_{18}H_{29}N$
Mw: 259.44 g.mol^{-1}

***N*-benzyl-*N*,3,7-trimethyloct-6-en-1-amine (5a)**

Following the general procedure B, starting from citronellal (91 µL, 0.5 mmol) and *N*-methylbenzylamine (150 µL, 1 mmol) for 24 h, the corresponding amine **5a** was obtained as a yellow oil (91 mg, 70 %) after purification by flash column chromatography on silica (eluent: CH_2Cl_2/MeOH [99:1]).

^{1}H-NMR ($CDCl_3$, 400 MHz) *δ* 7.31 (d, *J* = 4.4 Hz, 4H), 7.26-7.22 (m, 1H), 5.09 (t, *J* = 7.1 Hz, 1H), 3.48 (q, *J* = 13.0 Hz, 2H), 2.39 (t, *J* = 7.5 Hz, 2H), 2.18 (s, 3H), 2.04-1.90 (m, 2H), 1.68 (s, 3H), 1.60 (s, 3H), 1.58-1.55 (m, 1H), 1.50-1.46 (m, 1H), 1.34-1.29 (m, 2H), 1.19-1.11 (m, 1H), 0.87 (d, *J* = 6.6 Hz, 3H) ppm. **^{13}C-NMR ($CDCl_3$, 125 MHz)** *δ* 139.3, 131.1, 129.1, 128.2, 126.9, 124.9, 62.4, 55.6, 42.3, 37.3, 34.4, 30.9, 25.8, 25.5, 19.7, 17.7 ppm. Data are in accordance with the literature.[11]

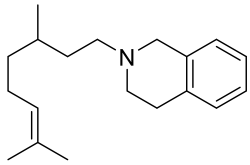

$C_{19}H_{29}N$
Mw: 271.45 g.mol^{-1}

2-(3,7-dimethyloct-6-en-1-yl)-1,2,3,4-tetrahydroisoquinoline (5b)

Following the general procedure B, starting from citronellal (91 µL, 0.5 mmol) and 1,2,3,4-tetrahydroisoquinoline (125 µL, 1 mmol) for 24 h, the corresponding amine **5b** was obtained as a yellow oil (83 mg, 61 %) after purification by flash column chromatography on silica (eluent: pentane/AcOEt [95:5] to [90:10]).

^{1}H-NMR ($CDCl_3$, 500 MHz) *δ* 7.12-7.08 (m, 3H), 7.03-7.01 (m, 1H), 5.11 (t, *J* = 7.1 Hz, 1H), 3.62 (s, 2H), 2.91 (t, *J* = 5.9 Hz, 2H), 2.73 (td, *J* = 6.0, 3.0 Hz, 2H), 2.52 (dt, *J* = 9.5, 5.6 Hz, 2H), 2.06-1.93 (m, 2H), 1.69 (s, 3H), 1.67-1.63 (m, 1H), 1.61 (s, 3H), 1.55-1.48 (m, 1H), 1.45-1.33 (m, 2H), 1.29-1.17 (m, 1H), 0.93 (d, *J* = 6.6 Hz, 3H). **^{13}C-NMR ($CDCl_3$, 125MHz)** *δ* 135.0, 134.4, 131.2, 128.7, 126.6, 126.1, 125.6, 124.9, 56.6, 56.4, 51.1, 37.3, 34.3, 31.2, 29.2, 25.7, 25.5, 19.8, 17.7 ppm. Data are in accordance with the literature.[12]

[11] Moulin, S.; Dentel, H.; Pagnoux-Ozherelyeva, A.; Gaillard, S.; Poater, A.; Cavallo, L.; Lohier, J.- F.; Renaud, J.-L. *Chem. Eur. J.* **2013**, *19*, 17881-17890.

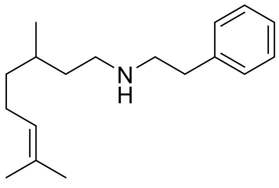

$C_{18}H_{29}N$
Mw: 259.44 g.mol^{-1}

3,7-dimethyl-*N*-phenethyloct-6-en-1-amine[12] (5c)

Following the general procedure B, starting from citronellal (91 µL, 0.5 mmol) and phenethylamine (126 µL, 1 mmol) for 24 h, the corresponding amine **5c** was obtained as a yellow oil (27 mg, 21 %) after purification by flash column chromatography on silica (eluent: CH_2Cl_2/MeOH [98:2]).

^{1}H-NMR ($CDCl_3$, 400 MHz) *δ* 7.33-7.22 (m, 3H), 7.24-7.17 (m, 2H), 5.14-5.03 (m, 1H), 2.94- 2.86 (m, 2H), 2.85-2.77 (m, 2H), 2.70-2.56 (m, 2H), 2.04-1.88 (m, 2H), 1.68 (s, 3H), 1.59 (s, 3H), 1.54-1.42 (m, 2H), 1.40-1.21 (m, 3H), 1.20-1.09 (m, 1H), 0.87 (d, J = 6.5 Hz, 3H) ppm. **^{13}C-NMR ($CDCl_3$, 125 MHz)** *δ* 140.0, 131.2, 128.7, 128.4, 126.1, 124.7, 51.2, 47.8, 37.1, 36.3, 30.6, 25.7, 25.4, 19.6, 17.6 ppm. Data are in accordance with the literature.[11]

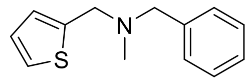

$C_{13}H_{15}NS$
Mw: 217.33 g.mol^{-1}

***N*-benzyl-*N*-methyl-1-(thiophen-2-yl)methanamine (5d)**

Following the general procedure B, starting from 2-thiophenecarboxaldehyde (47 µL, 0.5 mmol) and *N*-methylbenzylamine (150 µL, 1 mmol) for 48 h, the corresponding amine **5d** was obtained as a yellow oil (69 mg, 64 %) after purification by flash column chromatography on silica (eluent: pentane/AcOEt [95:5] to [90:10]).

^{1}H-NMR ($CDCl_3$, 500 MHz) *δ* 7.27 (d, *J* = 7.5 Hz, 2H), 7.22 (t, *J* = 7.4 Hz, 2H), 7.16-7.13 (m, 2H), 6.85-6.84 (m, 1H), 6.83 (s, 1H), 3.66 (s, 2H), 3.44 (s, 2H), 2.15 (s, 3H) ppm. **^{13}C-NMR ($CDCl_3$, 125 MHz)** *δ* 142.9, 139.0, 128.9, 128.3, 127.0, 126.4, 125.7, 124.9, 61.1, 56.1, 42.2 ppm. **IR (neat)** *ν* 3027, 2924, 2837, 2785, 1495, 1452, 1418, 1366, 1340, 1277, 1253, 1222, 1168, 1130, 1075, 1023, 973, 908, 863, 820, 736, 694, 606, 502, 479 cm^{-1}. **HRMS (m/z)**: $[M+H]^+$ calculated for $C_{13}H_{16}NS$: 218.1003; found: 218.1009.

[12] Pagnoux-Ozherelyeva, A.; Pannetier, N.; Mbaye, M. D.; Gaillard, S.; Renaud, J.-L. *Angew. Chem. Int. Ed.* **2012**, *51*, 4976–4980.

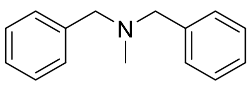

$C_{15}H_{17}N$
Mw: 211.31 g.mol^{-1}

_N_-benzyl-_N_-methyl-1-phenylmethanamine (5e)

Following the general procedure B, starting from benzaldehyde (51 µL, 0.5 mmol) and *N*-methylbenzylamine (150 µL, 1 mmol) for 48 h, the corresponding amine **5d** was obtained as a yellow oil (56 mg, 53 %) after purification by flash column chromatography on silica (eluent: pentane/AcOEt [97:3]).
^{1}H-NMR ($CDCl_3$, 500 MHz) *δ* 7.37 (d, *J* = 7.4 Hz, 4H), 7.32 (t, *J* = 7.4 Hz, 4H), 7.25 (t, *J* = 7.4 Hz, 2H), 3.53 (s, 4H), 2.19 (s, 3H) ppm. **^{13}C-NMR ($CDCl_3$, 125 MHz)** *δ* 139.4, 128.9, 128.2, 126.9, 61.9, 42.3 ppm. Data are in accordance with the literature.[13]

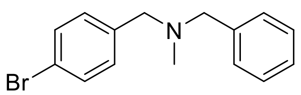

$C_{15}H_{16}BrN$
Mw: 290.20 g.mol^{-1}

_N_-benzyl-1-(4-bromophenyl)-_N_-methylmethanamine[14] (5f)

Following the general procedure B, starting from 4-bromobenzaldehyde (93 mg, 0.5 mmol) and *N*-methylbenzylamine (150 µL, 1 mmol) for 24 h, the corresponding amine **5f** was obtained as a yellow oil (75 mg, 52 %) after purification by flash column chromatography on silica (eluent: pentane/AcOEt [98:2] to [95:5]).
^{1}H-NMR ($CDCl_3$, 500 MHz) *δ* 7.37 (d, *J* = 8.05 Hz, 2H), 7.27 (m, 2H), 7.18 (m, 5H), 3.44 (s, 2H), 3.39 (s, 2H), 2.09 (s, 3H) ppm. **^{13}C-NMR ($CDCl_3$, 125 MHz)** *δ* 131.3, 130.6, 128.9, 128.3, 127.0, 61.9, 61.1, 42.2 ppm. Data are in accordance with the literature.[13]

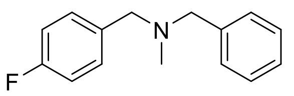

$C_{15}H_{16}FN$
Mw: 229.30 g.mol^{-1}

_N_-benzyl-1-(4-fluorophenyl)-_N_-methylmethanamine[11] (5g)

Following the general procedure B, starting from 4-fluorobenzaldehyde (54 µL, 0.5 mmol) and *N*-methylbenzylamine (150 µL, 1 mmol) for 24 h, the corresponding amine **5f** was obtained as a yellow oil (58 mg, 51 %) after purification by flash column chromatography on silica (eluent: pentane/AcOEt [95:5] to [90:10]).
^{1}H-NMR ($CDCl_3$, 500 MHz) *δ* 7.28-7.23 (m, 6H), 7.18-7.16 (m, 1H), 6.92 (t, *J* = 8.3 Hz, 2H), 3.43 (s, 2H), 3.40 (s, 2H), 2.09 (s, 3H) ppm. **^{13}C-NMR ($CDCl_3$, 125 MHz)** *δ* 162.9, 161.0, 139.2, 135.1,

---

[13] Kirinde Arachchige, P. T.; Lee, H.; Yi, C. S. *J. Org. Chem.* **2018**, *83*, 4932-4947.

[14] Milne, K.; Sun, J.; Zaal, E. A.; Mowat, J.; Celie, P. H. N. Fish, A.; Berkers,C. R.; Forlanif, G.; Loayza-Puch, F.; Jamieson, C.; Agami, R. *Bioorg. Med. Chem. Letters* **2019**, *29*, 2626-2631.

130.4, 130.3, 128.9, 128.3, 127.0, 115.1, 114.9, 61.9, 61.1, 42.2 ppm. **^{19}F-NMR ($CDCl_3$, 470 MHz)** δ -116.16 ppm. Data are in accordance with the literature.[15]

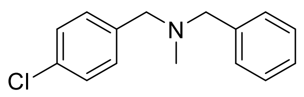

$C_{15}H_{16}ClN$
Mw: 245.75 g.mol^{-1}

_N_-benzyl-1-(4-chlorophenyl)-_N_-methylmethanamine (5h)

Following the general procedure B, starting from 4-chlorobenzaldehyde (70 mg, 0.5 mmol) and *N*-methylbenzylamine (150 µL, 1 mmol) for 24 h, the corresponding amine **5h** was obtained as a yellow oil (65 mg, 53 %) after purification by flash column chromatography on silica (eluent: pentane/AcOEt [95:5] to [90:10]).

^{1}H-NMR ($CDCl_3$, 500 MHz) δ 7.32-7.21 (m, 10H), 3.48 (s, 2H), 3.45 (s, 2H), 2.14 (s, 3H) ppm. **^{13}C-NMR ($CDCl_3$, 125 MHz)** δ 139.1, 137.9, 132.6, 130.2, 128.9, 128.4, 128.3, 127.1, 61.9, 61.1, 42.2 ppm. **IR (neat)** *v* 3028, 2924, 2840, 2786, 1598, 1490, 1452, 1406, 1364, 1284, 1263, 1240, 1192, 1132, 1088, 1025, 1015, 979, 939, 908, 867, 838, 799, 737, 697, 671, 634, 611, 536, 488, 456 cm^{-1}. **HRMS (m/z)**: $[M+H]^+$ calculated for $C_{15}H_{17}ClN$: 246.1050; found: 246.1052.

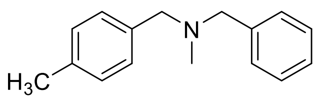

$C_{16}H_{19}N$
Mw: 225.33 g.mol^{-1}

_N_-benzyl-_N_-methyl-1-(_p_-tolyl)methanamine[13] (5i)

Following the general procedure B, starting from *p*-tolualdehyde (59 µL, 0.5 mmol) and *N*-methylbenzylamine (150 µL, 1 mmol) for 24 h, the corresponding amine **5i** was obtained as a yellow oil (50 mg, 53 %) after purification by flash column chromatography on silica (eluent: pentane/AcOEt [95:5] to [90:10]).

^{1}H-NMR ($CDCl_3$, 500 MHz) δ 7.27-7.24 (m, 3H), 7.18 (d, J = 7.9 Hz, 4H), 7.06 (d, J = 7.6 Hz, 2H), 3.43 (d, 4H), 2.26 (s, 3H), 2.09 (s, 3H) ppm. **^{13}C-NMR ($CDCl_3$, 125 MHz)** δ 139.4, 136.5, 136.2, 128.9, 128.2, 126.9, 61.8, 61.6, 42.2, 21.1 ppm. Data are in accordance with the literature.[13]

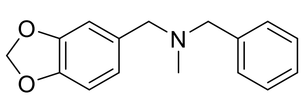

$C_{16}H_{17}NO_2$
Mw: 255.32g.mol^{-1}

1-(benzo[_d_][1,3]dioxol-5-yl)-_N_-benzyl-_N_-methylmethanamine[16] (5j)

[15] Kazuya, Y.; Jinling, H.; Takamishi, O.; Noritaka, M. *Chem. Eur. J.*, **2010**, *16*, 7199-7204

[16] Jung, Y. J.; Bae, J. W.; Park, E. S.; Chang, Y. M.; Yoon, C. M. *Tetrahedron* **2003**, *59*, 10331-10338.

Following the general procedure B, starting from piperonal (75 mg, 0.5 mmol) and *N*-methylbenzylamine (150 µL, 1 mmol) for 24 h, the corresponding amine **5j** was obtained as a yellow oil (17 mg, 13 %) after purification by flash column chromatography on silica (eluent: pentane/AcOEt [95:5] to [90:10]).

^{1}H-NMR ($CDCl_3$, 500 MHz) *δ* 7.36-7.31 (m, 4H), 7.26-7.23 (m, 1H), 6.91 (s, 1H), 6.77 (q, *J* = 7.9 Hz, 2H), 5.9 (s, 2H), 3.51 (s, 2H), 3.43 (s, 2H), 2.17 (s, 2H) ppm. **^{13}C-NMR ($CDCl_3$, 125 MHz)** *δ* 147.7, 146.5, 139.4, 133.4, 128.9, 128.3, 126.9, 121.9, 109.3, 107.9, 100.9, 61.8, 61.6, 42.1 ppm. Data are in accordance with the literature.

Part 2: Preparation of water-soluble complexes

- **Preparation of *N*[1],*N*[2]-bisalkylethane-1,2-diamine derivatives.**

General procedure C:[17] To a stirring mixture of diethyl oxalate (1 equiv.) in absolute ethanol (C = 0.3 M) was added the corresponding amine (2 equiv.). The mixture was stirred and heated under reflux for 2 hours. The resulting mixture was cooled-down to 0°C and the precipitated solid was isolated by filtration. The solid was then washed with cold diethylether and dried under vacuum to afford the pure product.

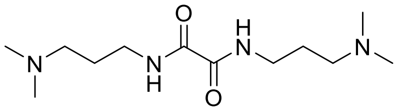

$C_{12}H_{26}N_4O_2$
Mw : 258.37 g.mol^{-1}

***N*[1],*N*[2]-bis(3-(dimethylamino)propyl)oxalamide (5).** Following the general procedure C, starting from diethyloxalate (1.9 mL, 13.4 mmol) and *N*,*N*-dimethyl-1,3-propanediamine (3.4 mL, 27.4 mmol), **5** was obtained as a white powder (3.54 g, 100 %).
^{1}H-NMR ($CDCl_3$, 400 MHz) *δ* 8.41 (s, 2H), 3.37 (q, *J* = 6.4 Hz, 4H), 2.36 (t, *J* = 6.4 Hz, 4H), 2.22 (s, 12H), 1.69 (p, *J* = 6.4 Hz, 4H) ppm. **^{13}C-NMR ($CDCl_3$, 100 MHz)** *δ* 160.0, 58.1, 45.4, 39.0, 26.3 ppm. **IR (neat)** *v* 3291, 2812, 2765, 1650, 1523, 1465, 1434, 1377, 1303, 1276, 1240, 1214, 1147, 1129, 1111, 1071, 1040, 1017, 985, 919, 888, 863, 798, 784, 761 cm^{-1}. **HRMS (m/z)**: $[M+H]^+$ calculated for $C_{12}H_{27}N_4O_2$: 259.2134; found: 259.2135.

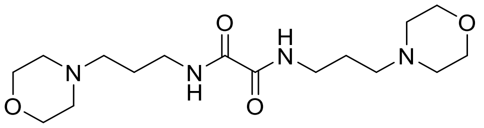

$C_{16}H_{30}N_4O_4$
Mw : 342.44 g.mol^{-1}

***N*[1],*N*[2]-bis(3-morpholinopropyl)oxalamide (6).** Following the general procedure C, starting from diethyloxalate (1.9 mL, 13.4 mmol) and 3-morpholinopropylamine (4.0 mL, 27.4 mmol), **1d** was obtained as a white needles (4.7 g, 100 %).

---

[17] Rice, L. M.; Grogan, C. H.; Reid, E. E. *J. Am. Chem. Soc.*, **1953**, *75*, 242–243.

^{1}H-NMR ($CDCl_3$, 400 MHz) *δ* 8.58 (s, 2H), 3.77 (t, *J* = 6.1 Hz, 8H), 3.41 (q, *J* = 6.1 Hz, 4H), 2.48-2.45 (m, 12H), 1.72 (q, *J* = 6.1 Hz, 6H) ppm. **^{13}C-NMR ($CDCl_3$, 100 MHz)** *δ* 160.0, 66.9, 57.7, 53.8, 39.4, 24.8 ppm. **IR (neat)** *ν* 3289, 2973, 2937, 2873, 2811, 2766, 1647, 1522, 1465, 1434, 1377, 1303, 1275, 1254, 1240, 1214, 1147, 1129, 1112, 1070, 1039, 1017, 985, 919, 888, 863, 979, 783, 762, 734 cm^{-1}. **HRMS (m/z)**: $[M+H]^+$ calculated for $C_{16}H_{31}N_4O_4$: 343.2345; found: 343.2346.

General procedure D:[18] To a stirred solution of the corresponding amide (1 equiv.) in THF (0.3 M) at 0°C, was added by portions $LiAlH_4$ (5-8 equiv.) under an argon atmosphere at such a rate that the temperature was maintained at 0 °C. The resulting suspension was stirred and heated at reflux for 2h. After cooling to 0 °C, dichloromethane (0.3 mL/mmol) was added followed by a dropwise addition of an aqueous saturated solution of Na_2SO_4 until gas evolution ceased and apparition of white salts. The solids were removed by filtration through Celite® and the filter cake was washed with a (9:1) mixture of CH_2Cl_2-MeOH. The filtrate was dried with $MgSO_4$, filtrated and evaporated under reduced pressure to give the pure product.

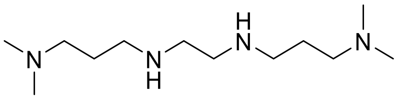

$C_{12}H_{30}N_4$
Mw : 230.40 g.mol^{-1}

***N*^{1},*N*$^{1'}$-(ethane-1,2-diyl)bis(*N*^{3},*N*^{3}-dimethylpropane-1,3-diamine) (3).** Following the general procedure D, starting from oxalamide **5** (2.0 g, 7.7 mmol) and lithium aluminium hydride (1.47 g, 38.7 mmol), **3** was obtained as a colorless liquid (1.65 g, 93 %).

^{1}H-NMR ($CDCl_3$, 400 MHz) *δ* 2.72 (s, 4H), 2.64 (t, *J* = 7.1 Hz, 4H), 2.30 (t, *J* = 7.1 Hz, 4H), 2.21 (s, 12H), 1.65 (p, *J* = 7.1 Hz, 4H) ppm (NH signal not observed). **^{13}C-NMR ($CDCl_3$, 100 MHz)** *δ* 58.0, 49.5, 48.4, 45.6, 28.2 ppm. **IR (neat)** *ν* 2816, 2770, 1632, 1606, 1486, 1461, 1374, 1318, 1300, 1167, 1126, 1099, 1014, 908, 844, 731 cm^{-1}. **HRMS (m/z)**: $[M+H]^+$ calculated for $C_{12}H_{31}N_4$: 231.2549; found: 231.2553.

---

[18] Bilke, J. L.; O'Brien, P. *J. Org. Chem.*, **2008**, *73*, 6452-6454.

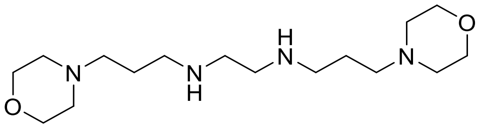

***N*[1],*N*[2]-bis(3-morpholinopropyl)ethane-1,2-diamine (4).** Following the general procedure D, starting from oxalamide **6** (2.0 g, 5.8 mmol) and lithium aluminium hydride (1.11 g, 29.2 mmol), **4** was obtained as a colorless oil (1.75 g, 95 %).

^{1}H-NMR ($CDCl_3$, 400 MHz) δ 3.72-3.69 (m, 8H), 2.71 (s, 4H), 2.65 (t, *J* = 7.0 Hz, 4H), 2.42-2.37 (m, 14H), 1.68 (q, *J* = 7.0 Hz, 4H) ppm. **^{13}C-NMR ($CDCl_3$, 100 MHz)** δ 67.0, 57.3, 53.8, 49.6, 48.5, 27.0 ppm. **IR (neat)** ν 2814, 1658, 1632, 1606, 1488, 1449, 1424, 1372, 1318, 1167, 1117, 1011, 906, 729 cm^{-1}. **HRMS (m/z)**: $[M+H]^+$ calculated for $C_{16}H_{35}N_4O_2$: 315.2760; found: 315.2762.

- **Preparation of water-soluble complexes.**

General procedure E:[19] In a Schlenk tube, diphenylcyclopentanetrione (1 equiv.) and *N,N'*-dimethylethylenediamine (1 equiv.) were introduced in methanol (C = 0.5 M). The reaction mixture was heated under reflux overnight. After complete consumption of the starting materials, the solvent was removed under vacuum. Purification by flash chromatography on alumina with dichlomethane, and precipitation in a mixture of Et_2O/pentane (1:1) furnish the pure product as a purple powder.

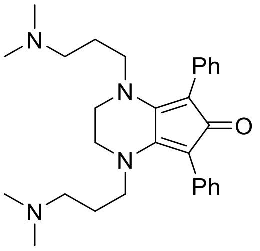

1,4-bis(3-(dimethylamino)propyl)-5,7-diphenyl-1,2,3,4-tetrahydro-6*H*-cyclopenta[*b*]pyrazin-6-one (1). Following the general procedure E, starting from

[19] Haak, E. *Eur. J. Org. Chem.* **2007**, *17*, 2815-2824.

diphenylcyclopentanetrione (1.83 g, 6.9 mmol) and **3** (1.60 g, 6.9 mmol), ligand **1** was obtained as a dark purple powder (2.50 g, 79 %).

^{1}H-NMR ($CDCl_3$, 400 MHz) *δ* 7.31-7.23 (m, 8H), 7.19-7.15 (m, 2H), 3.38 (s, 4H), 3.24-3.20 (m, 4H), 2.04 (s, 12H), 1.73-1.69 (m, 4H), 1.54-1.47 (m, 4H) ppm. **^{13}C-NMR ($CDCl_3$, 100 MHz)** *δ* 195.4, 149. 5, 134.0, 131.4, 127.4, 125.8, 98.7, 56.4, 50.9, 48.3, 45.3, 24.3 ppm. **IR (neat)** *ν* 3289, 2938, 2812, 2783, 1651, 1582, 1523, 1492, 1464, 1456, 1429, 1371, 1349, 1302, 1275, 1215, 1197, 1156, 1140, 1110, 1040, 1017, 985, 944, 919, 889, 863, 836, 824, 806, 766, 757, 727, 968 cm^{-1}. **HRMS (m/z)**: $[M+H]^+$ calculated for $C_{29}H_{39}N_4O$: 459.3124; found: 459.3124.

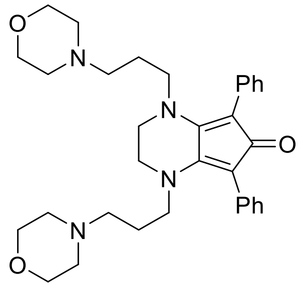

$C_{33}H_{42}N_4O_3$
Mw : 542.72 $g.mol^{-1}$

1,4-bis(3-morpholinopropyl)-5,7-diphenyl-1,2,3,4-tetrahydro-6*H*-cyclopenta[*b*]pyrazin-6-one (2). Following the general procedure F, starting from diphenylcyclopentanetrione (1.47 g, 5.6 mmol) and **4** (1.75 g, 5.6 mmol), ligand **2** was obtained as a purple powder (2.66 g, 88 %). **^{1}H-NMR ($CDCl_3$, 400 MHz)** *δ* 7.31-7.22 (m, 8H), 7.18-7.14 (m, 2H), 3.59 (t, *J* = 4,4 Hz, 8H), 3.39 (s, 4H), 3.26 (t, *J* = 7.8 Hz, 4H), 2.17 (s, 8H), 1.81 (t, *J* = 7.2 Hz, 4H), 1.58-1.49 (m, 4H) ppm. **^{13}C-NMR ($CDCl_3$, 100 MHz)** *δ* 195.3, 149.5, 133.9, 131.3, 127.4, 125.8, 98.7, 66.9, 55.7, 53.5, 51.1, 48.3, 23.2 ppm. **IR (neat)** *ν* 3291, 2938, 2874, 2811, 2766, 2325, 2111, 1794, 1651, 1603, 1523, 1466, 1434, 1377, 1361, 1303, 1276, 1240, 1214, 1169, 1147, 1129, 1112, 1071, 1039, 1017, 985, 919, 888, 863, 798, 784, 762 cm^{-1}. **HRMS (m/z)**: $[M+H]^+$ calculated for $C_{33}H_{43}N_4O_3$: 543.3335; found: 543.3333.

General procedure F: In a dried Schlenk tube under argon, the cyclopentadienone derivative (1 equiv.) and $Fe_2(CO)_9$ (2 equiv.) were introduced in dry and degassed toluene (C = 0.3 M). The reaction mixture was stirred overnight at 110°C. The resulting mixture was cooling down to room temperature and purified on neutral alumina oxide column chromatography

surrounded by a pad of Celite® (eluent: CH_2Cl_2 to CH_2Cl_2/MeOH [98:2]). Precipitation on diethylether/pentane (1:1) and filtration under vacuum furnish the pure product.

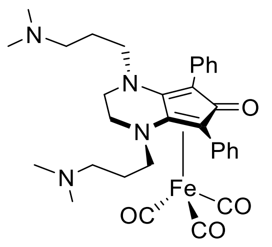

$C_{32}H_{38}FeN_4O_4$
Mw : 598.53 g.mol^{-1}

Iron complex (Fe6). Following the general procedure F, starting from cyclopentadienone ligand **1** (1.0 g, 2.2 mmol) and $Fe_2(CO)_9$ (1.59 g, 4.4 mmol), iron complex **Fe6** was obtained as a yellow powder (625 mg, 48 %).

^{1}H-NMR ($CDCl_3$, 400 MHz) *δ* 7.53 (d, *J* = 7.1 Hz, 4H), 7.41-7.28 (m, 6H), 3.56-3.50 (m, 2H), 2.96-2.84 (m, 4H), 2.40-2.31 (m, 2H), 2.09 (s, 12H), 1.81-1.69 (m, 4H), 1.55-1.52 (m, 2H), 1.41-1.36 (m, 2H) ppm. **^{13}C-NMR ($CDCl_3$, 100 MHz)** *δ* 210.2, 166.0, 132.2, 132.1, 128.4, 127.9, 114.4, 71.0, 56.5, 50.8, 47.2, 45.4, 25.5 ppm. **IR (neat)** *ν* 2815, 2775, 2724, 2032, 1961, 1919, 1628, 1608, 1578, 1526, 1493, 1455, 1439, 1357, 1317, 1287, 1168, 1154, 1097, 1071, 1040, 950, 890, 853, 821, 749, 731, 698 cm^{-1}. **HRMS (m/z)**: $[M+H]^+$ calculated for $C_{32}H_{39}N_4O_4Fe$: 599.2321; found: 599.2322. Anal. Calcd. for $C_{32}H_{39}N_4O_4Fe$: C, 64.22; N, 9.24; H, 6.40. Found: C, 63.87; N, 9.24; H, 6.54.

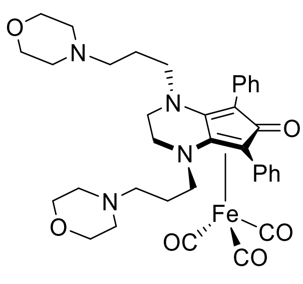

$C_{36}H_{42}FeN_4O_6$
Mw : 682.60 g.mol^{-1}

Iron complex (Fe7). Following the general procedure F, starting from cyclopentadienone ligand **2** (300 mg, 0.55 mmol) and $Fe_2(CO)_9$ (402 mg, 1.1 mmol), iron complex **Fe7** was obtained as a yellow powder (285 mg, 76 %).

^{1}H-NMR ($CDCl_3$, 400 MHz) *δ* 7.53 (d, *J* = 7.1 Hz, 4H), 7.40-7.31 (m, 6H), 3.60 (t, *J* = 4.2 Hz, 8H), 3.53-3.48 (m, 2H), 2.96-2.87 (m, 4H), 2.41-2.30 (m, 2H), 2.30-2.14 (m, 8H), 1.92-1.76 (m, 4H),

1.60-1.48 (m, 2H), 1.48-1.35 (m, 2H) ppm. **^{13}C-NMR ($CDCl_3$, 100 MHz)** δ 210.1, 165.9, 132.1, 132.1, 128.4, 127.9, 114.4, 70.9, 66.9, 55.7, 53.6, 51.2, 47.2, 24.3 ppm. **IR (neat)** ν 2951, 2888, 2805, 2326, 2025, 1967, 1946, 1619, 1560, 1493, 1469, 1445, 1363, 1316, 1304, 1282, 1180, 1146, 1114, 1070, 1024, 1004, 969, 948, 917, 879, 856, 801, 758, 728, 703 cm^{-1}. **HRMS (m/z)**: $[M+H]^+$ calculated for $C_{36}H_{43}N_4O_6Fe$: 683.2532; found: 683.2529. Analysis of **Fe3d** CIF file (see below) showed that 1 molecule of water crystallized with 4 molecules of complex. Anal. Calcd. for $C_{36}H_{43}N_4O_6Fe.(H_2O)_{0.25}$: C, 62.93; N, 8.15; H, 6.23. Found: C, 63.10; N, 8.35; H, 6.41.

General procedure G: In a two necked round bottom flask under argon atmosphere, the corresponding iron complex (**Fe6** or **Fe7**, 1 equiv.) was introduced in dry dichloromethane (C = 0.3 mL). Methyl iodide (2-3 equiv.) was added dropwise and the resulting mixture was stirred overnight at room temperature. The solvent was partially removed and a mixture of pentane/Et_2O (50:50) was added resulting in precipitation. Filtration afforded the pure cationic complex.

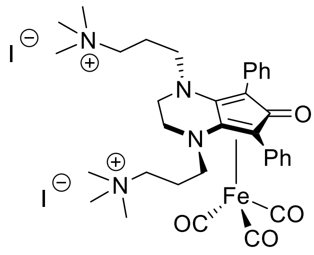

$C_{34}H_{44}FeI_2N_4O_4$
Mw : 882.40 g.mol^{-1}

Water-soluble iron complex (Fe4). Following the general procedure G, starting from iron complex **Fe6** (100 mg, 0.17 mmol) and iodomethane (33 µL, 51 mmol), **Fe4** was obtained as a yellow powder (144 mg, 98 %).

^{1}H-NMR (D_2O, 600 MHz) δ 7.64 (d, *J* = 6.4 Hz, 4H), 7.56-7.52 (m, 6H), 3.56-3.54 (m, 2H), 3.05-3.03 (m, 2H), 2.94 (s, 18H), 2.90-2.85 (m, 2H), 2.74-2.69 (m, 2H), 2.65 (ddd, *J* = 13.9, 9.3, 4.9 Hz, 2H), 2.53 (td, *J* = 12.5, 4.4 Hz, 2H), 1.90-1.81 (m, 2H), 1.71-1.57 (s *br*, 2H) ppm. **^{13}C-NMR (D_2O, 151 MHz)** δ 161.3, 132.6, 130.5, 129.1, 129.0, 114.2, 72.0, 63.5, 52.9, 48.8, 46.7, 20.8 ppm (CO ligands signal missing). **IR (neat)** ν 3010, 2804, 2038, 1955, 1615, 1538, 1490, 1474, 1456, 1439, 1365, 1304, 1287, 1264, 1206, 1175, 1142, 1106, 1075, 1053, 1002, 965, 917, 870, 813, 797, 779, 757, 729, 703, 657 cm^{-1}. **HRMS (m/z)**: $[M]^{2+}$ calculated for $C_{34}H_{44}FeN_4O_4$: 314.1356; found: 314.1350.

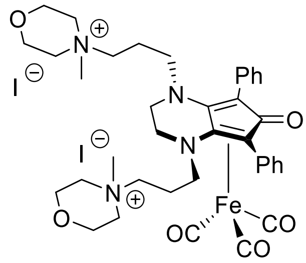

$C_{38}H_{48}FeI_2N_4O_6$

Mw : 966.48 g.mol^{-1}

Water-soluble iron complex (Fe5). Following the general procedure H, starting from iron complex **Fe7** (100 mg, 0.15 mmol) and iodomethane (27 µL, 0.45 mmol), **Fe5** was obtained as a yellow powder (145 mg, 100 %).

^{1}H-NMR (D_2O, 600 MHz) *δ* 7.67 (d, *J* = 6.4 Hz, 4H), 7.56-7.53 (m, 6H), 3.97-3.95 (m, 4H), 3.91-3.89 (m, 2H), 3.56 (d, *J* = 6.0 Hz, 2H), 3.31-3.24 (m, 8H), 3.05-3.00 (m, 2H), 3.00 (s, 6H), 2.95-2.90 (m, 2H), 2.86 (td, *J* = 12.6, 4.4 Hz, 2H), 2.73-2.67 (m, 4H), 1.92-1.81 (m, 2H), 1.73-1.61 (s *br*, 1H) ppm. **^{13}C-NMR (D_2O, 151 MHz)** *δ* 161.4, 132.7, 130.6, 129.1, 129.1, 114.2, 71.9, 60.2, 60.2, 59.6, 59.6, 48.8, 46.7, 19.4 ppm (CO ligands signal missing). **IR (neat)** *ν* 2873, 2034, 1957, 1621, 1533, 1495, 1452, 1441, 1362, 1260, 1203, 1119, 1074, 1018, 964, 922, 904, 876, 849, 759, 731, 705, 625, 615, 586, 554, 528 cm^{-1}. **HRMS (m/z)**: $[M]^{2+}$ calculated for $C_{38}H_{48}N_4O_6Fe$: 356.1462; found: 356.1468.

Part 3: Copies of NMR spectra.

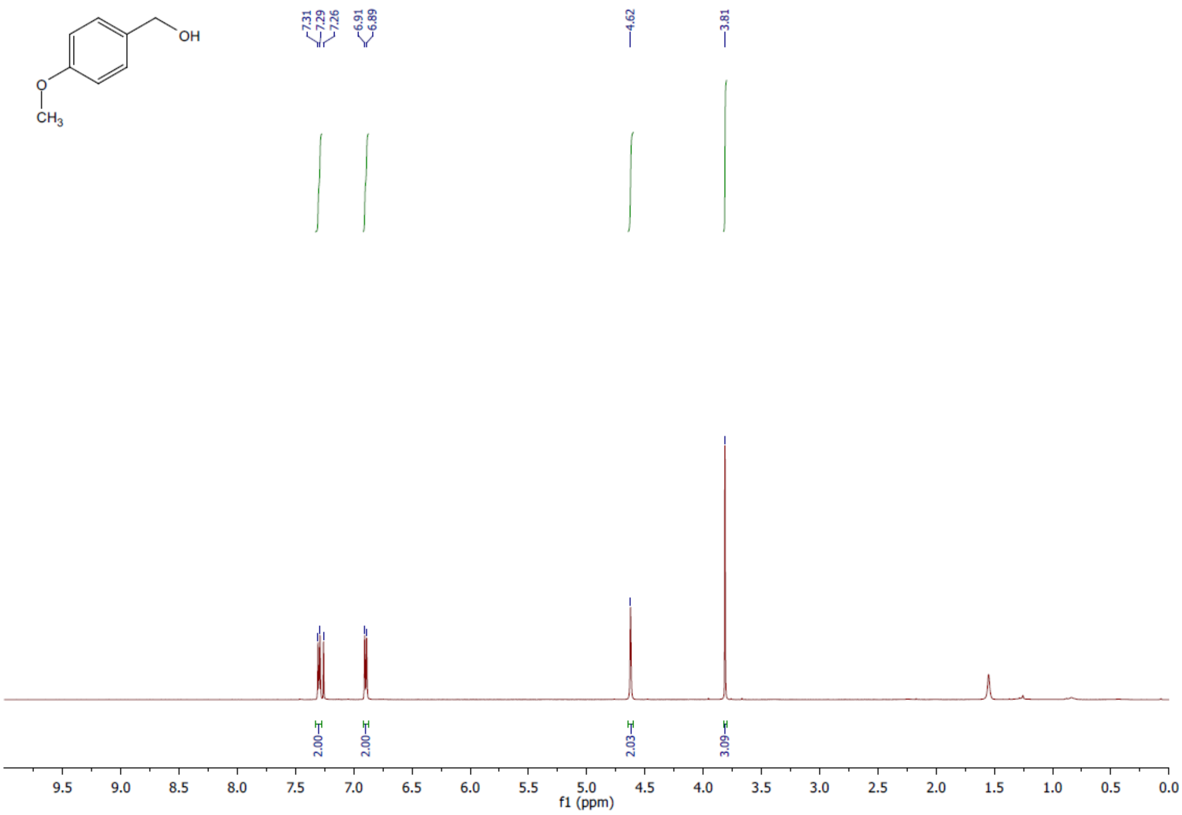

Figure S1. ^{1}H-NMR spectrum of alcohol **4a**.

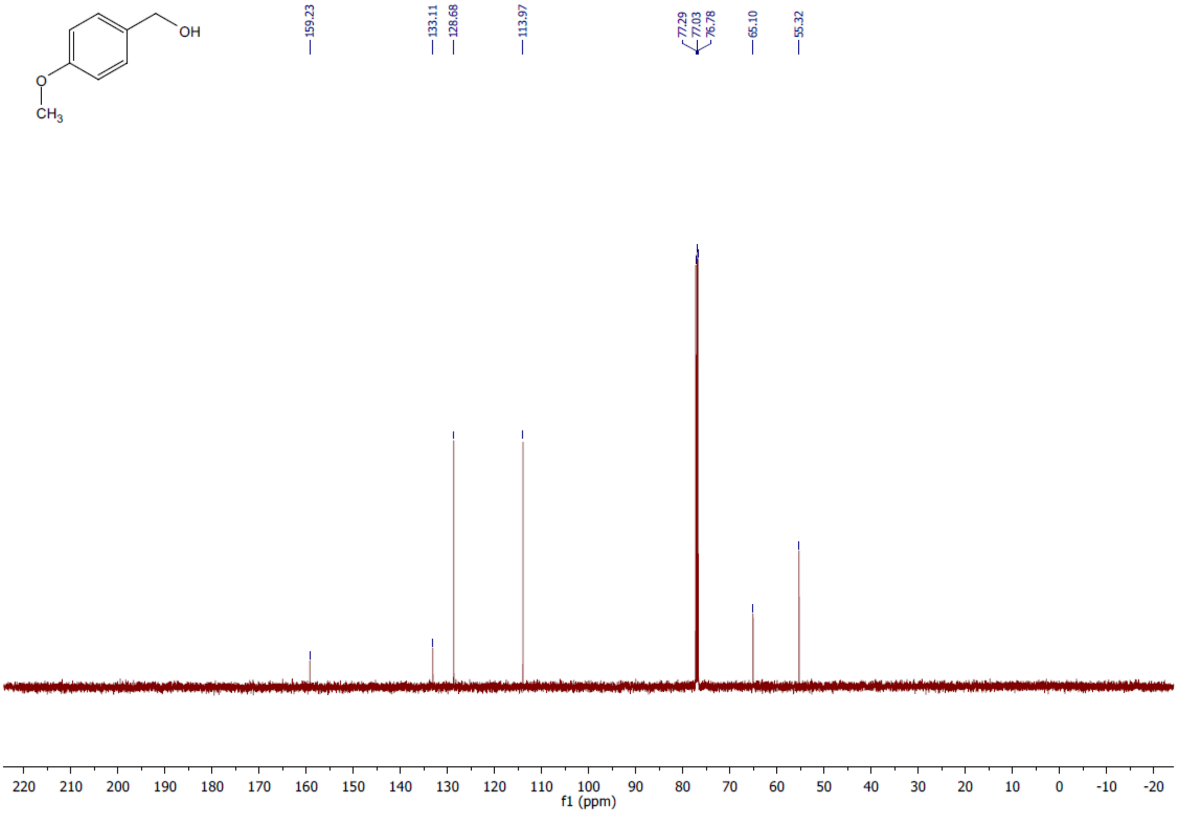

Figure S2. ^{13}C-NMR spectrum of alcohol **4a**.

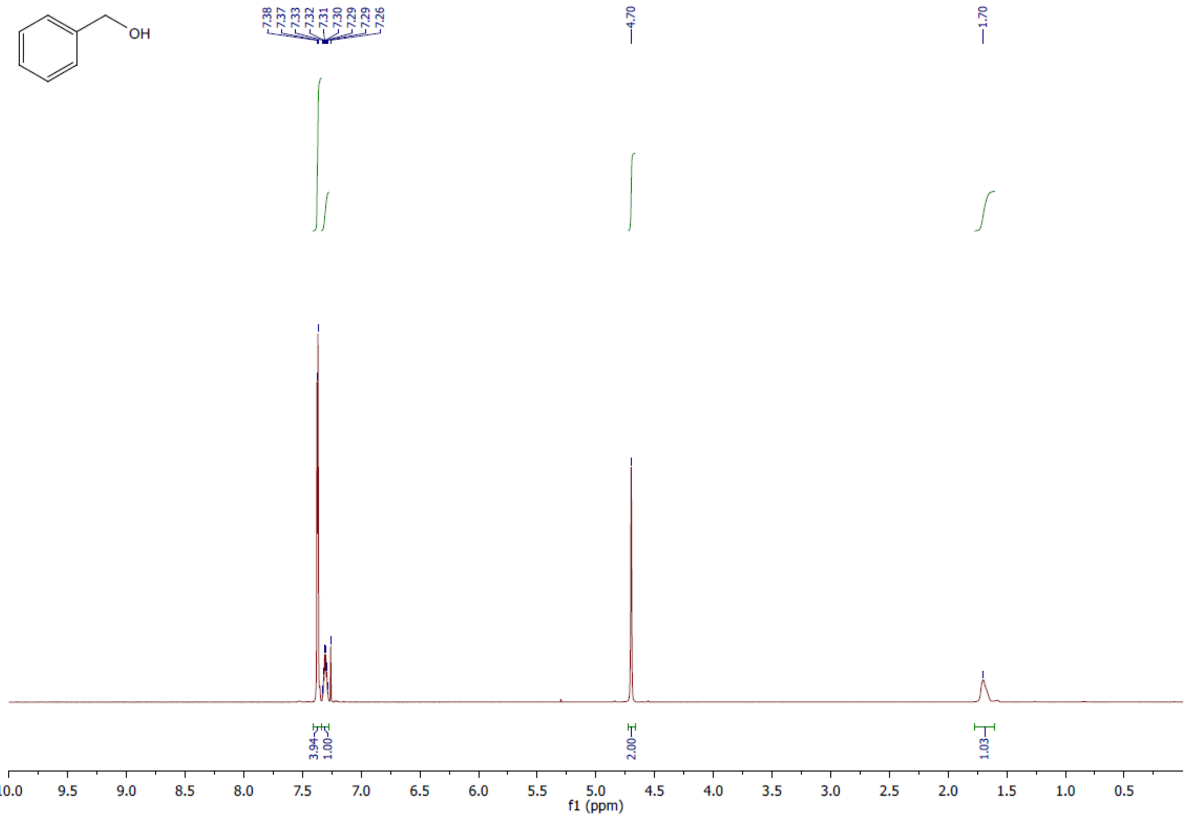

Figure S3. ^{1}H-NMR spectrum of alcohol 4**b**.

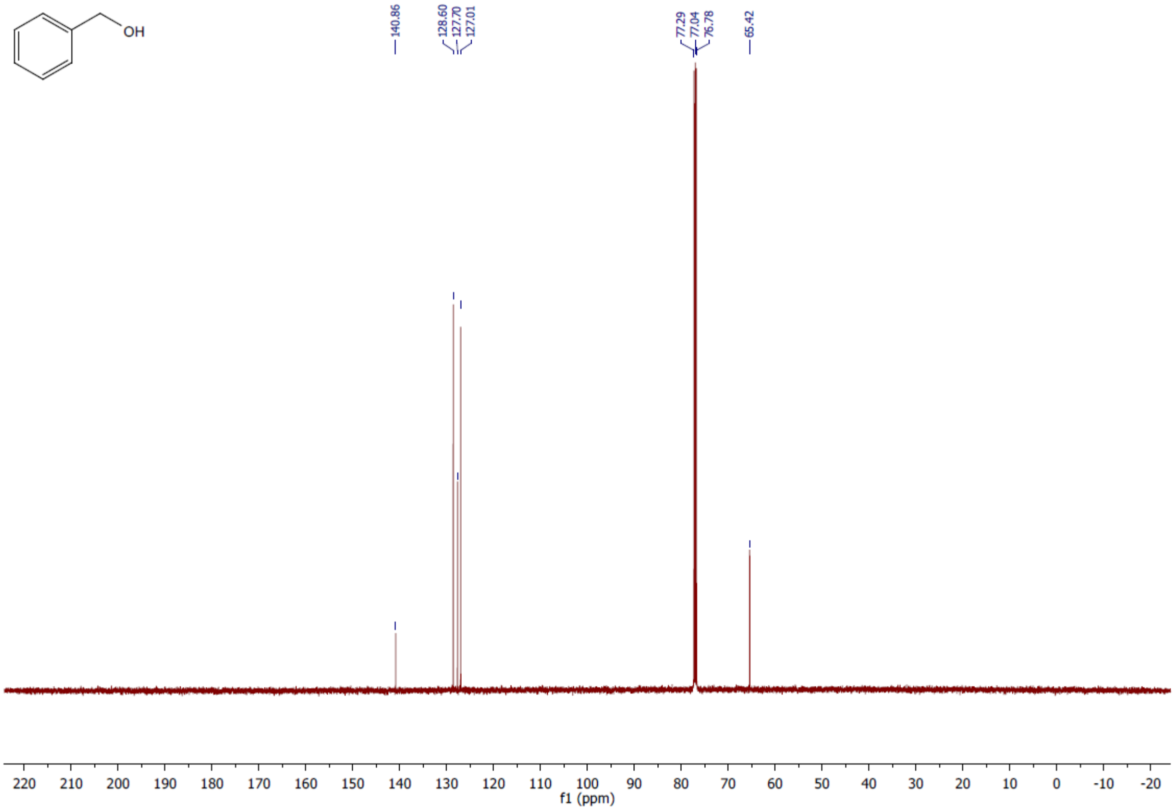

Figure S4. ^{13}C-NMR spectrum of alcohol **4b**.

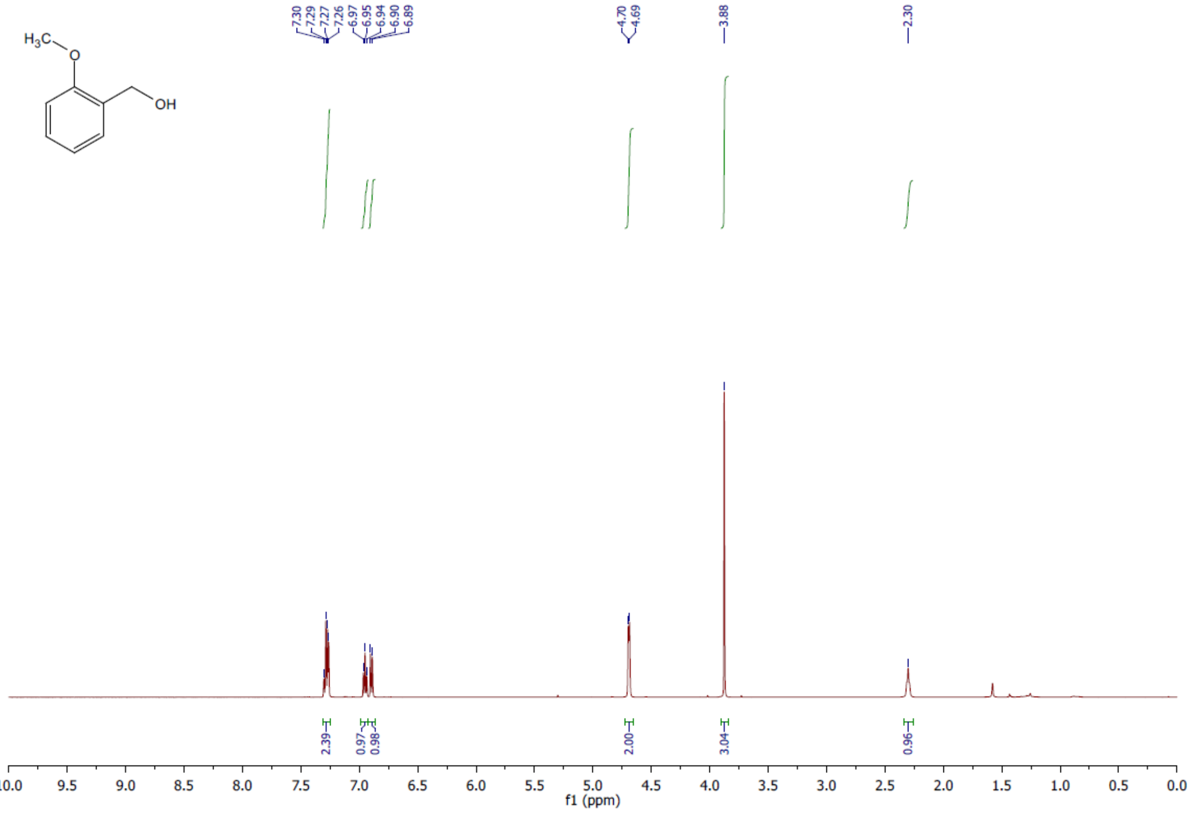

Figure S5. ^{1}H-NMR spectrum of alcohol **4c**.

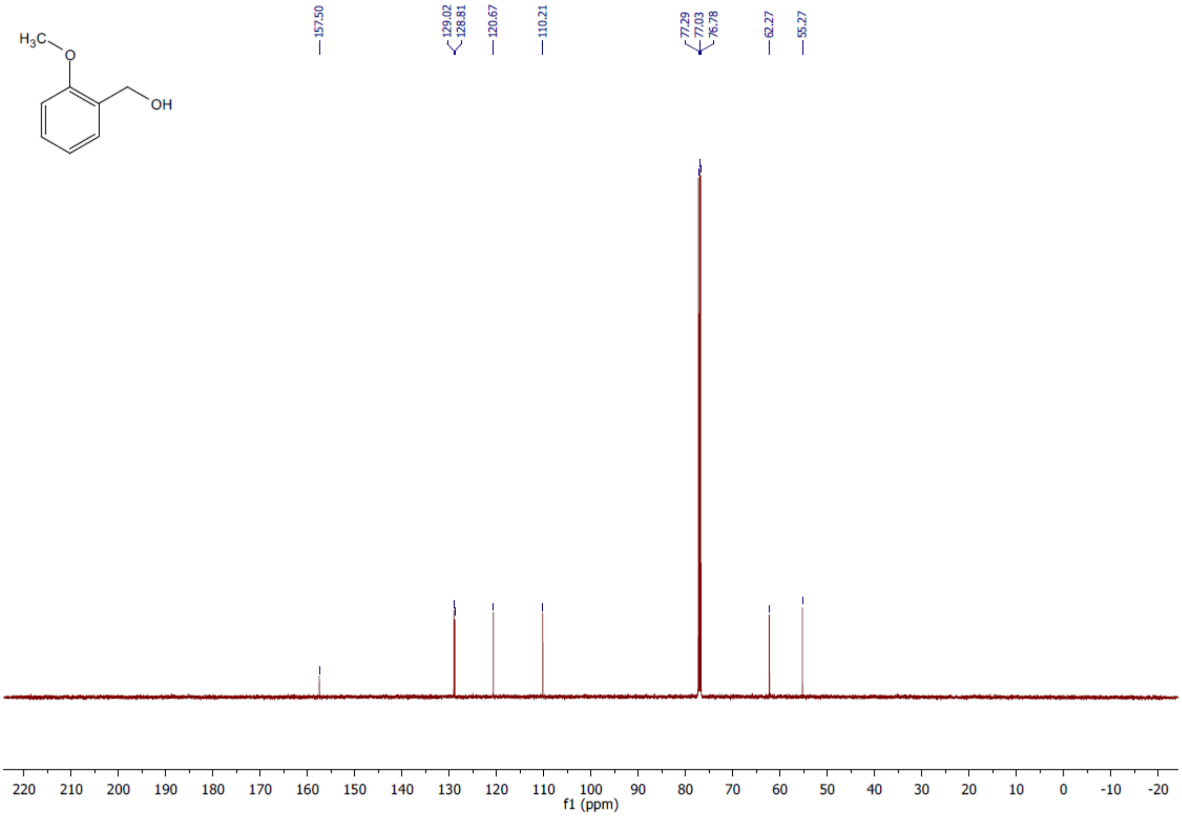

Figure S6. ^{13}C-NMR spectrum of alcohol **4c**.

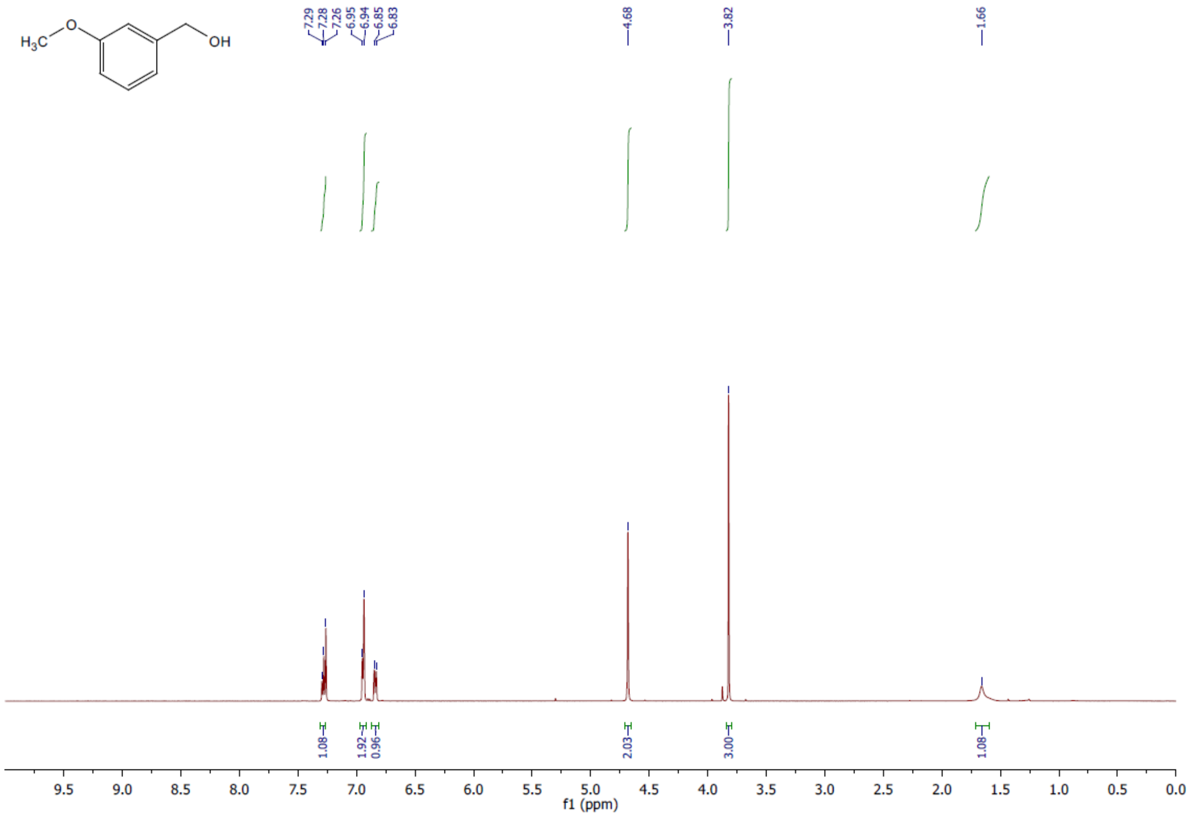

Figure S7. ^{1}H-NMR spectrum of alcohol **4d**.

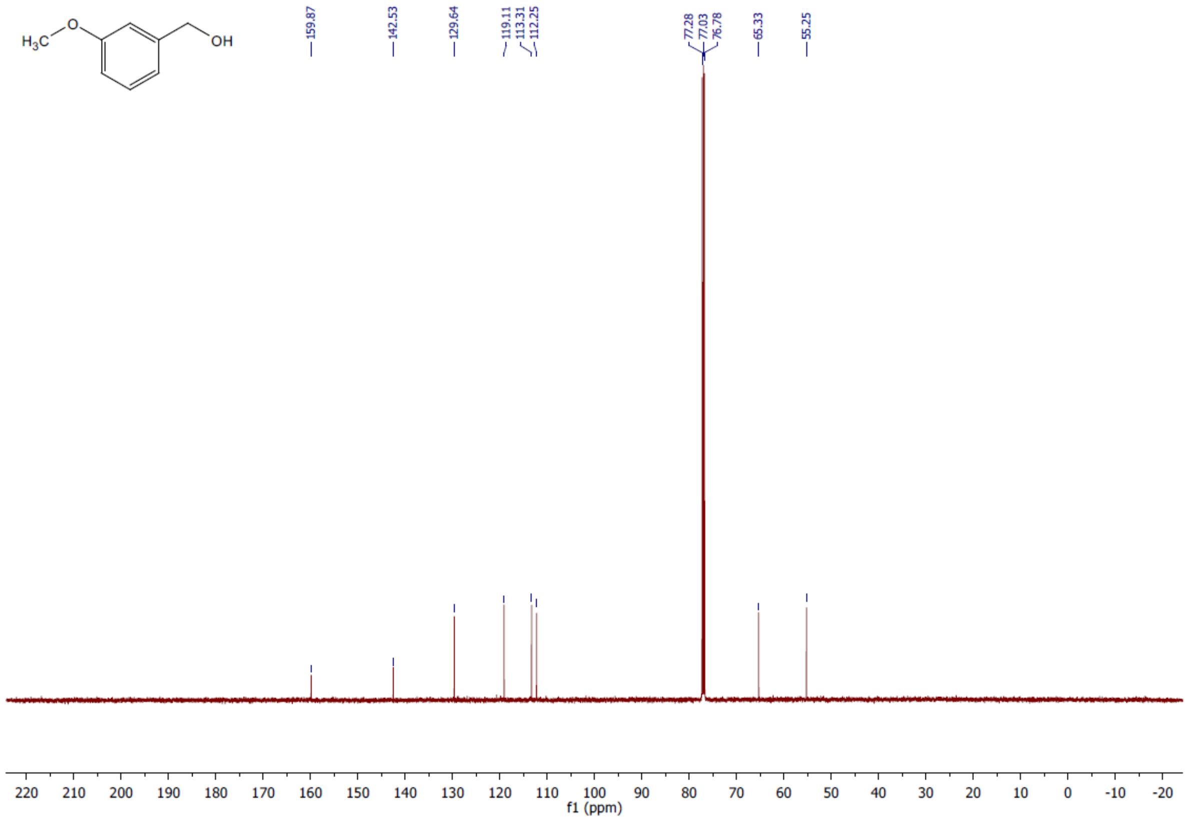

Figure S8. ^{13}C-NMR spectrum of alcohol **4d**.

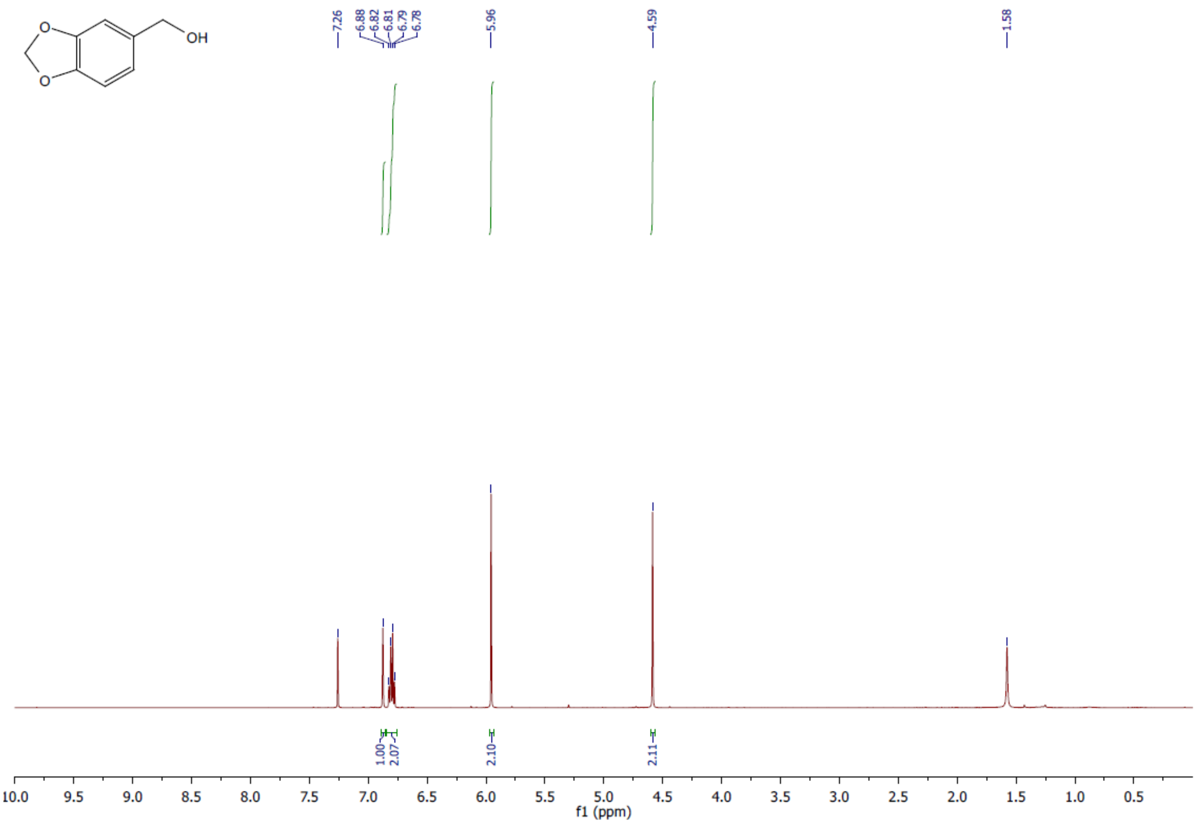

Figure S9. ^{1}H-NMR spectrum of alcohol **4e**.

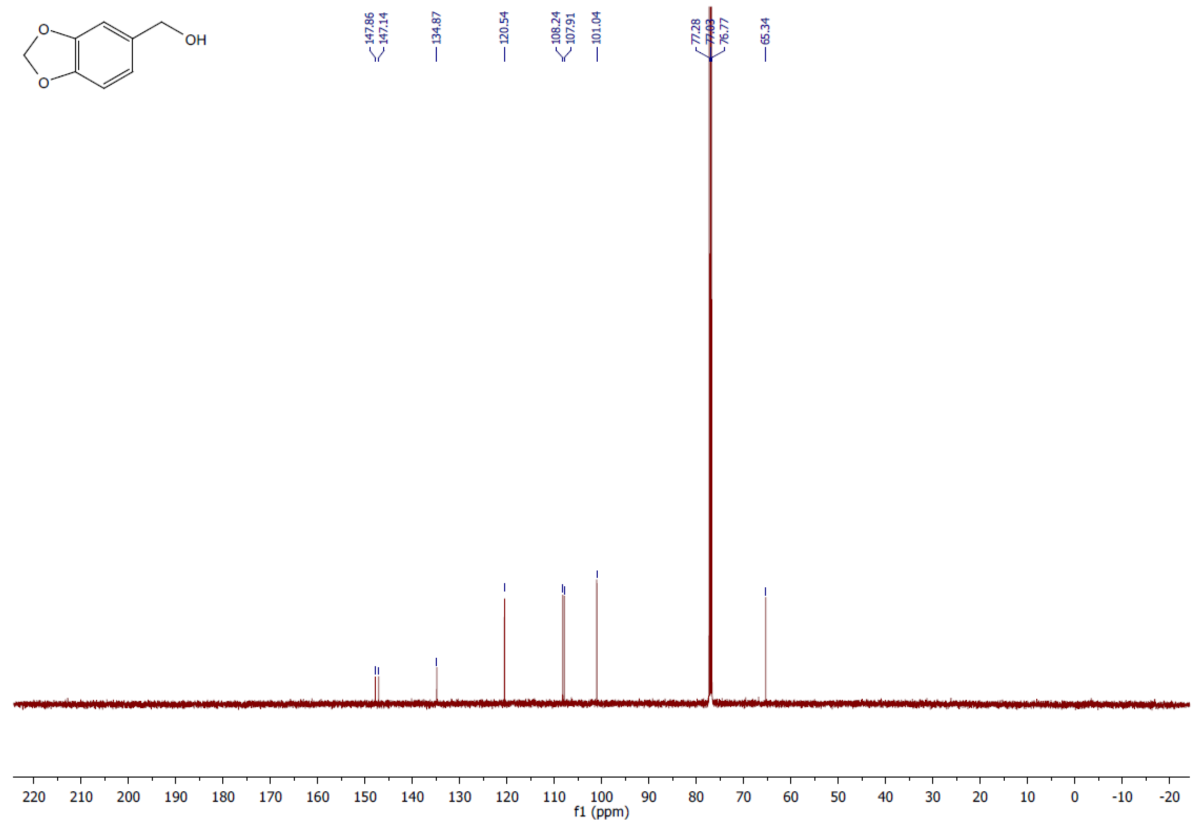

Figure S10. ^{13}C-NMR spectrum of alcohol **4e**.

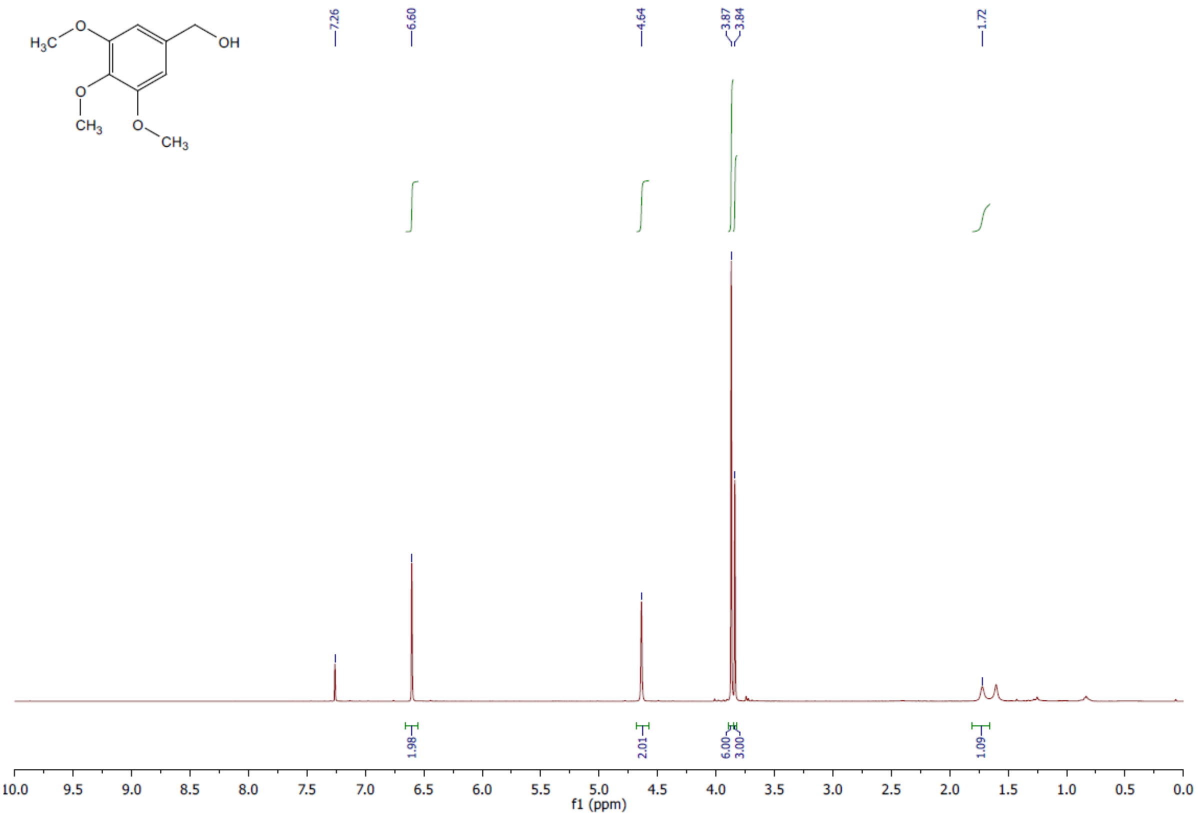

Figure S11. ^{1}H-NMR spectrum of alcohol **4f**.

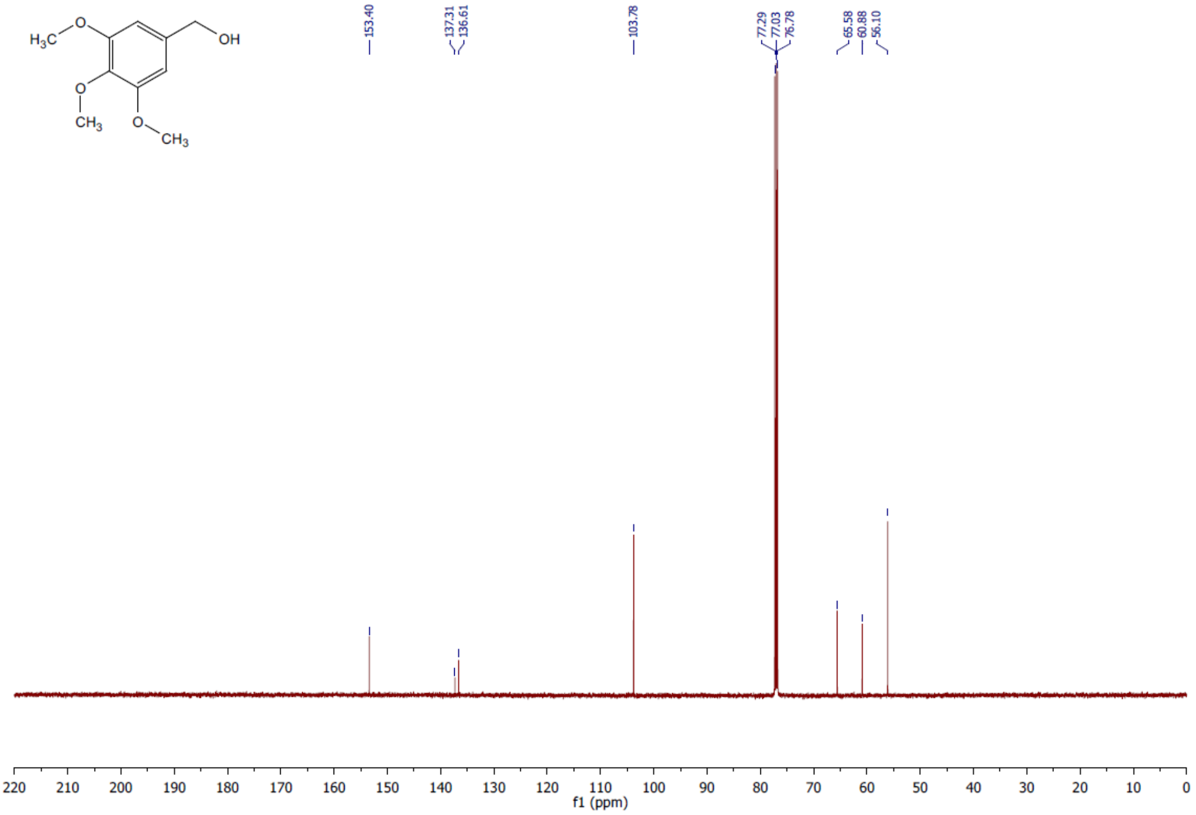

Figure S12. ^{13}C-NMR spectrum of alcohol **4f**.

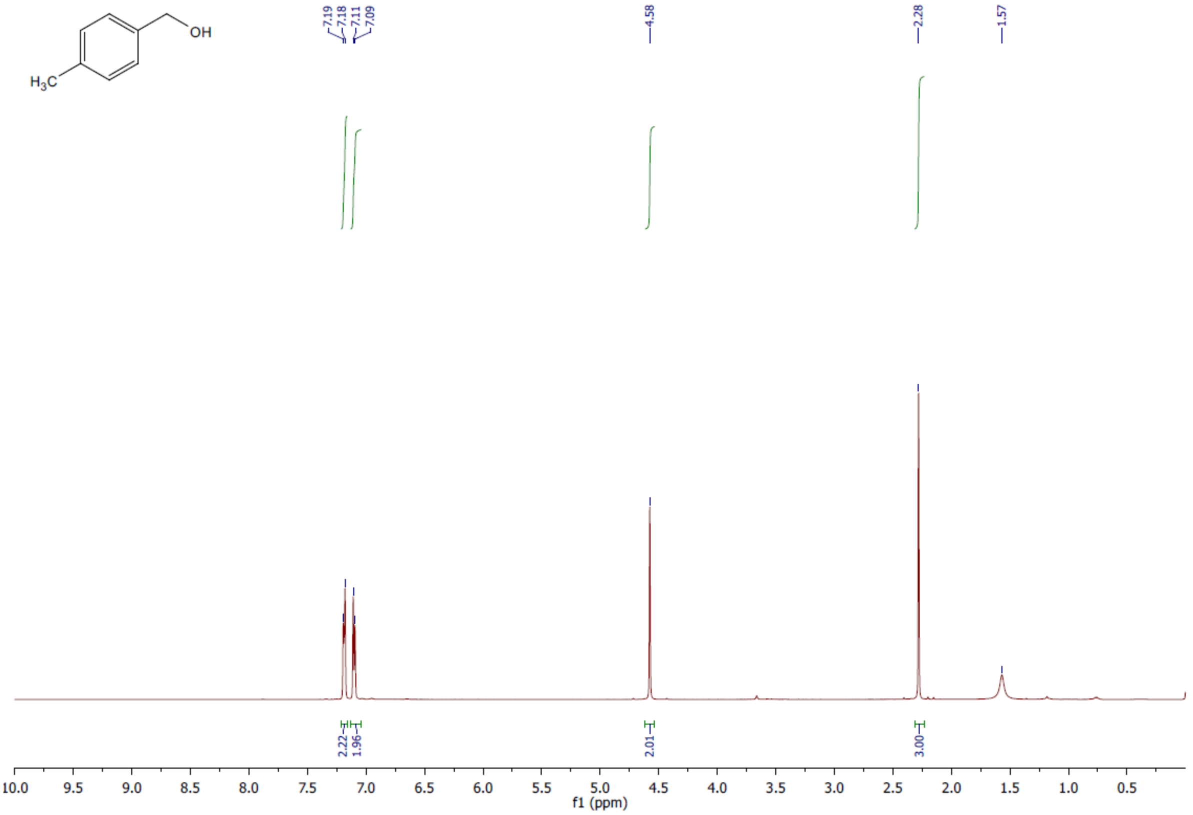

Figure S13. ^{1}H-NMR spectrum of alcohol **4g**.

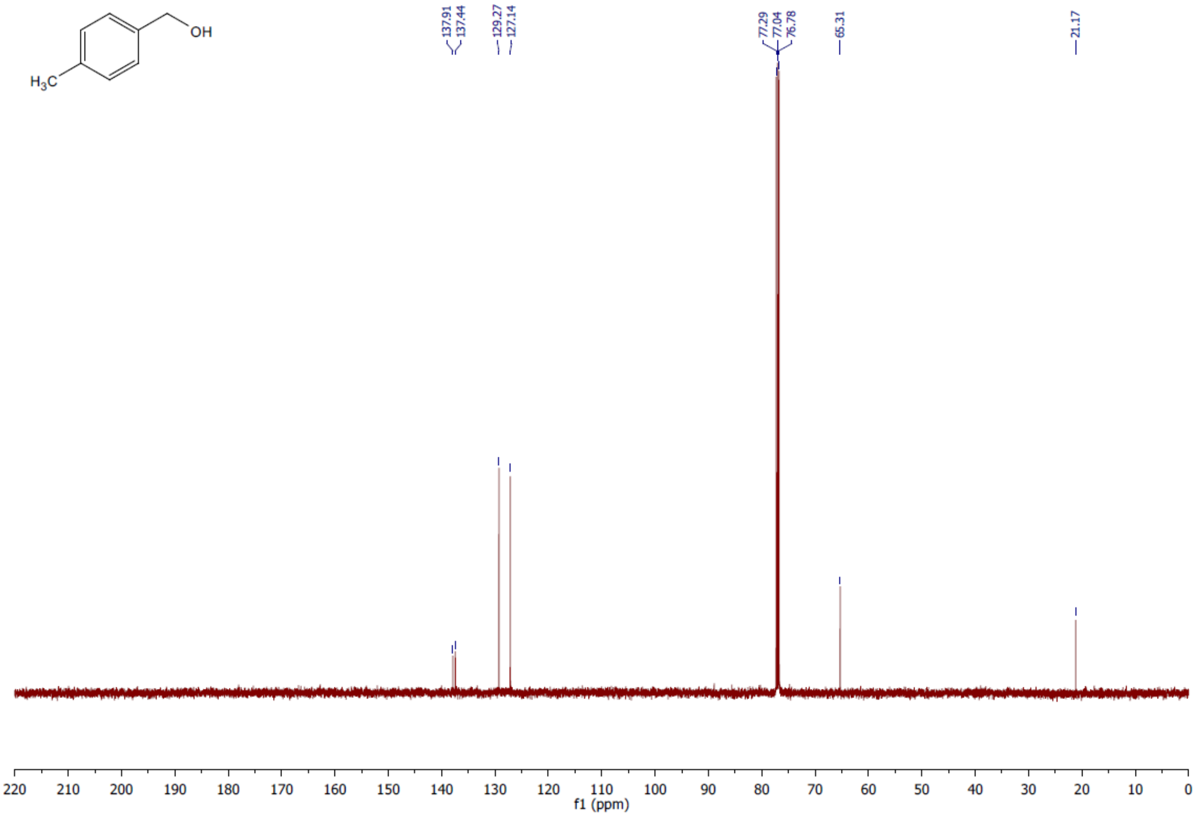

Figure S14. ^{13}C-NMR spectrum of alcohol **4g**.

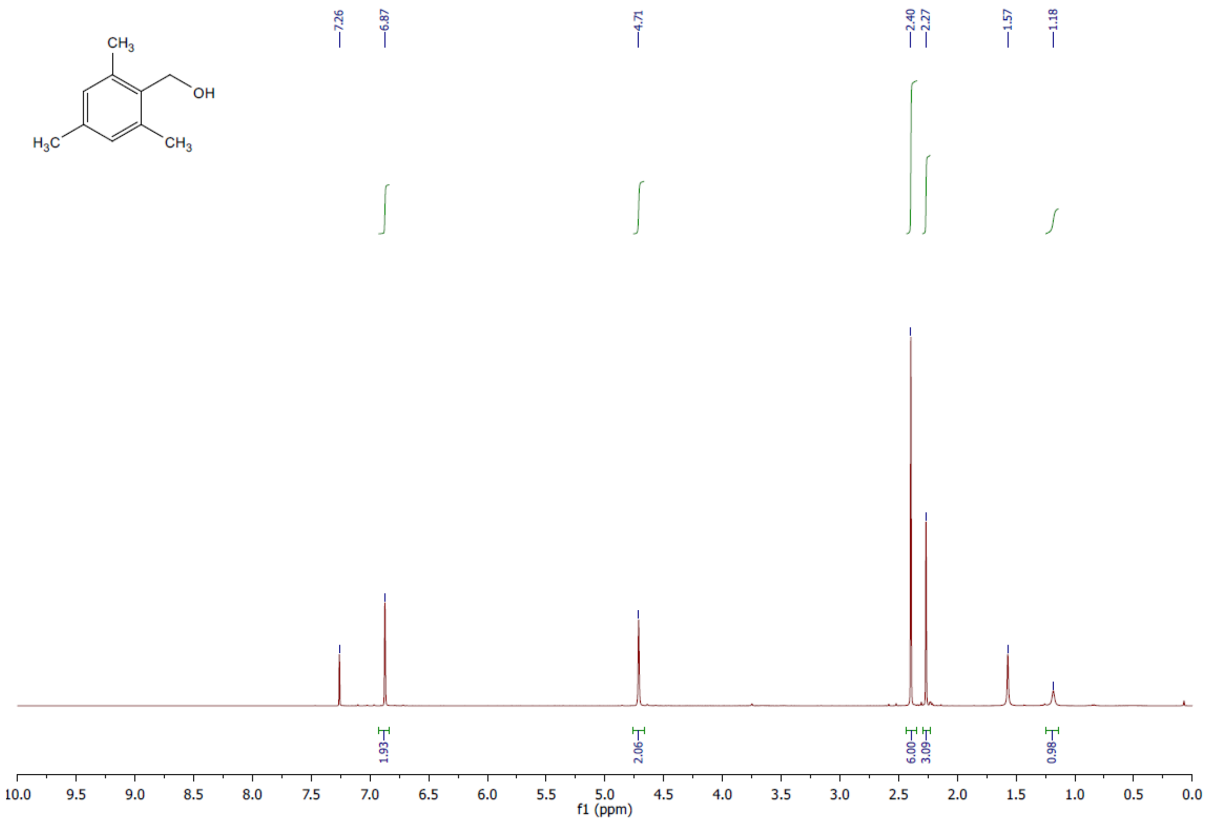

Figure S15. ^{1}H-NMR spectrum of alcohol **4h**.

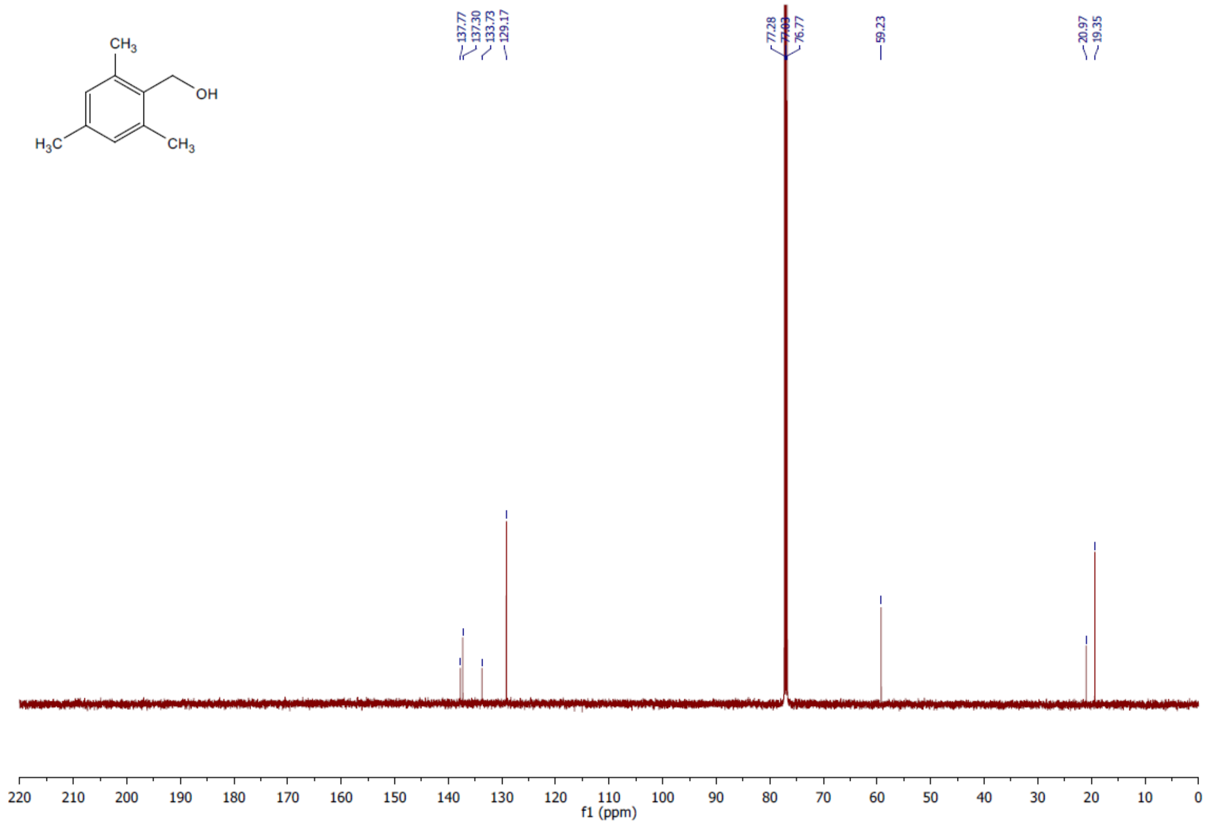

Figure S16. ^{13}C-NMR spectrum of alcohol **4h**.

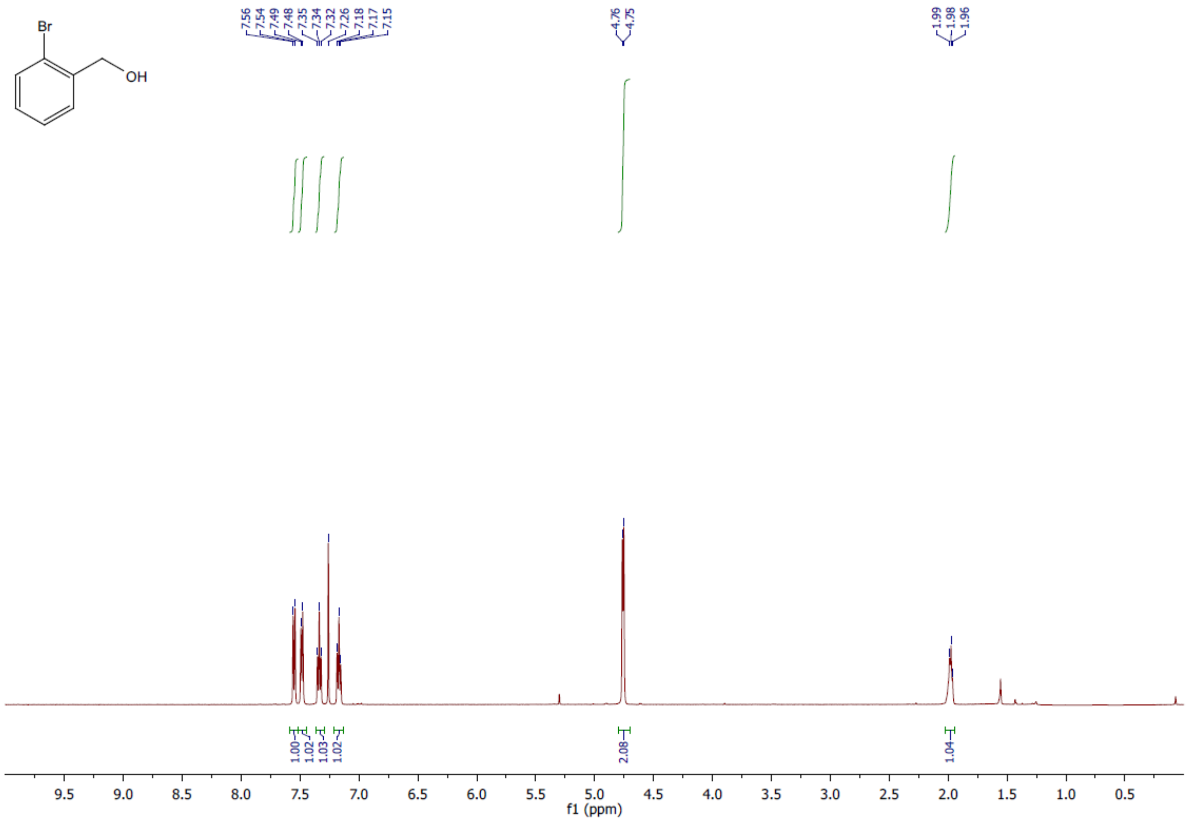

Figure S17. ^{1}H-NMR spectrum of alcohol **4i**.

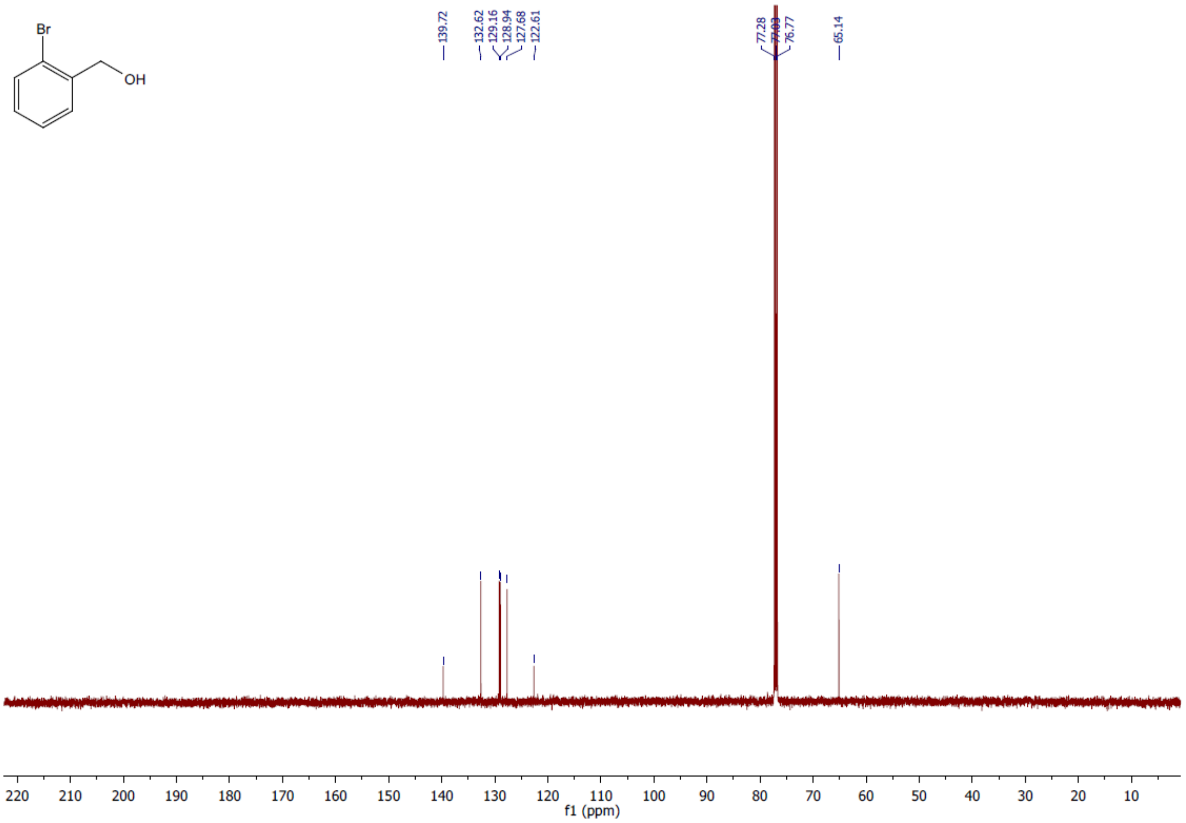

Figure S18. ^{13}C-NMR spectrum of alcohol **4i**.

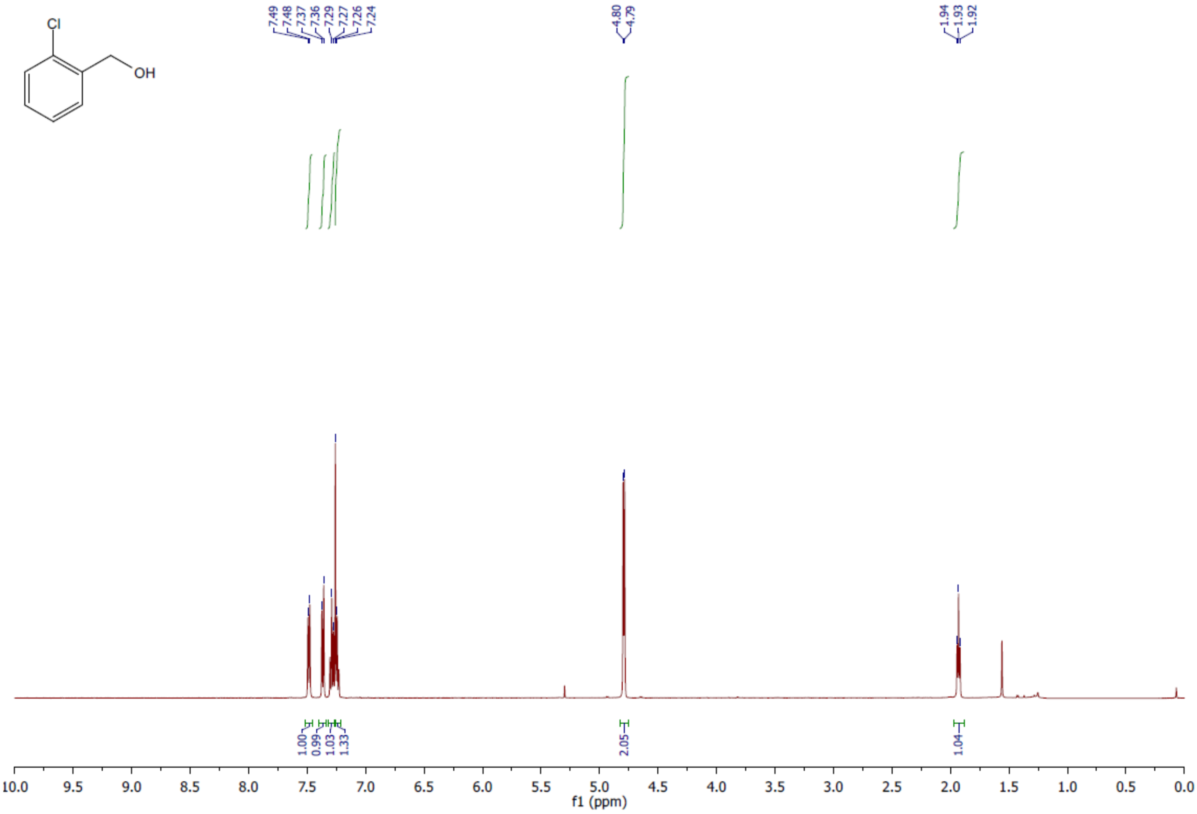

Figure S19. ^{1}H-NMR spectrum of alcohol **4j**.

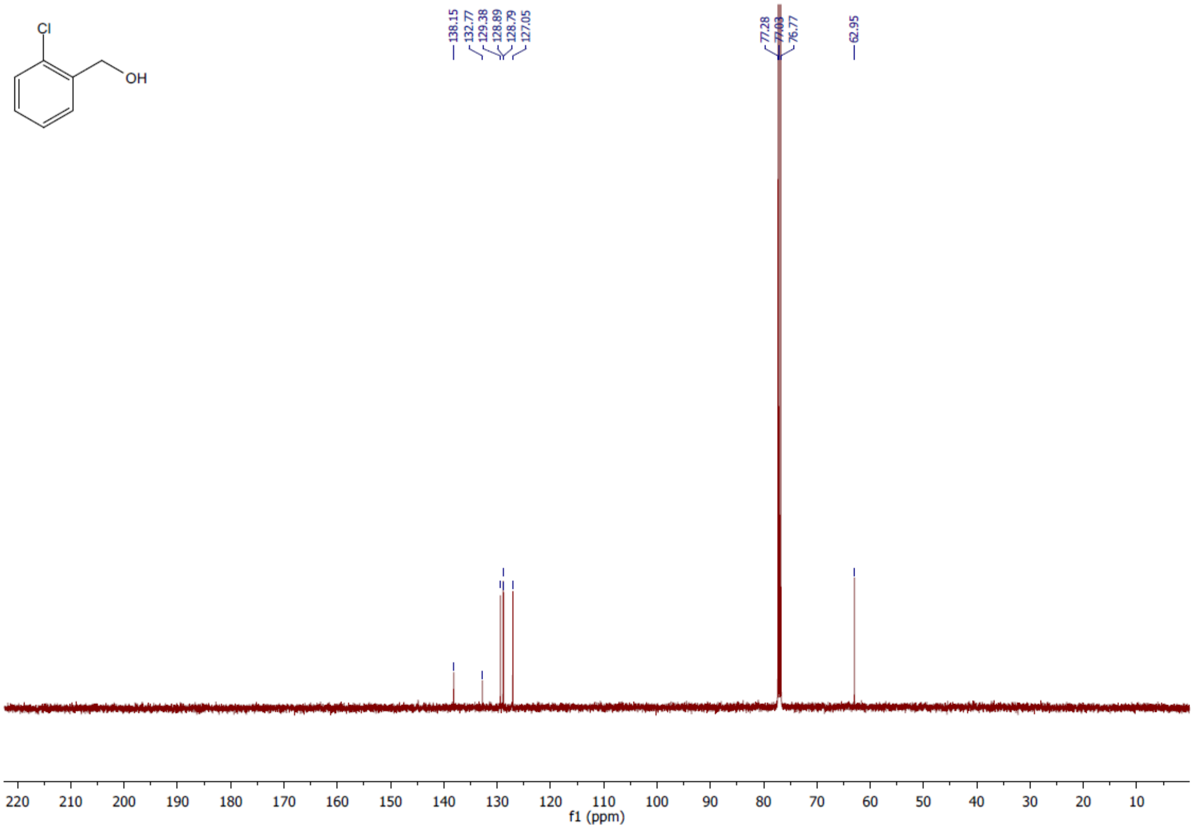

Figure S20. ^{13}C-NMR spectrum of alcohol **4j**.

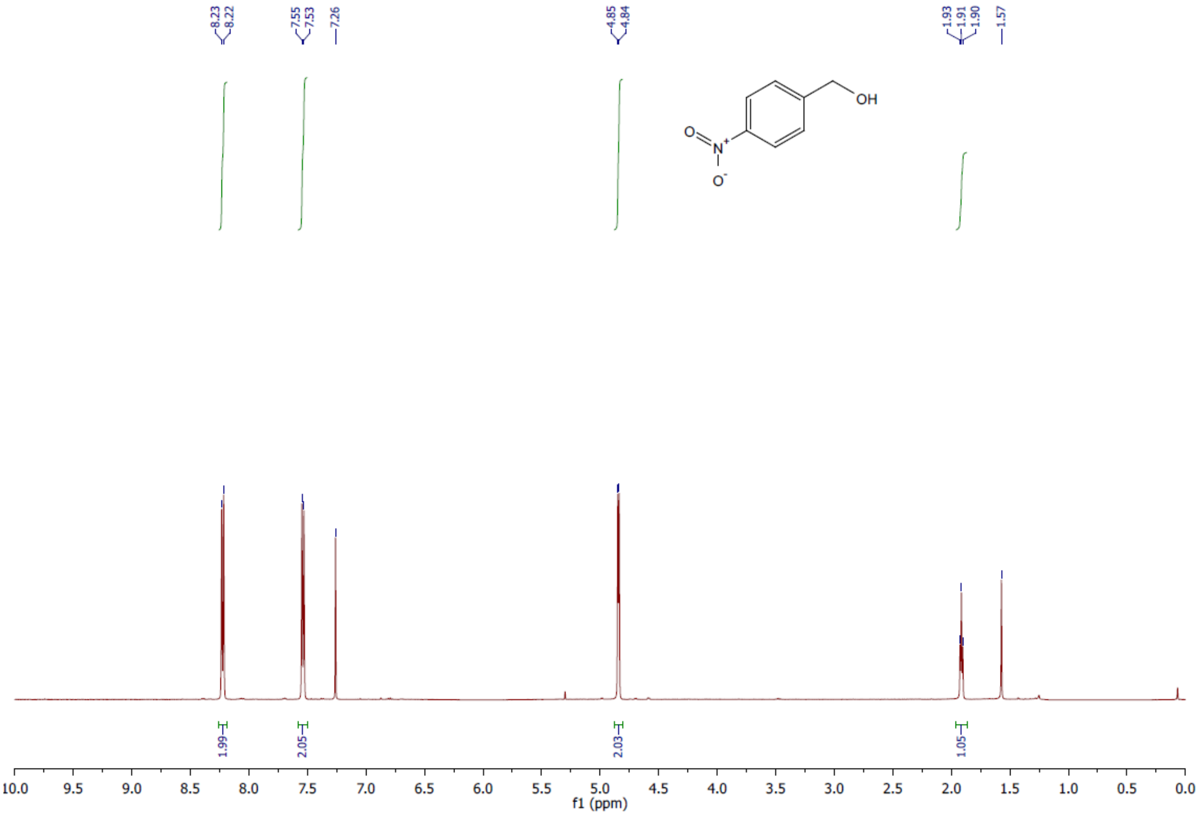

Figure S21. ^{1}H-NMR spectrum of alcohol **4k**.

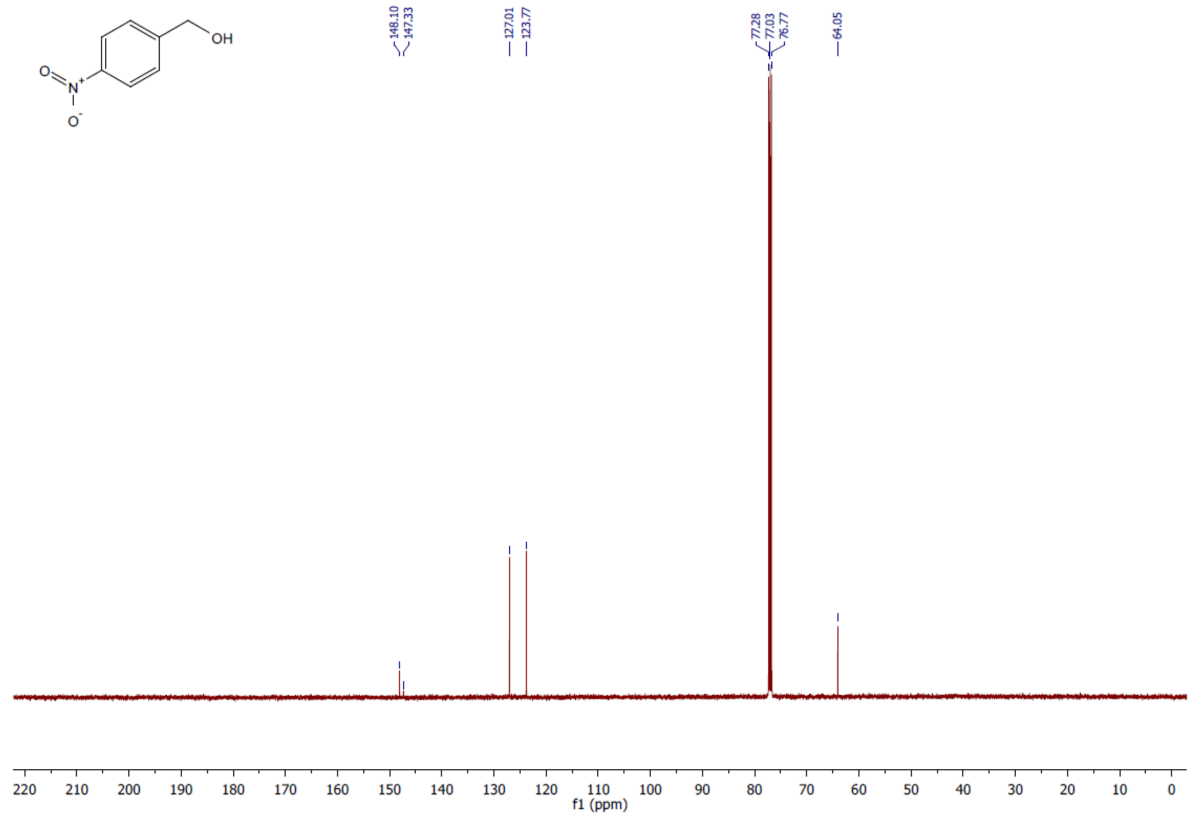

Figure S22. ^{13}C-NMR spectrum of alcohol **4k**.

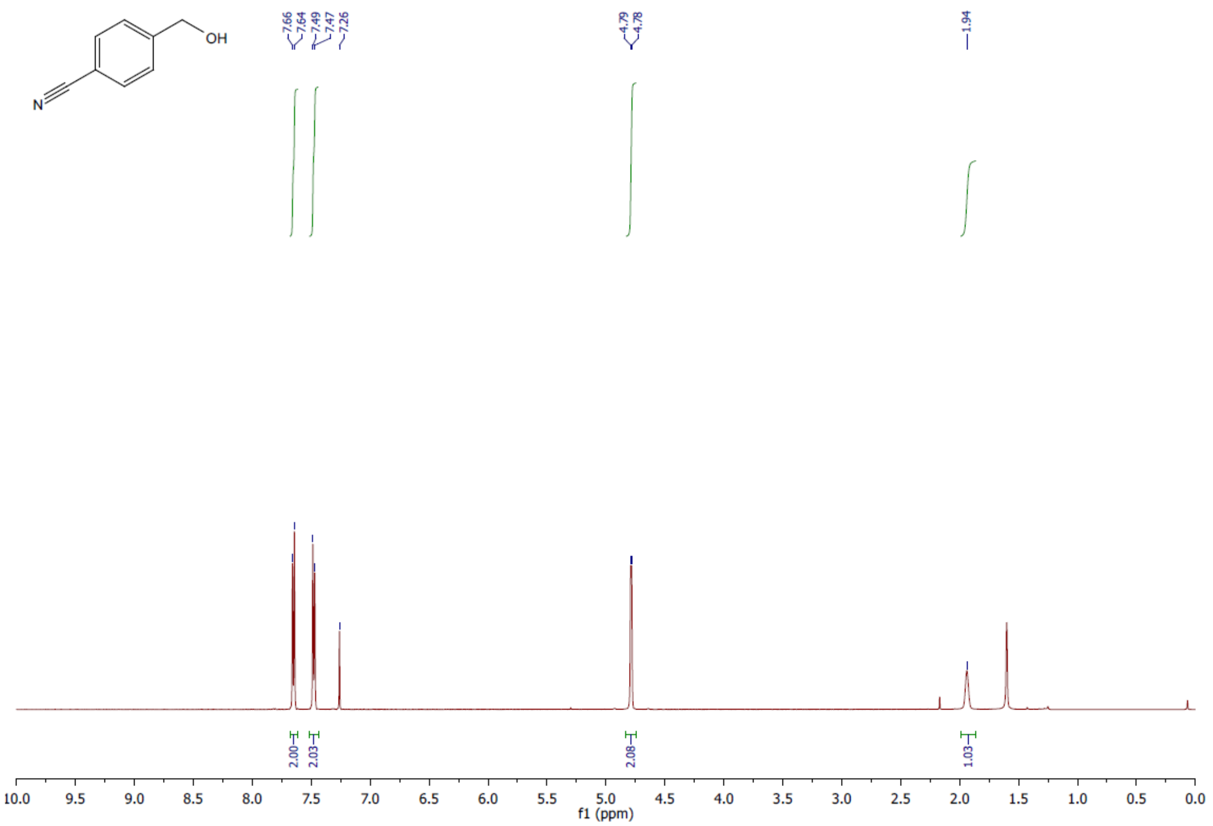

Figure S23. ^{1}H-NMR spectrum of alcohol **4l**.

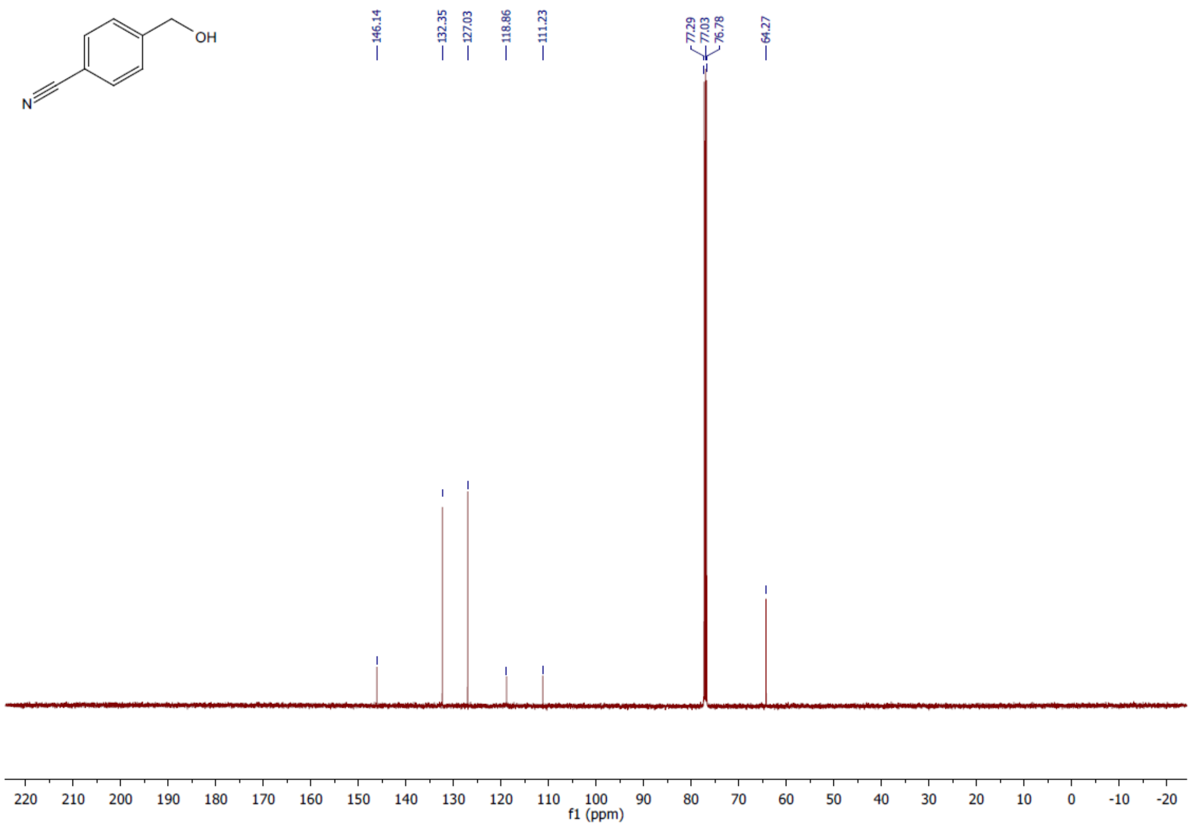

Figure S24. ^{13}C-NMR spectrum of alcohol **4l**.

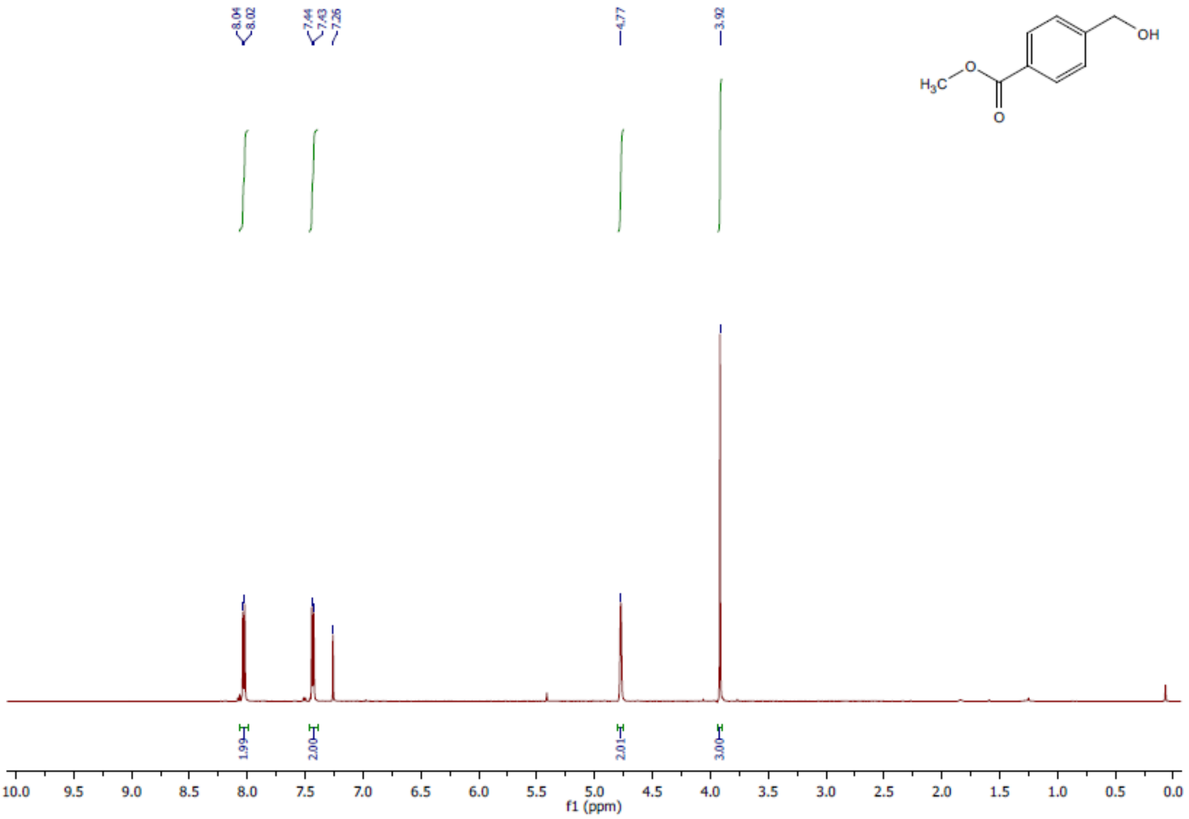

Figure S25. ^{1}H-NMR spectrum of alcohol **4m**.

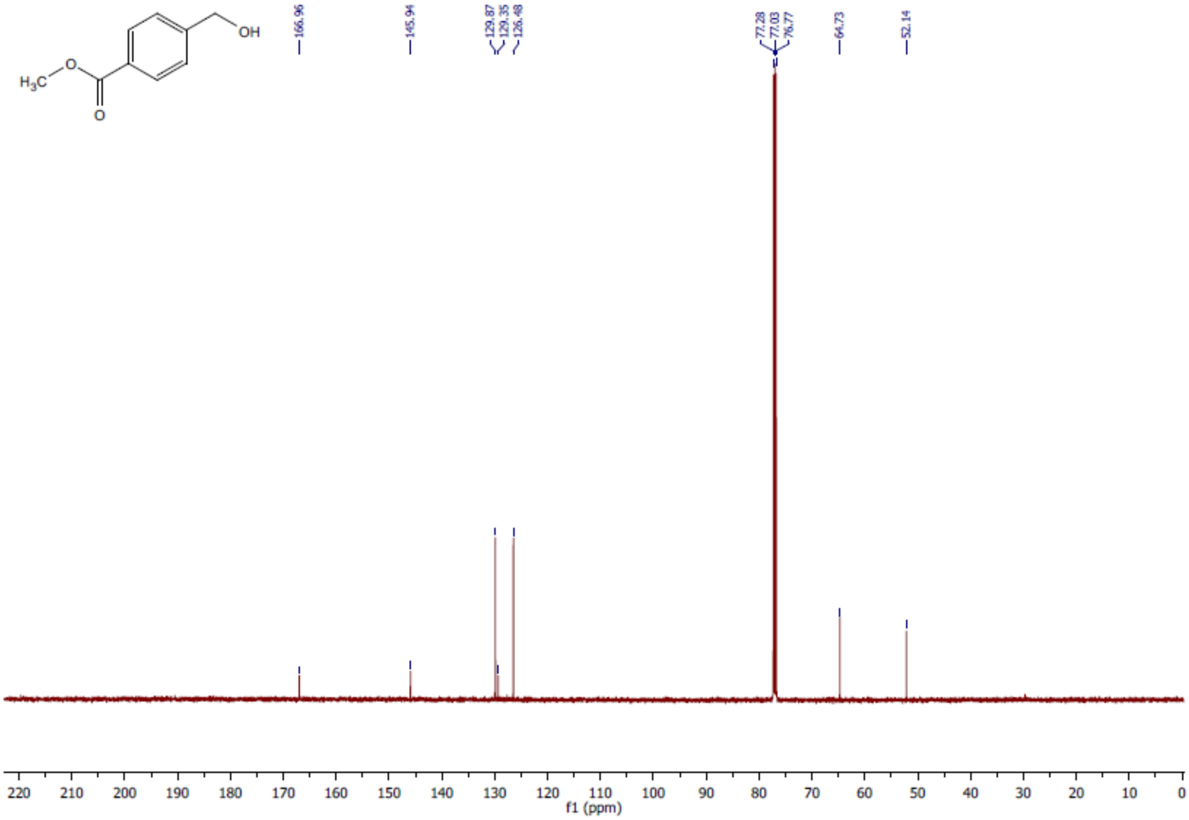

Figure S26. ^{13}C-NMR spectrum of alcohol **4m**.

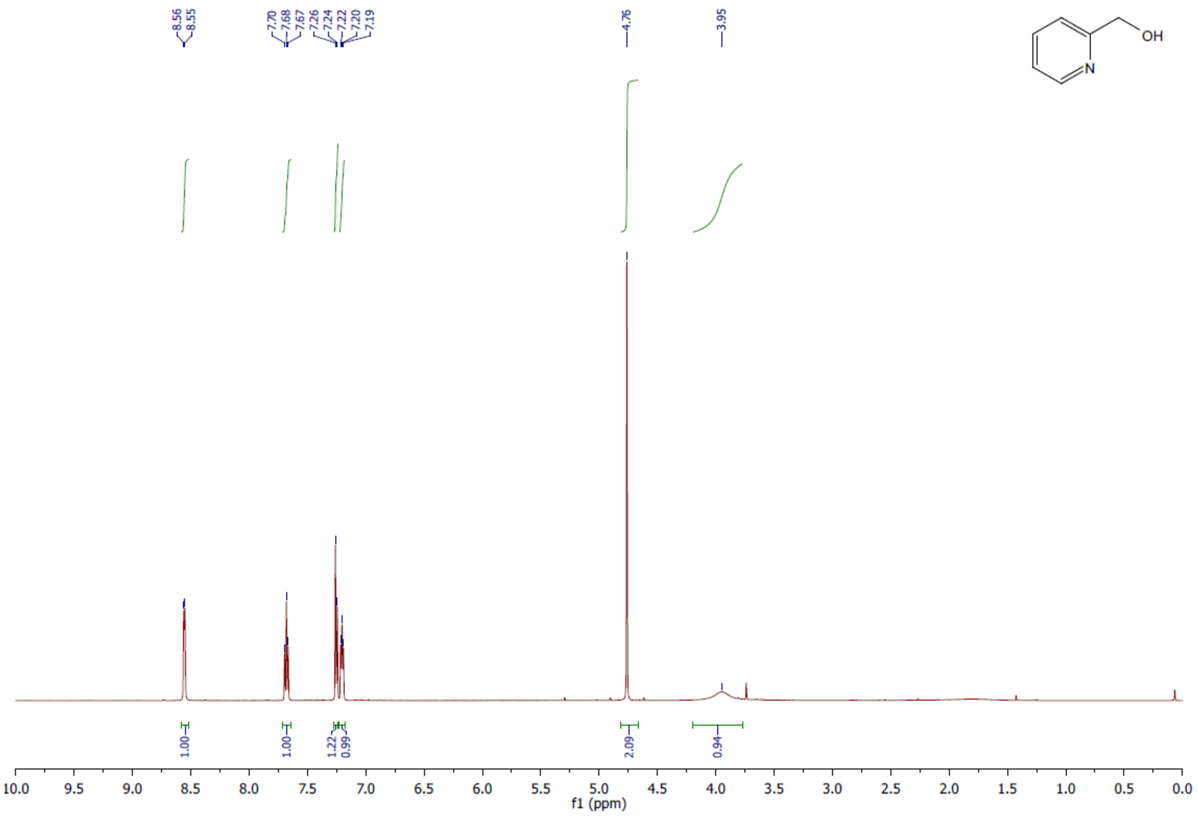

Figure S27. ^{1}H-NMR spectrum of alcohol **4n**.

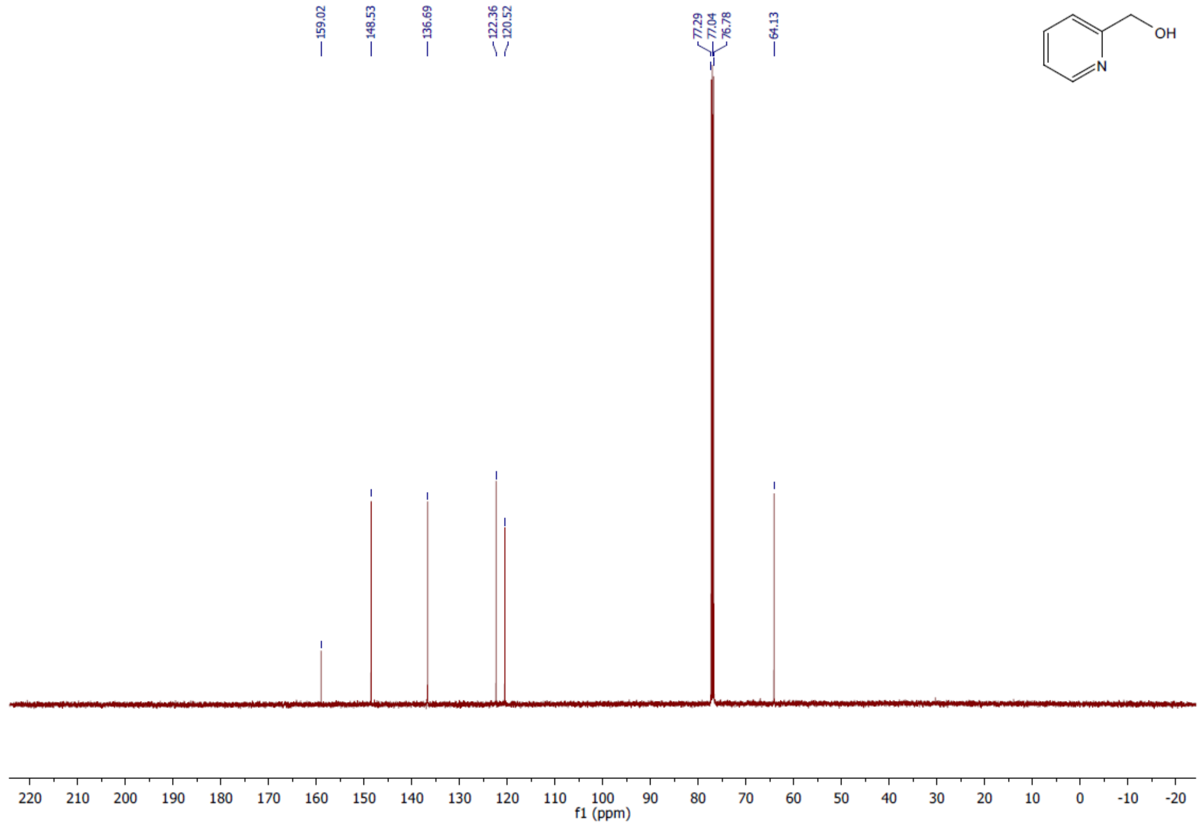

Figure S28. ^{13}C-NMR spectrum of alcohol **4n**.

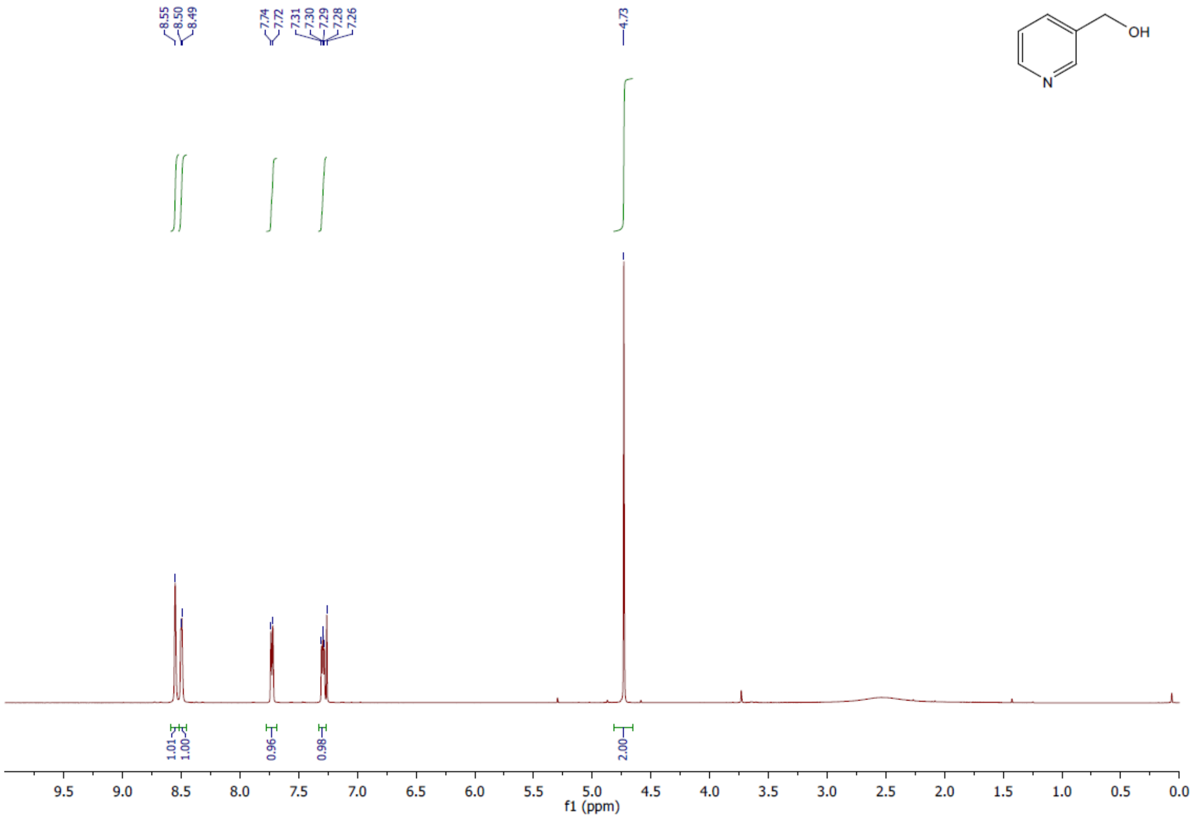

Figure S29. ^{1}H-NMR spectrum of alcohol **4o**.

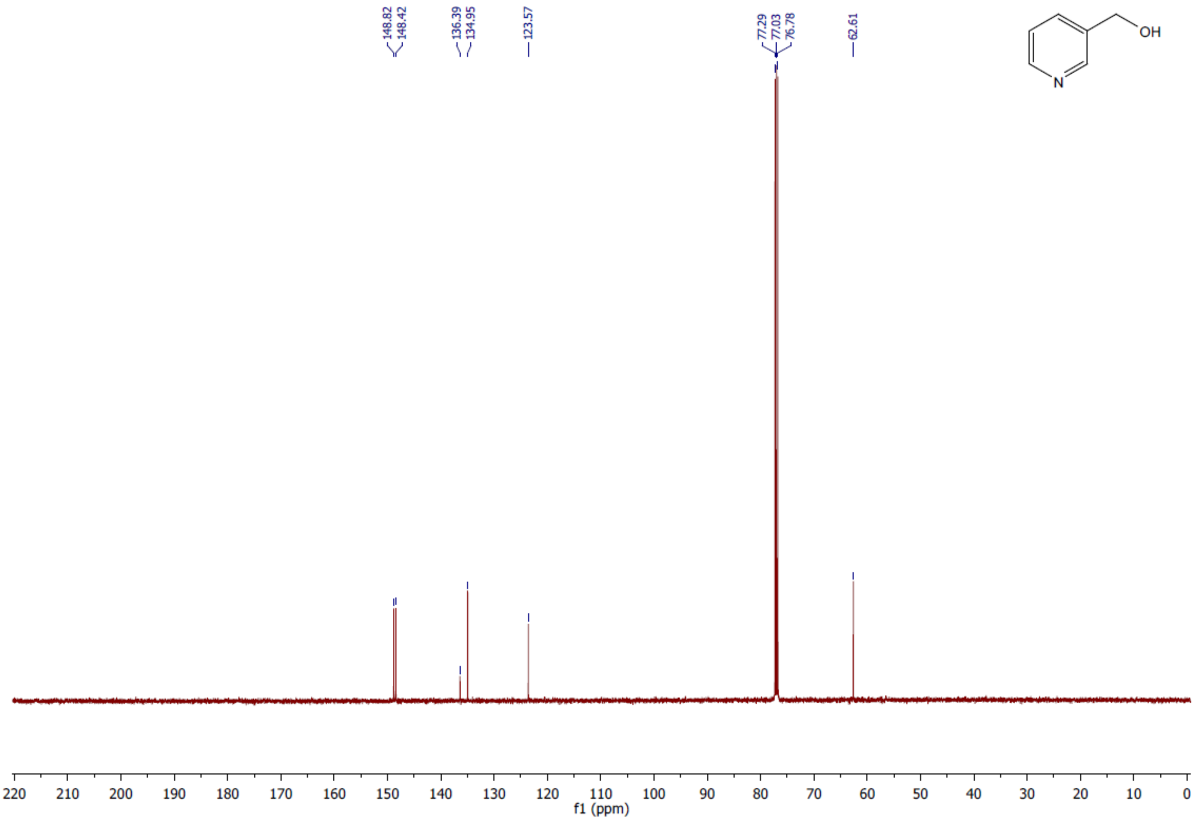

Figure S30. ^{13}C-NMR spectrum of alcohol **4o**.

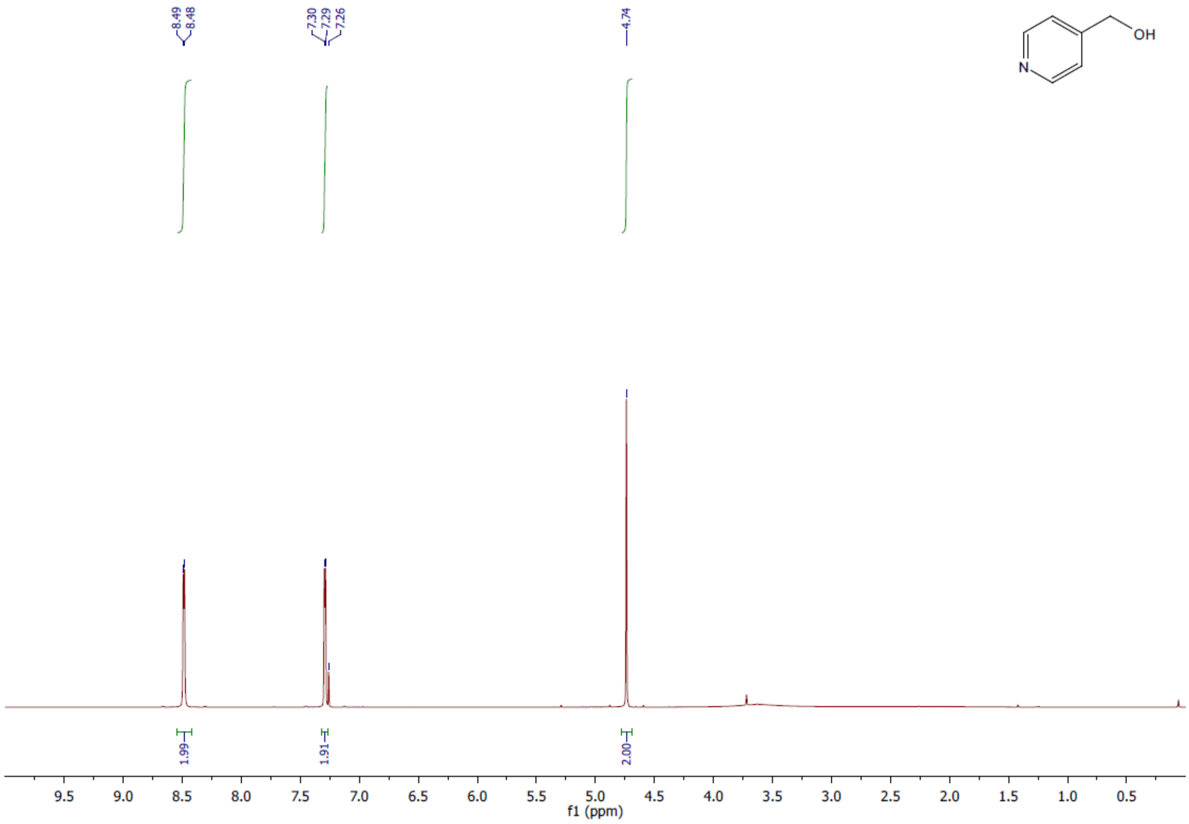

Figure S31. ^{1}H-NMR spectrum of alcohol **4p**.

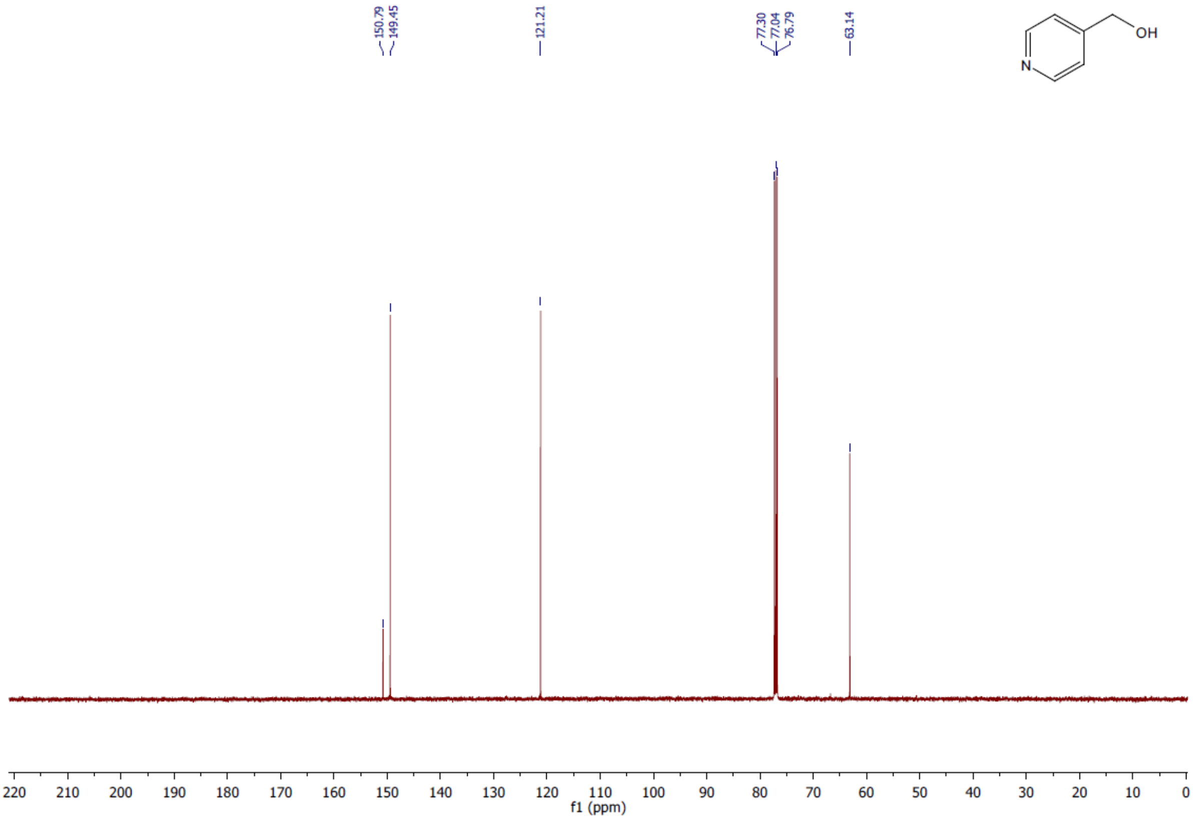

Figure S32. ^{13}C-NMR spectrum of alcohol **4p**.

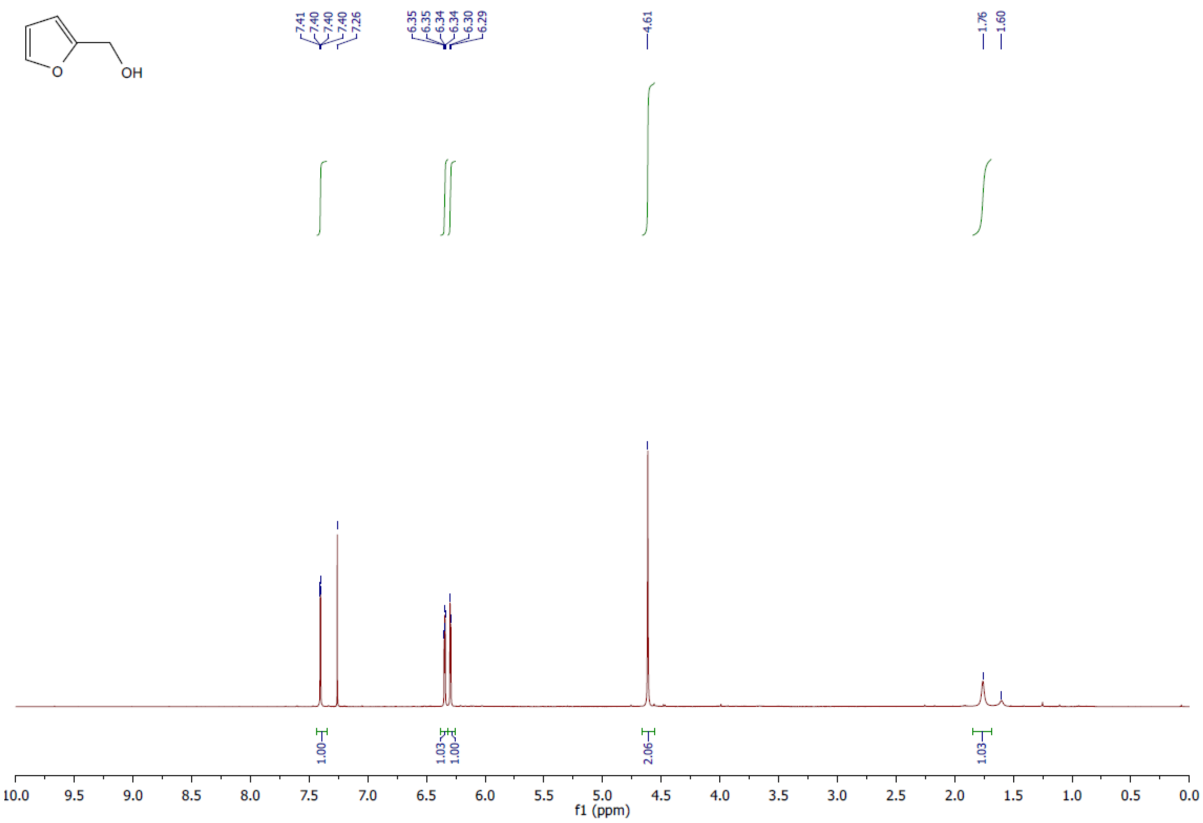

Figure S33. ^{1}H-NMR spectrum of alcohol **4q**.

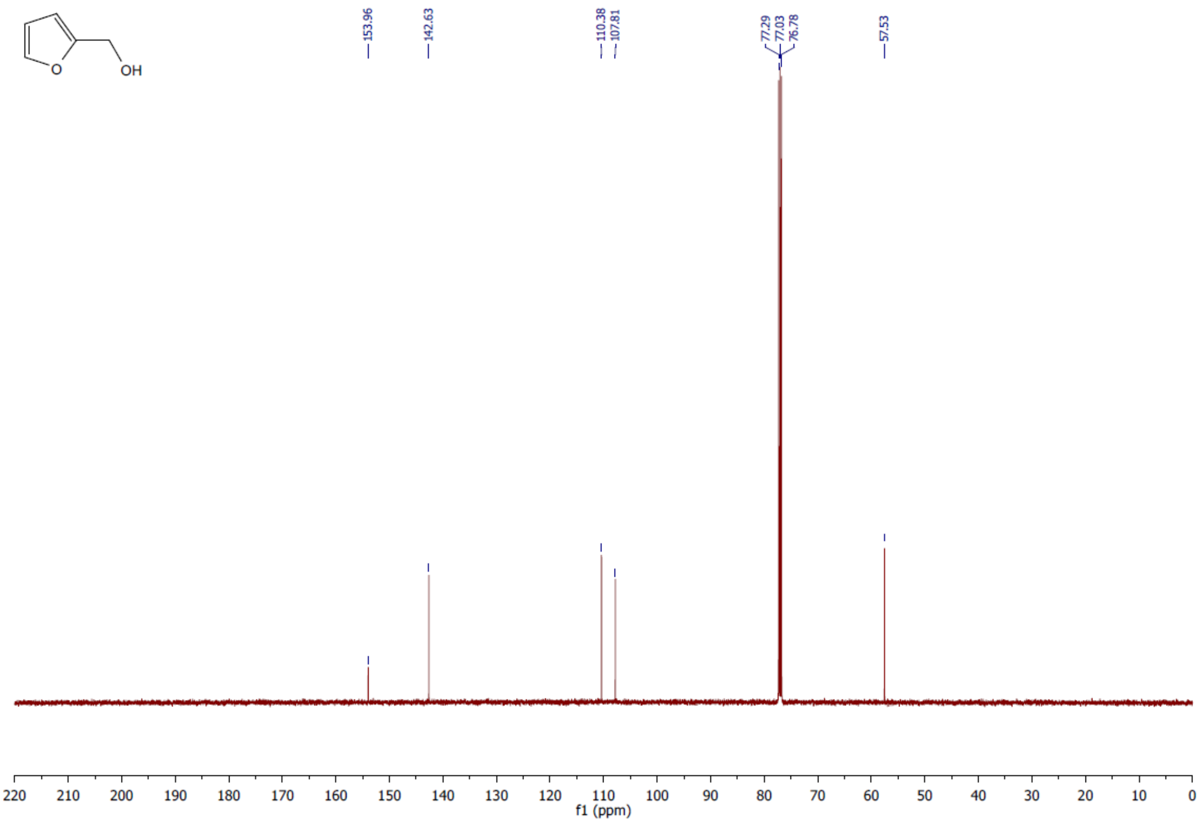

Figure S34. ^{13}C-NMR spectrum of alcohol **4q**.

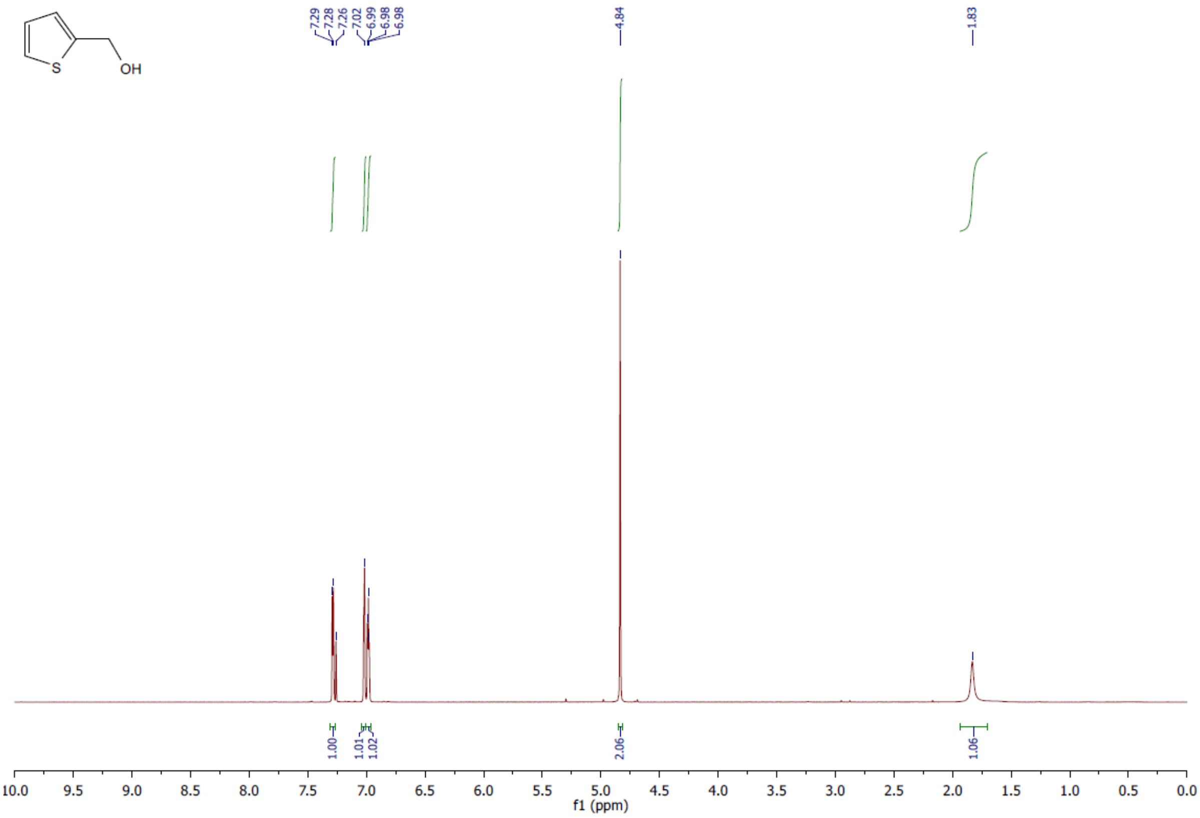

Figure S35. ^{1}H-NMR spectrum of alcohol **4r**.

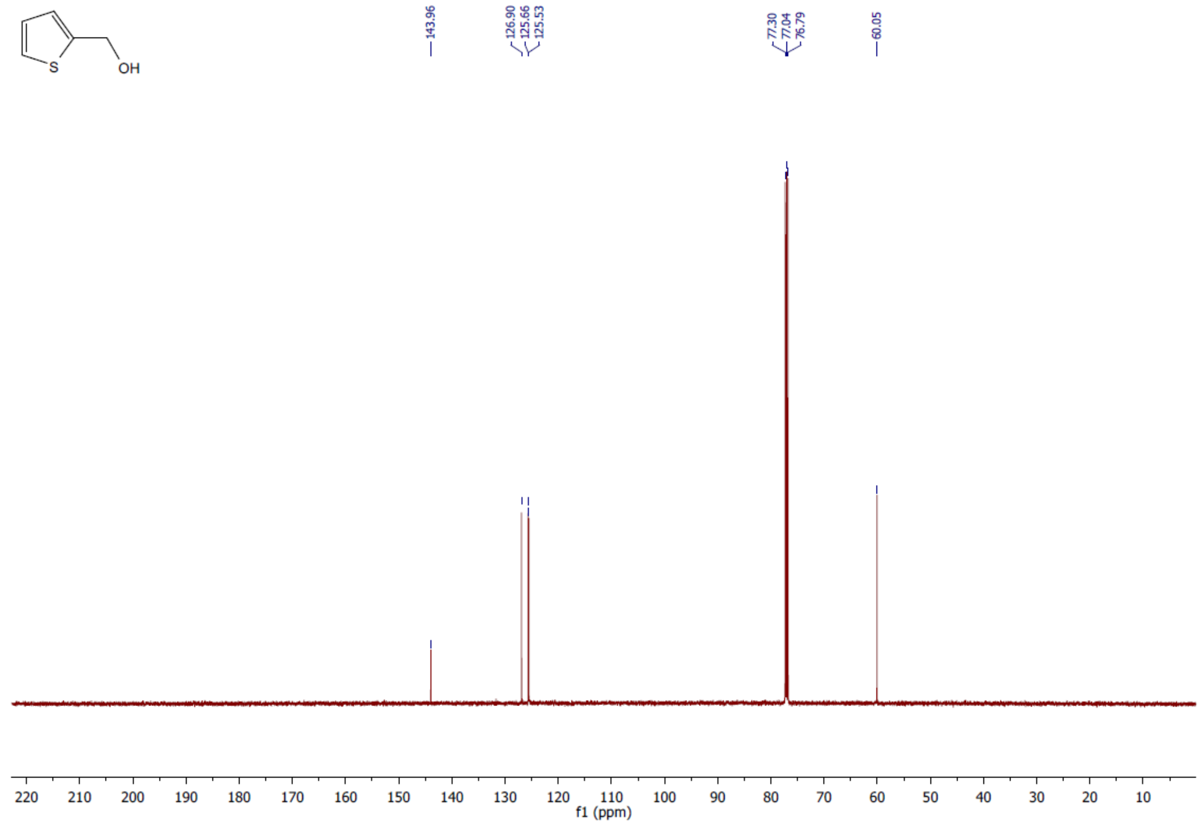

Figure S36. ^{13}C-NMR spectrum of alcohol **4r**.

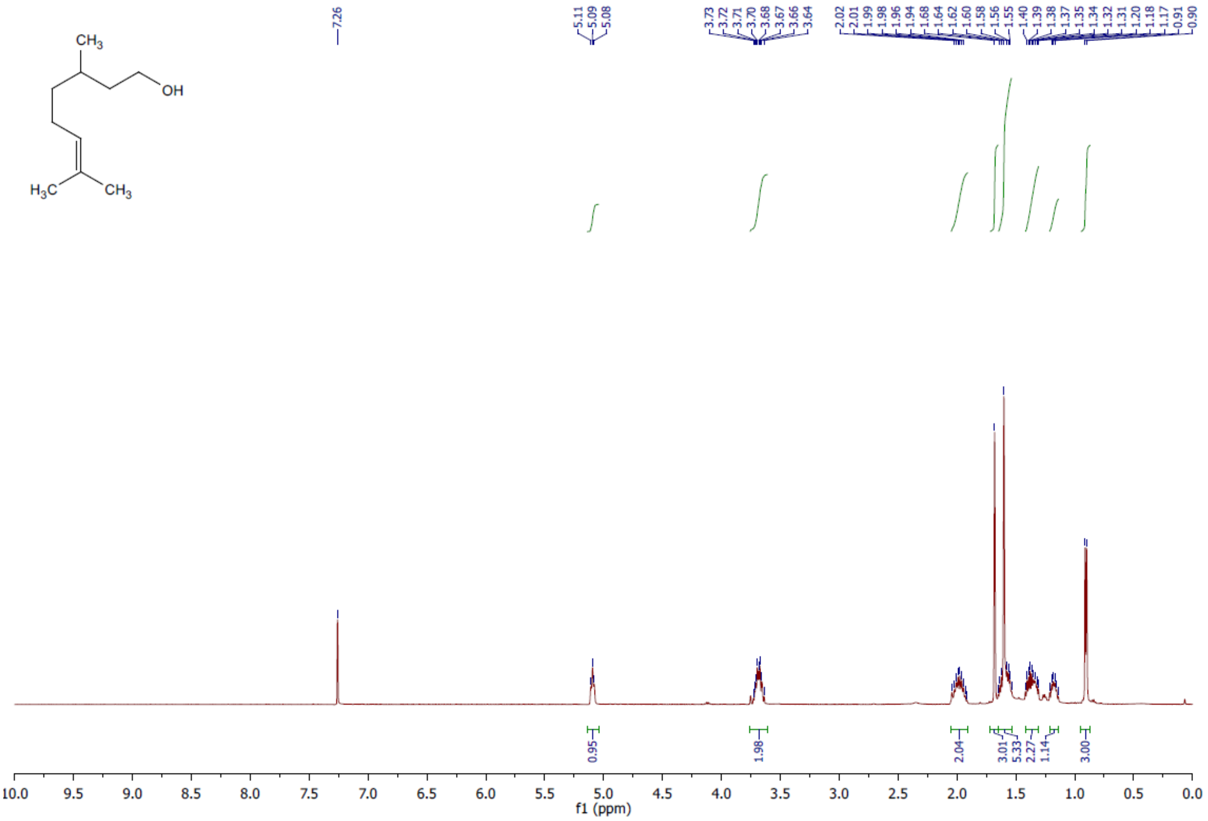

Figure S37. ^{1}H-NMR spectrum of alcohol **4s**.

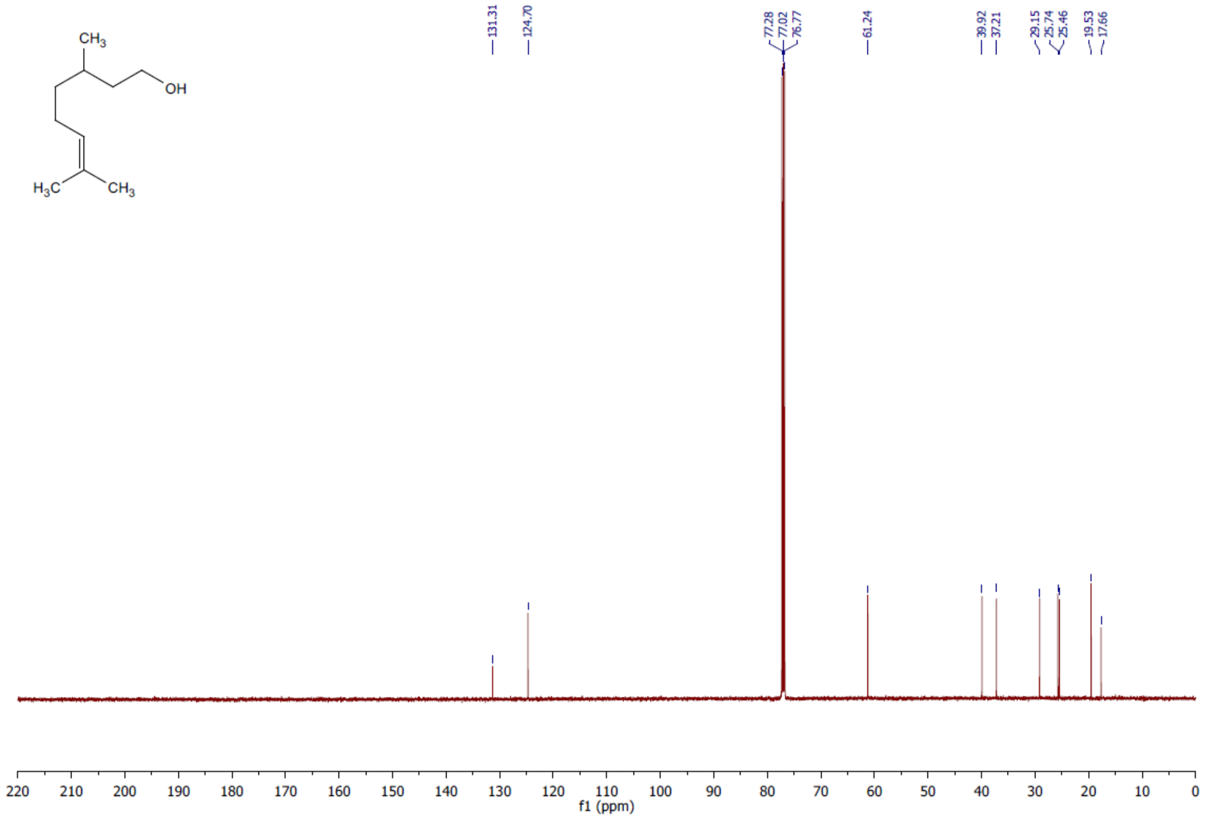

Figure S38. ^{13}C-NMR spectrum of alcohol **4s**.

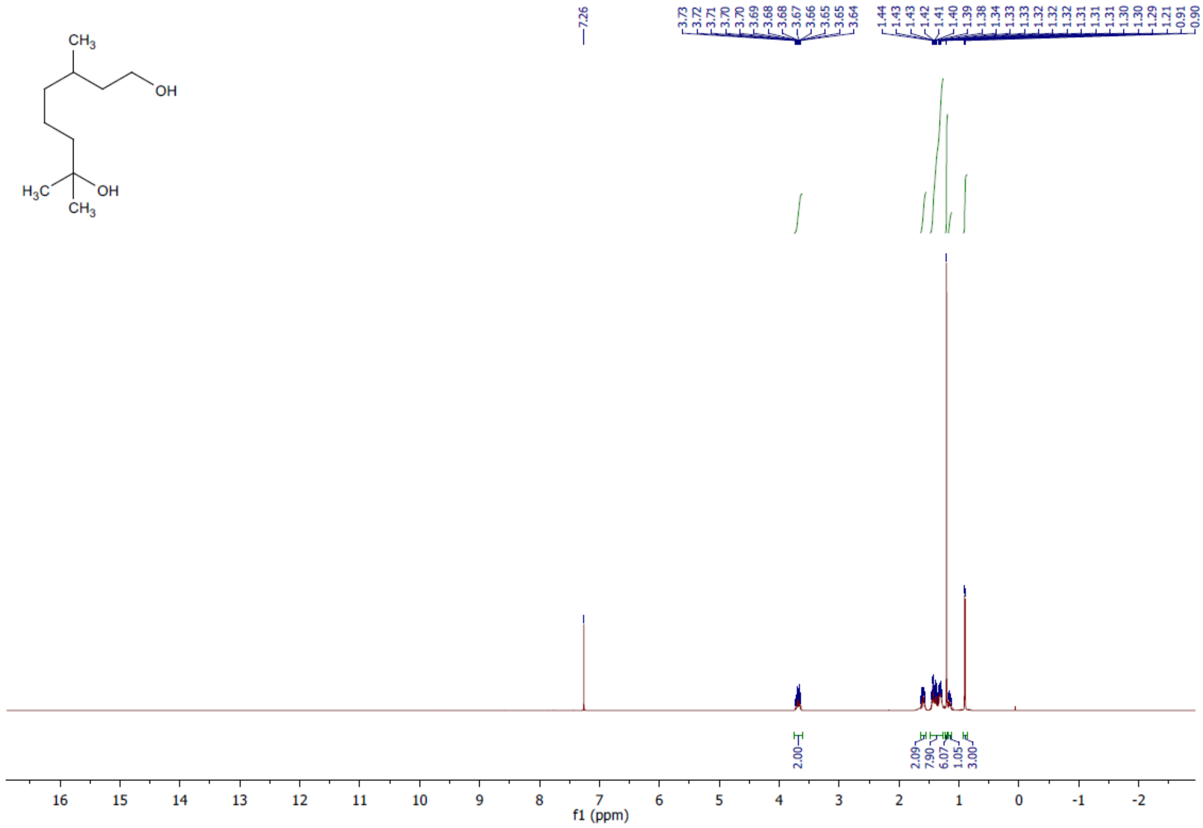

Figure S39. ^{1}H-NMR spectrum of alcohol **4t**.

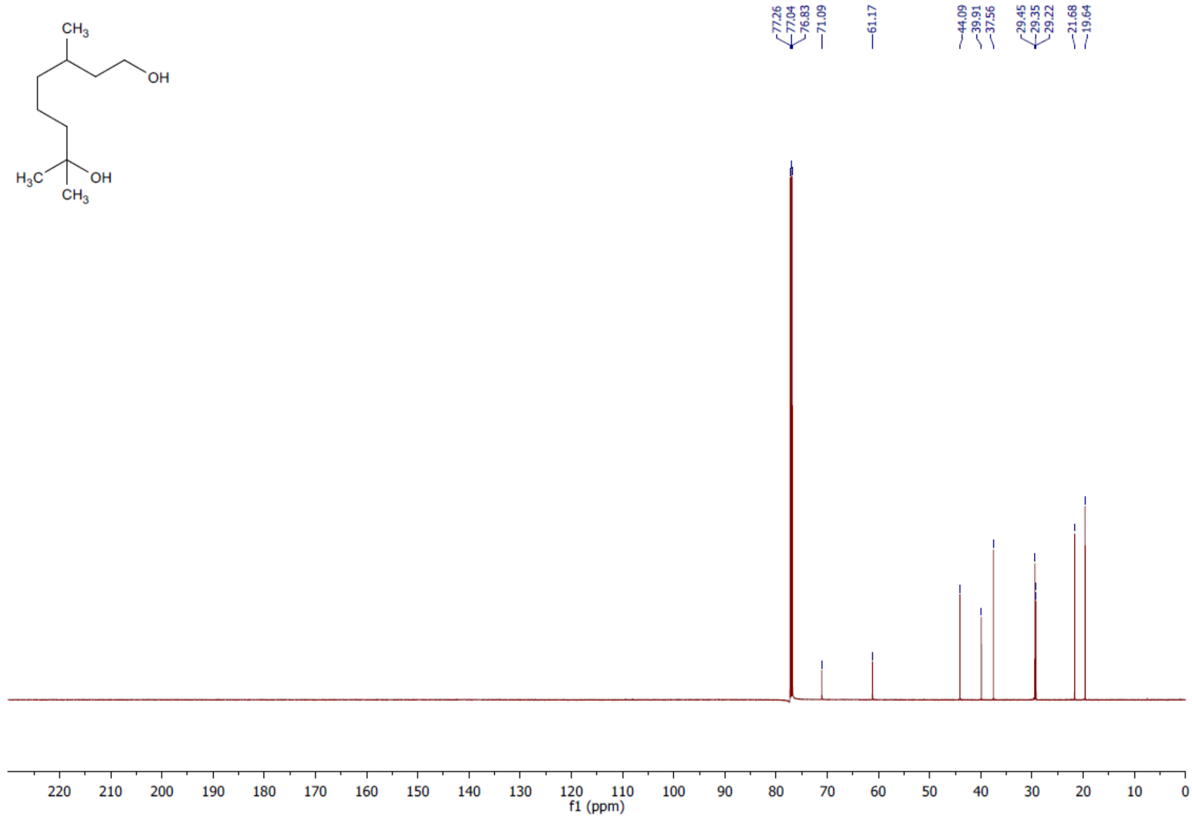

Figure S40. ^{13}C-NMR spectrum of alcohol **4t**.

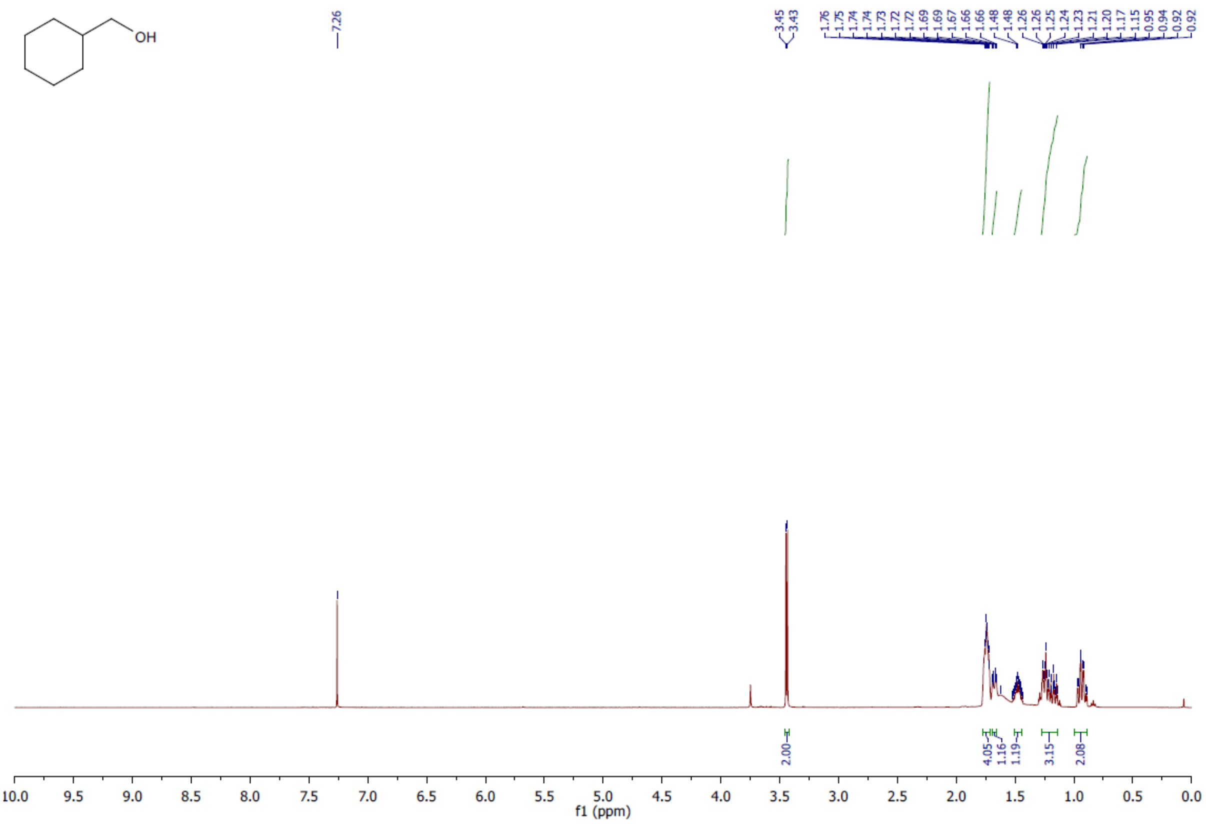

Figure S41. ^{1}H-NMR spectrum of alcohol **4u**.

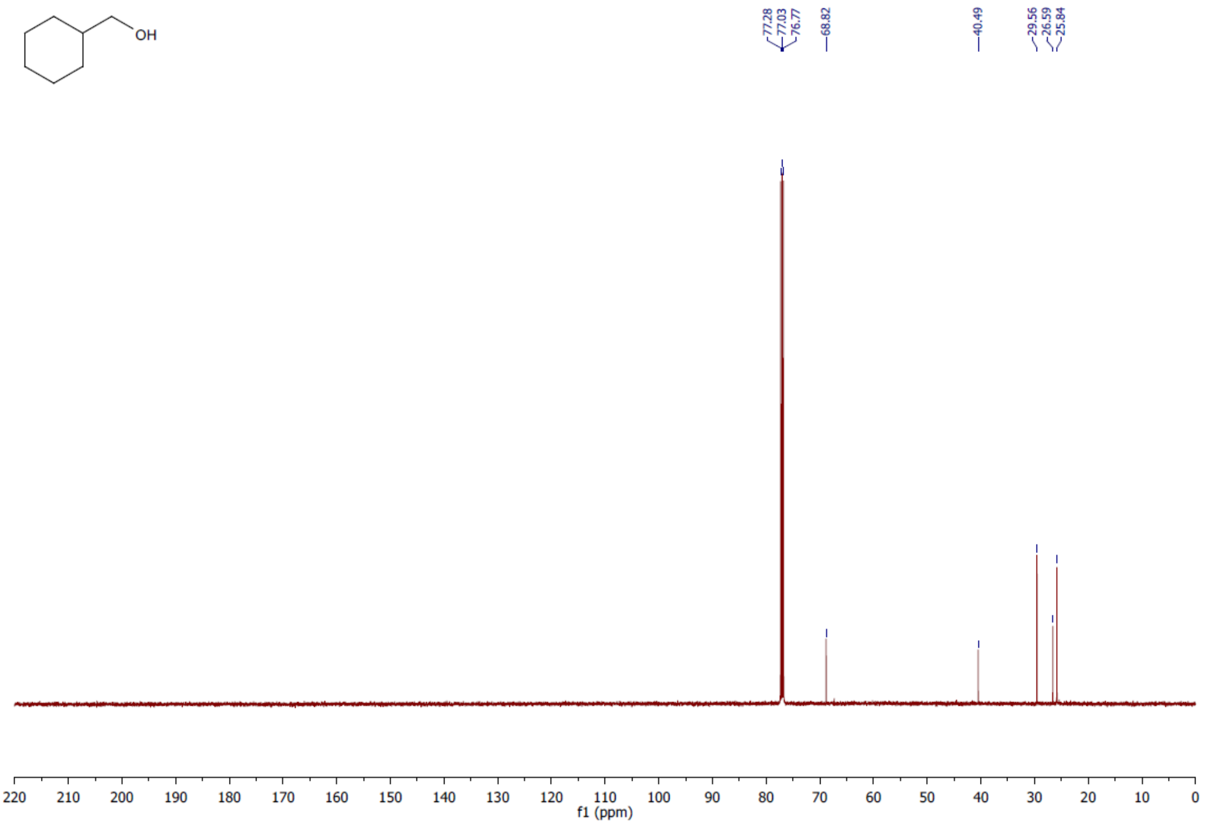

Figure S42. ^{13}C-NMR spectrum of alcohol **4u**.

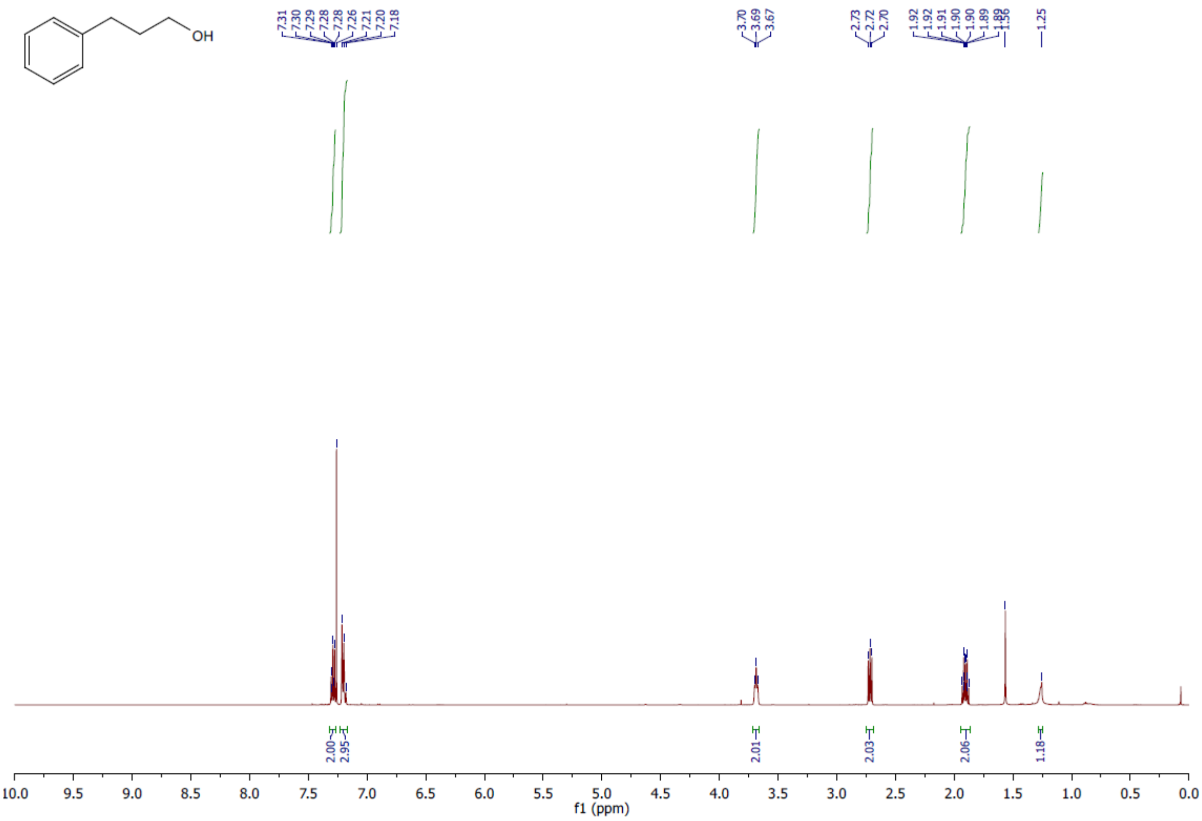

Figure S43. ^{1}H-NMR spectrum of alcohol **4v**.

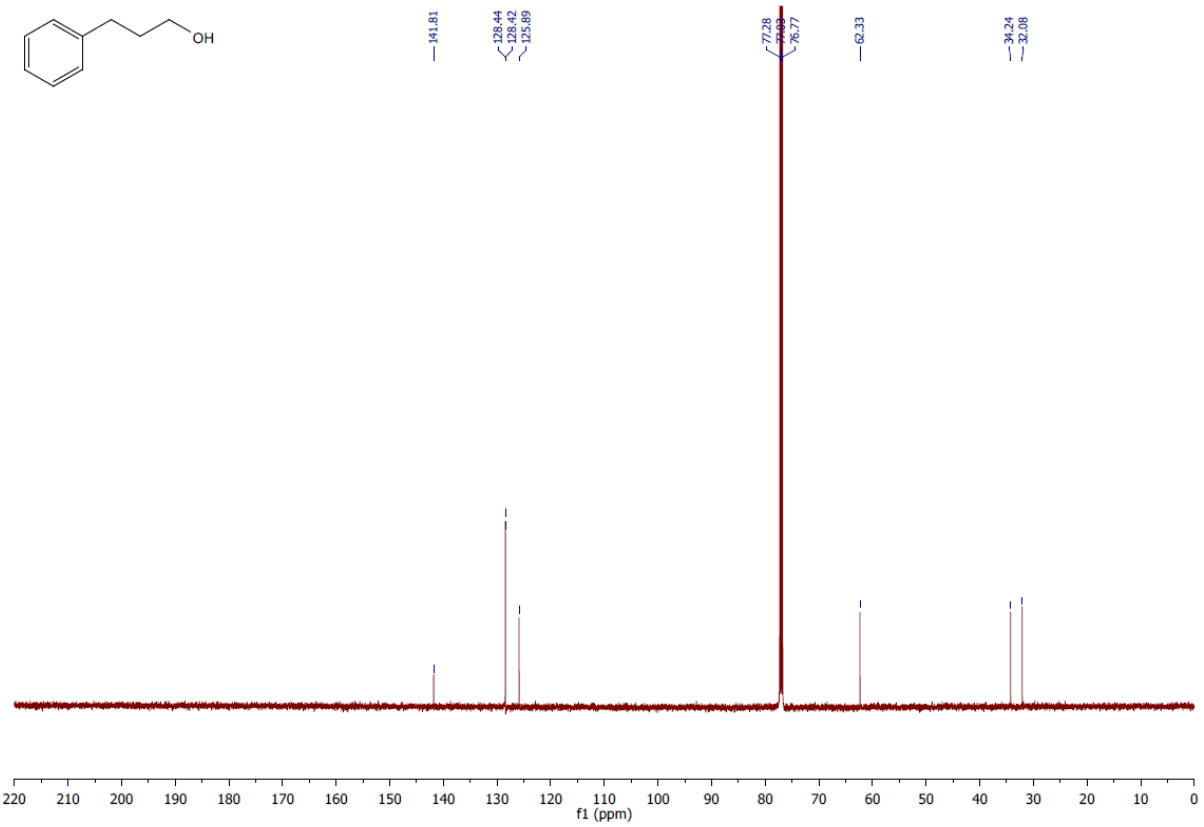

Figure S44. ^{13}C-NMR spectrum of alcohol **4v**.

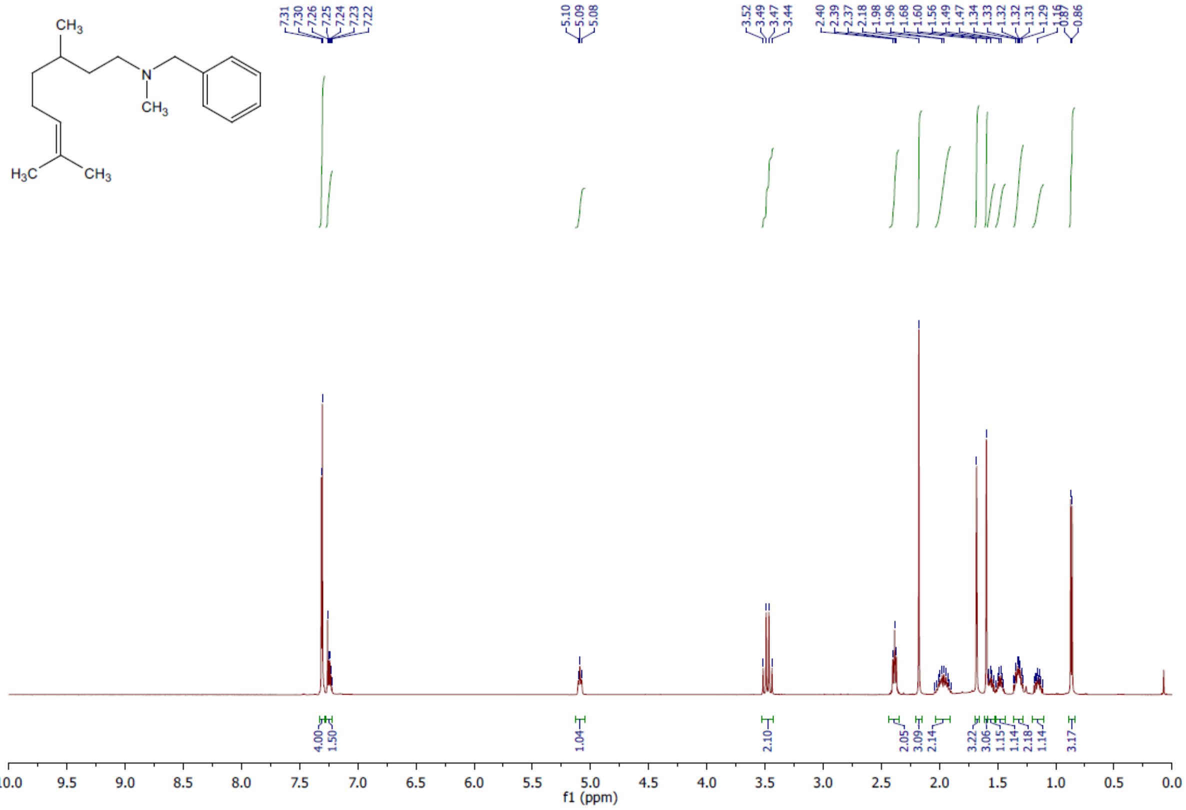

Figure S45. ^{1}H-NMR spectrum of amine **5a**.

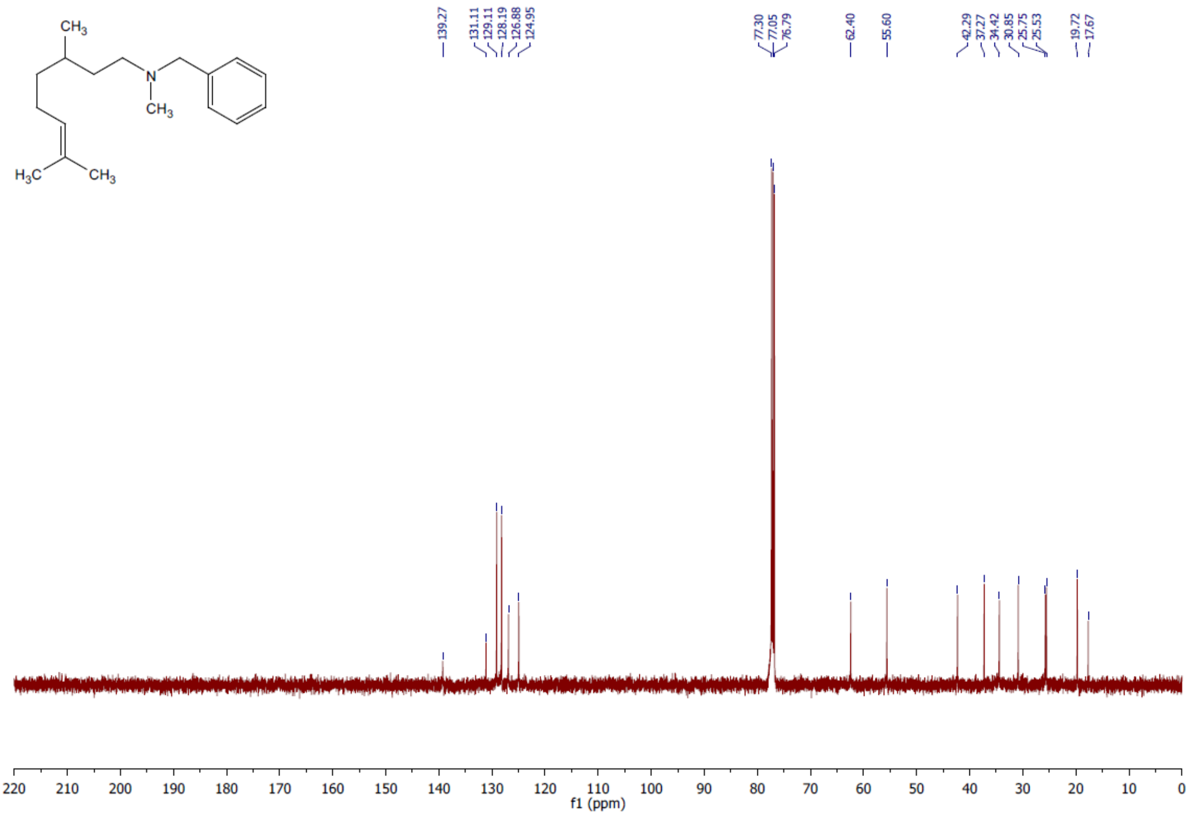

Figure S46. ^{13}C-NMR spectrum of amine **5a**.

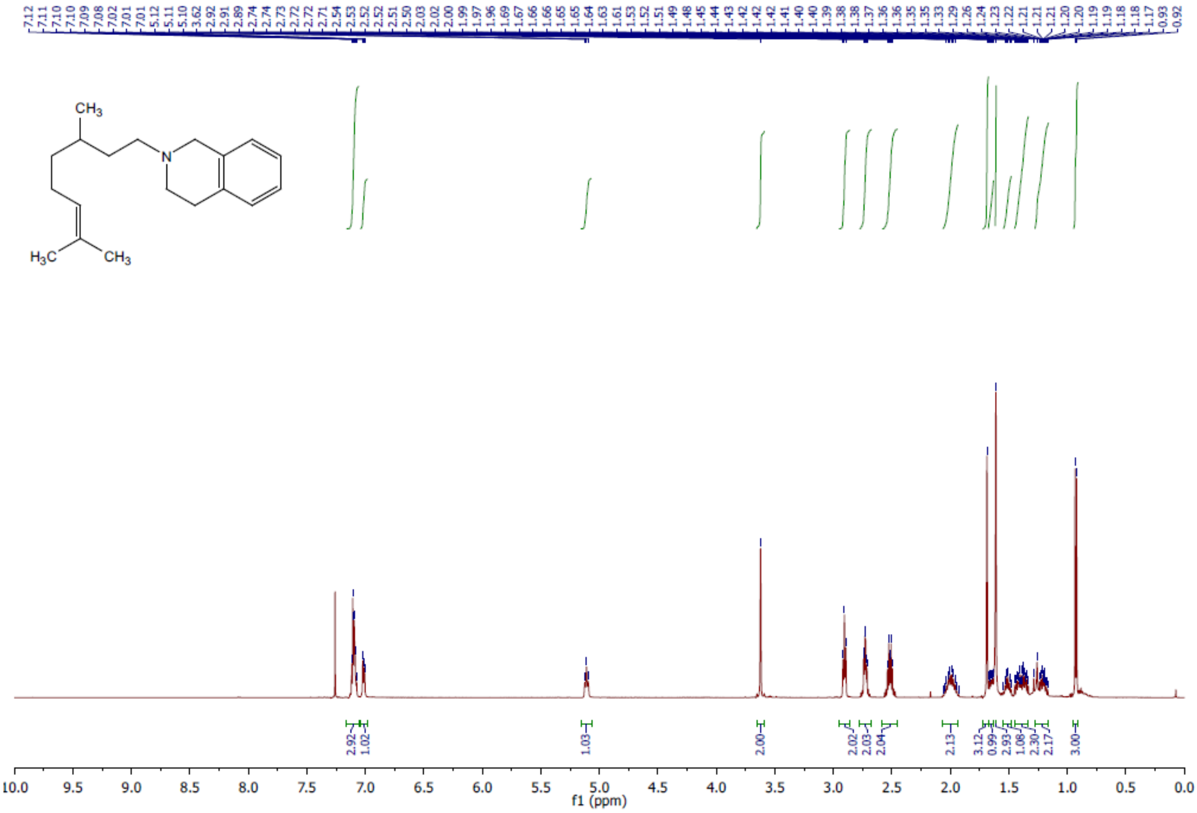

Figure S47. ^{1}H-NMR spectrum of amine **4b**.

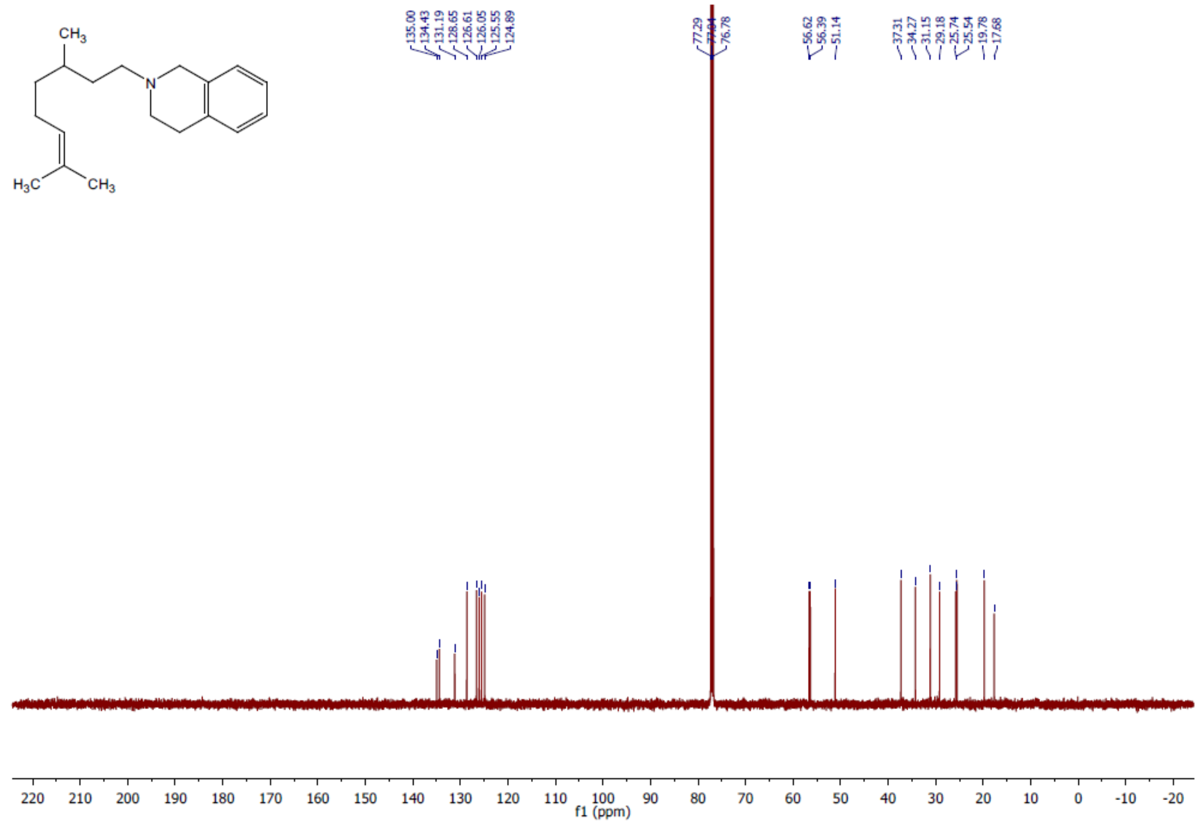

Figure S48. ^{13}C-NMR spectrum of amine **5b**.

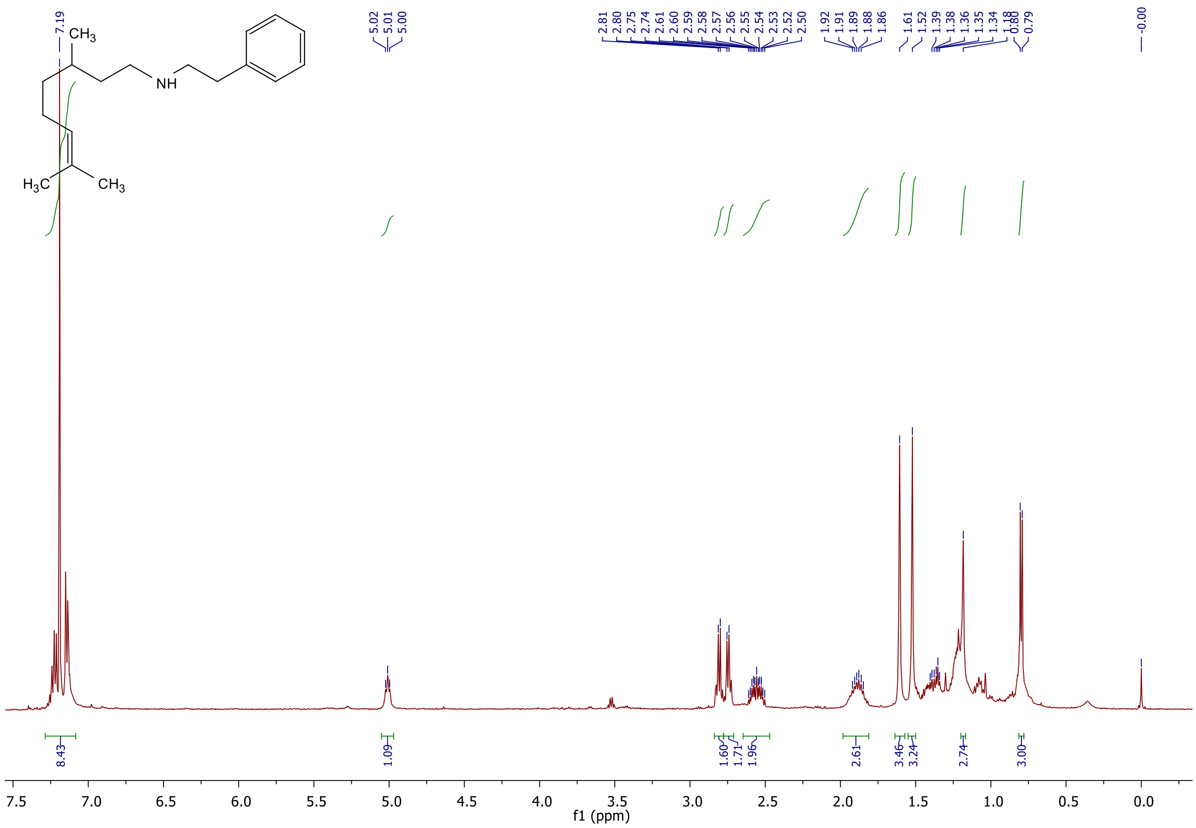

Figure S49. ^{1}H-NMR spectrum of amine **5c**.

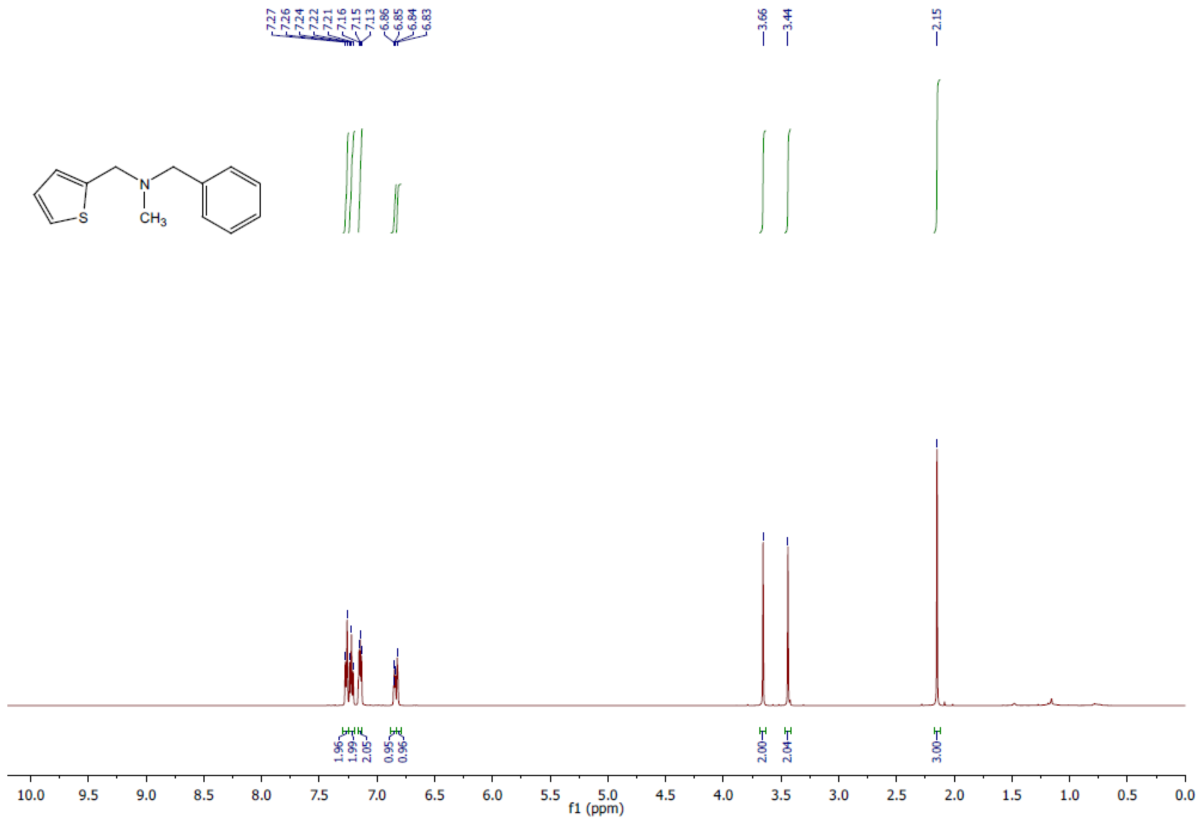

Figure S50. ^{1}H-NMR spectrum of amine **5d**.

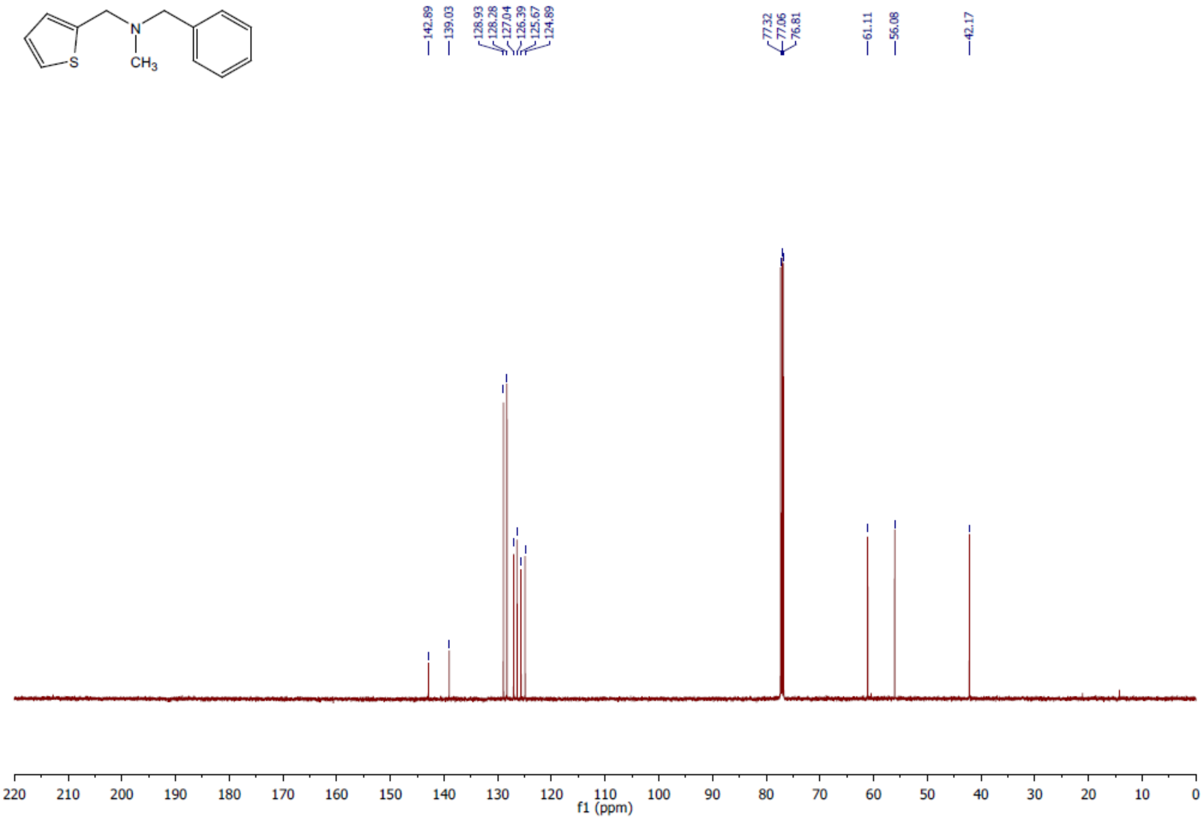

Figure S51. ^{13}C-NMR spectrum of amine **5d**.

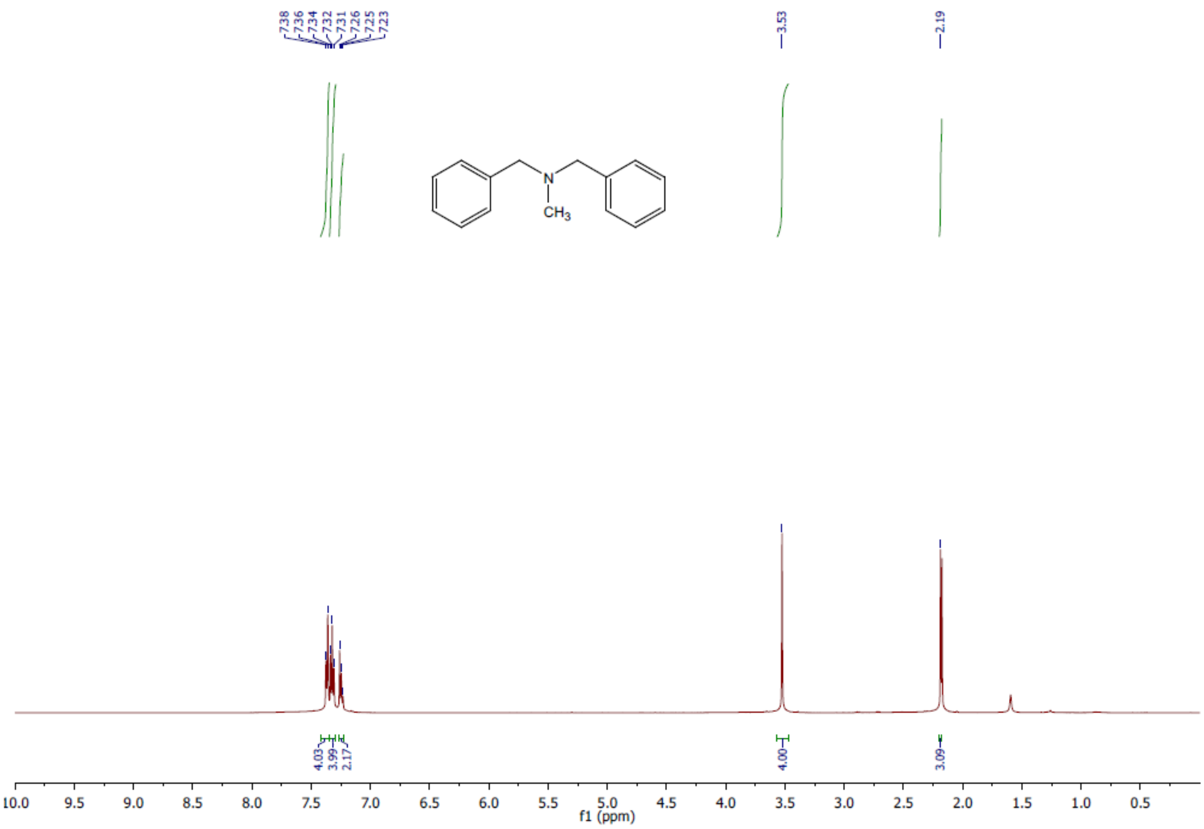

Figure S52. ^{1}H-NMR spectrum of amine **5e**.

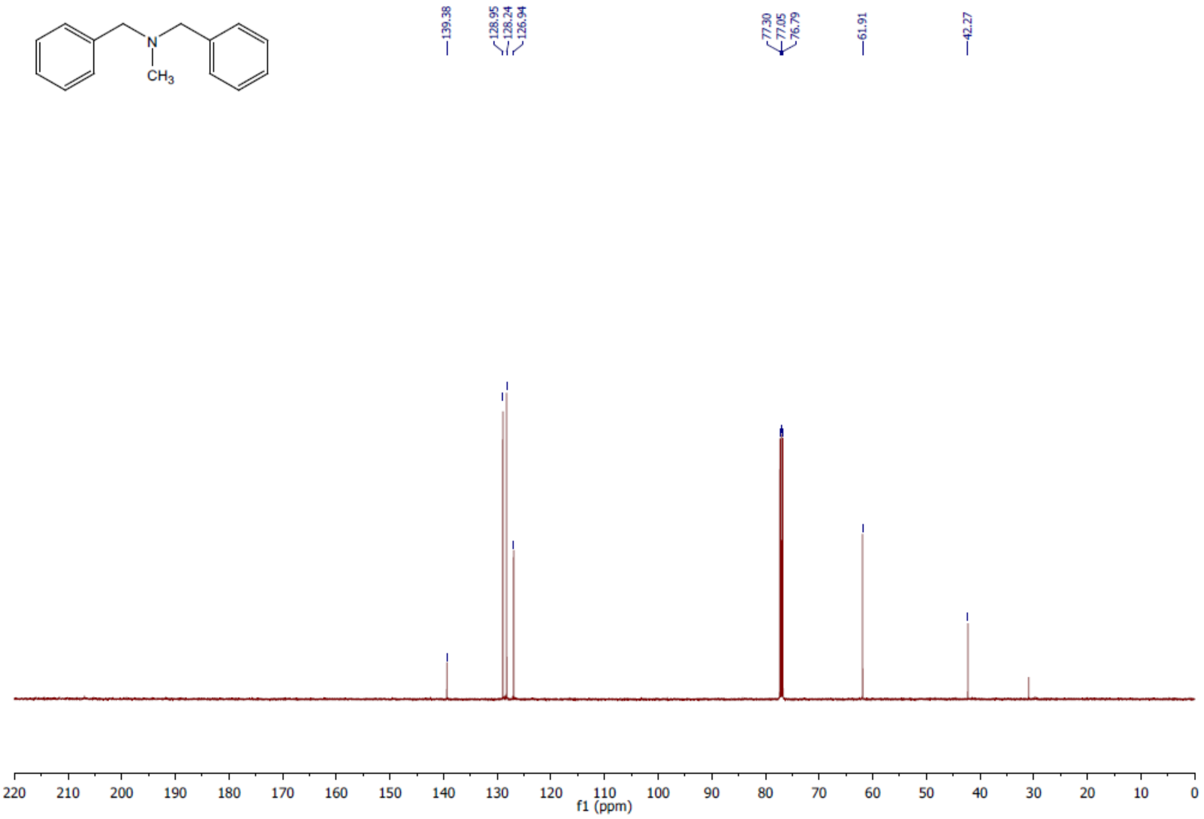

Figure S53. ^{13}C-NMR spectrum of amine **5e**.

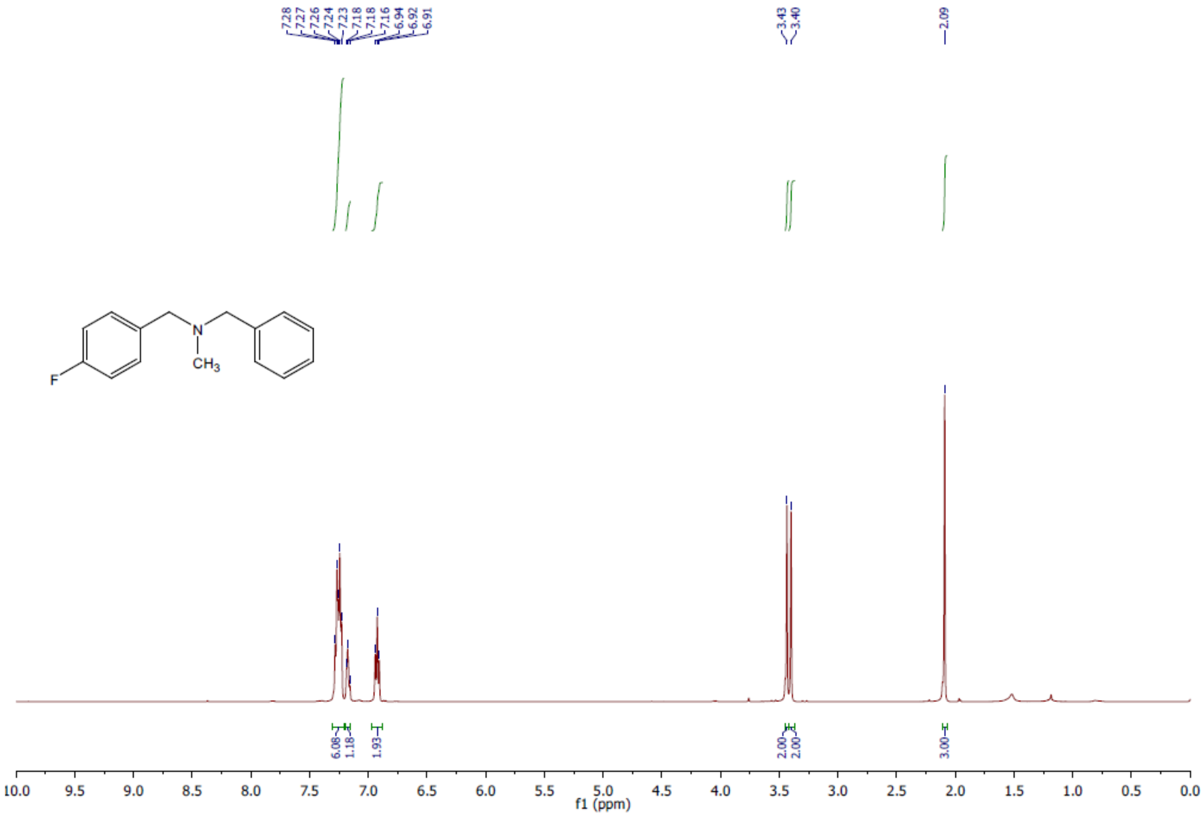

Figure S54. ^{1}H-NMR spectrum of amine **5f**.

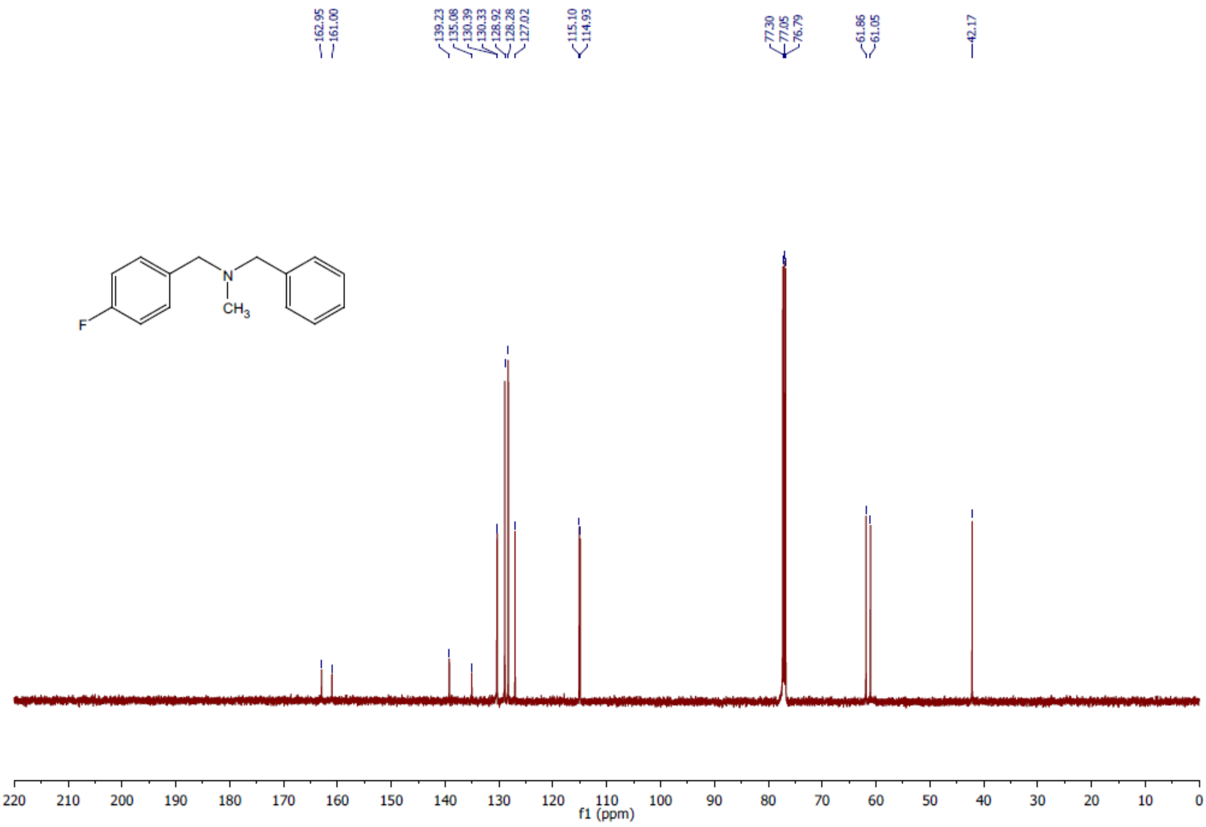

Figure S55. ^{13}C-NMR spectrum of amine **5f**.

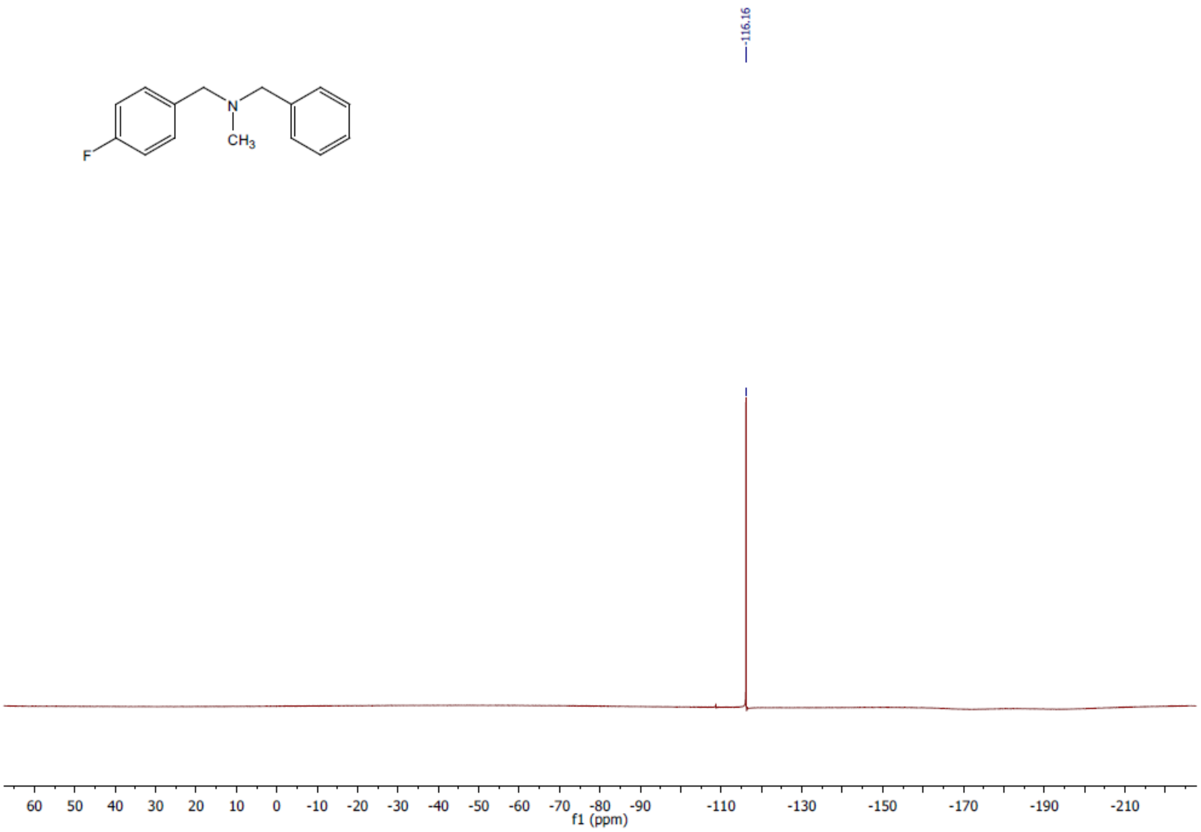

Figure S56. ^{19}F-NMR spectrum of amine **5f**.

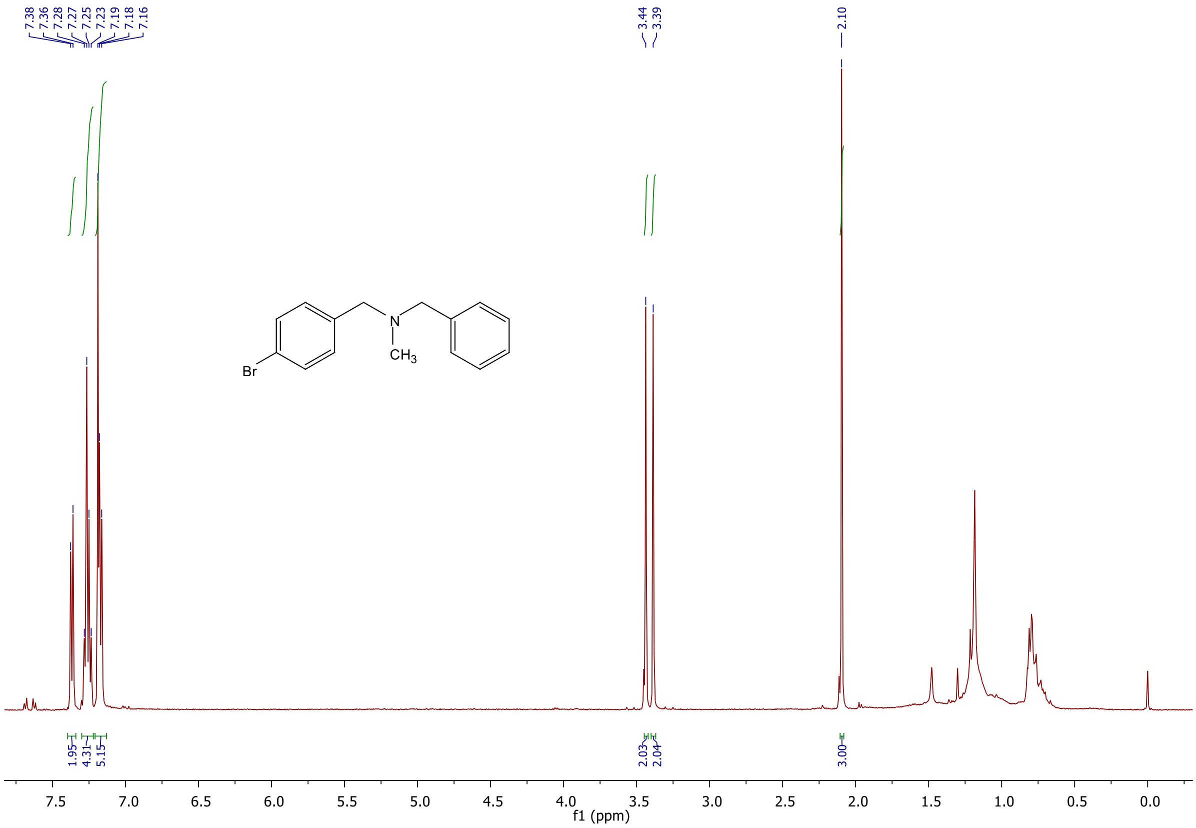

Figure S57. ^{1}H-NMR spectrum of amine **5g**.

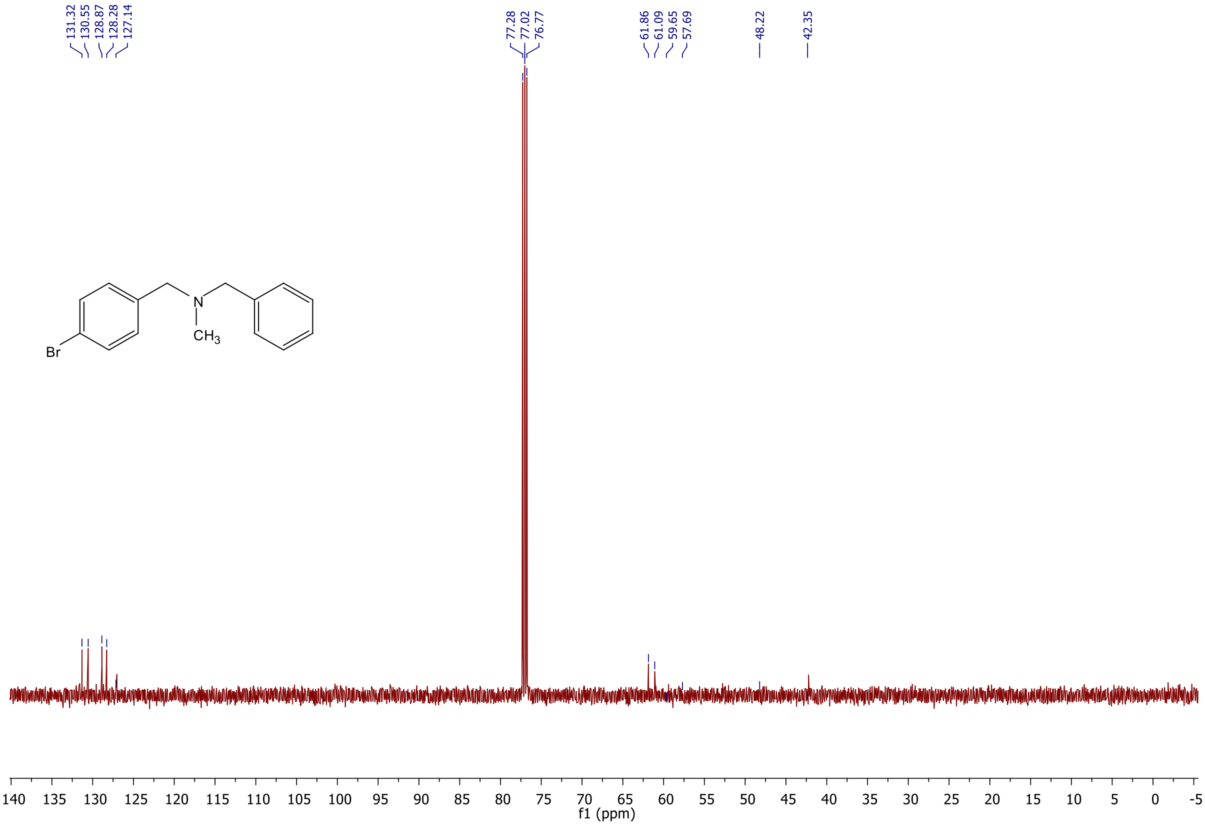

Figure S58. ^{13}C-NMR spectrum of amine **5g**.

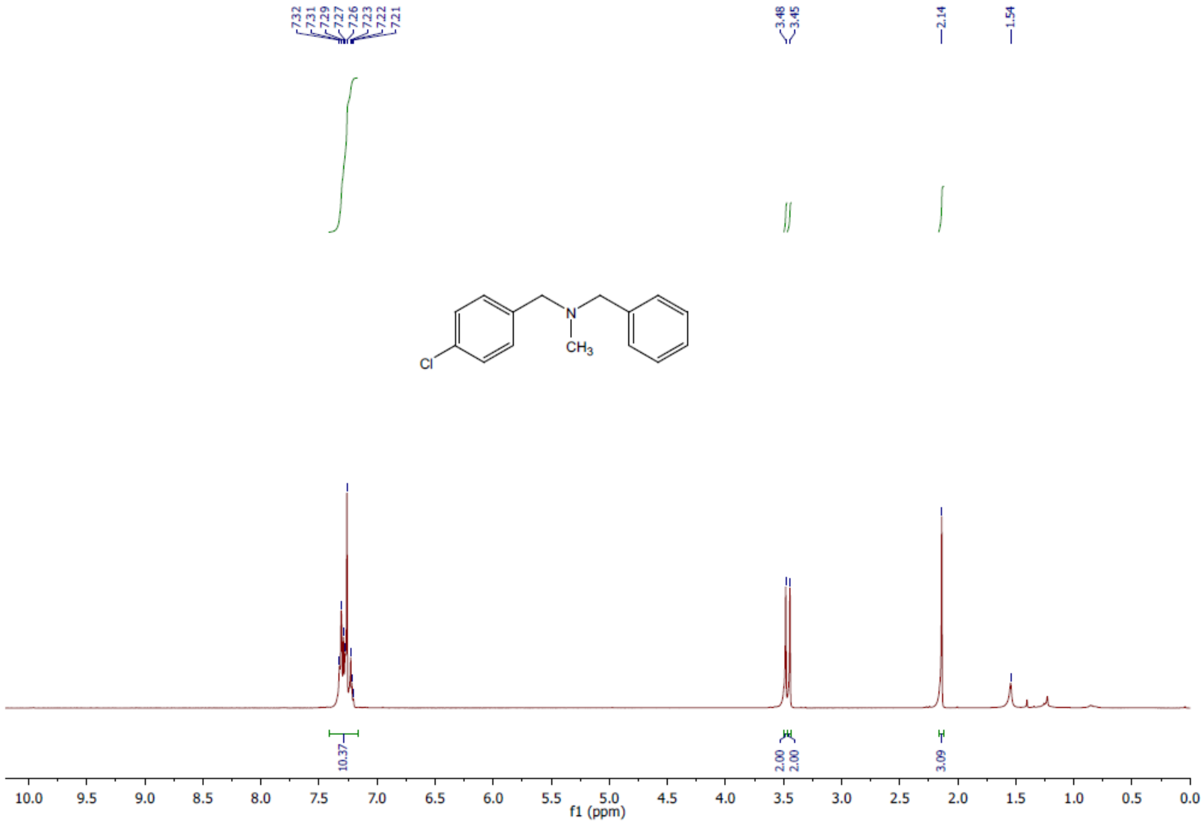

Figure S59. ^{1}H-NMR spectrum of amine **5h**.

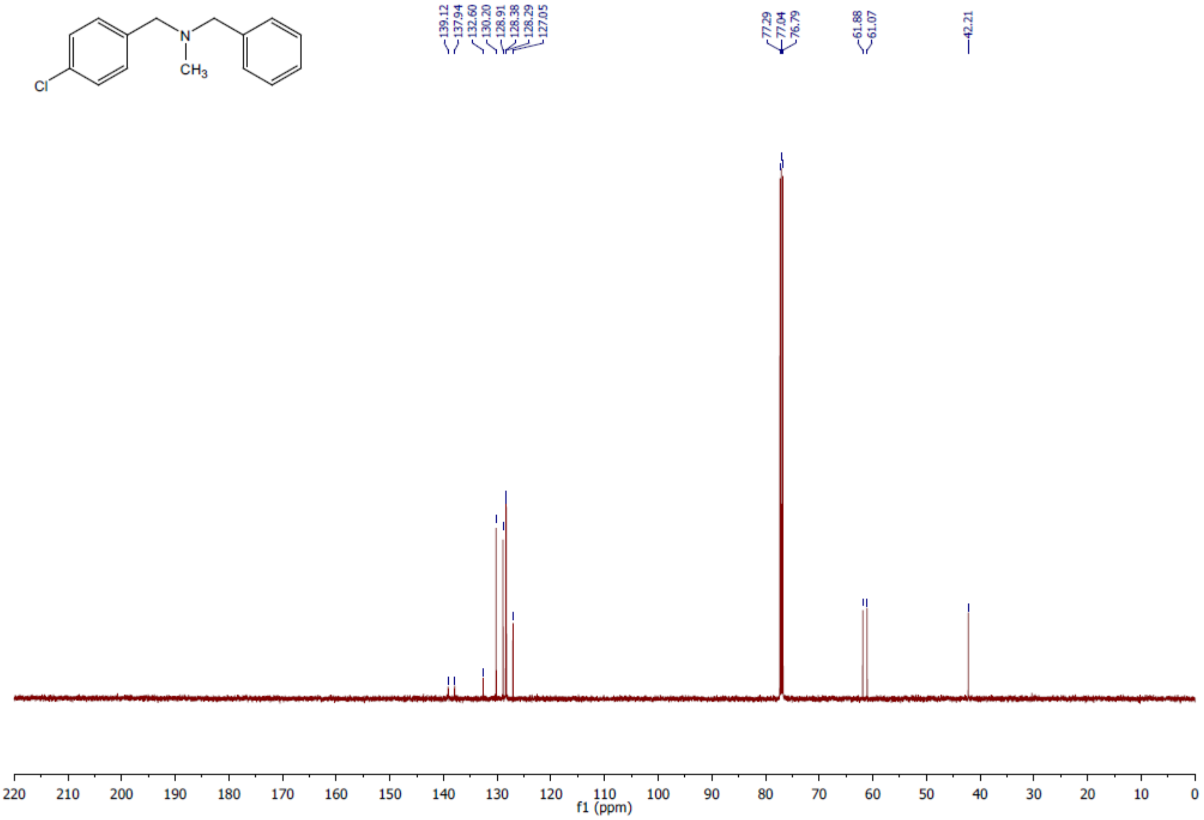

Figure S60. ^{13}C-NMR spectrum of amine **5h**.

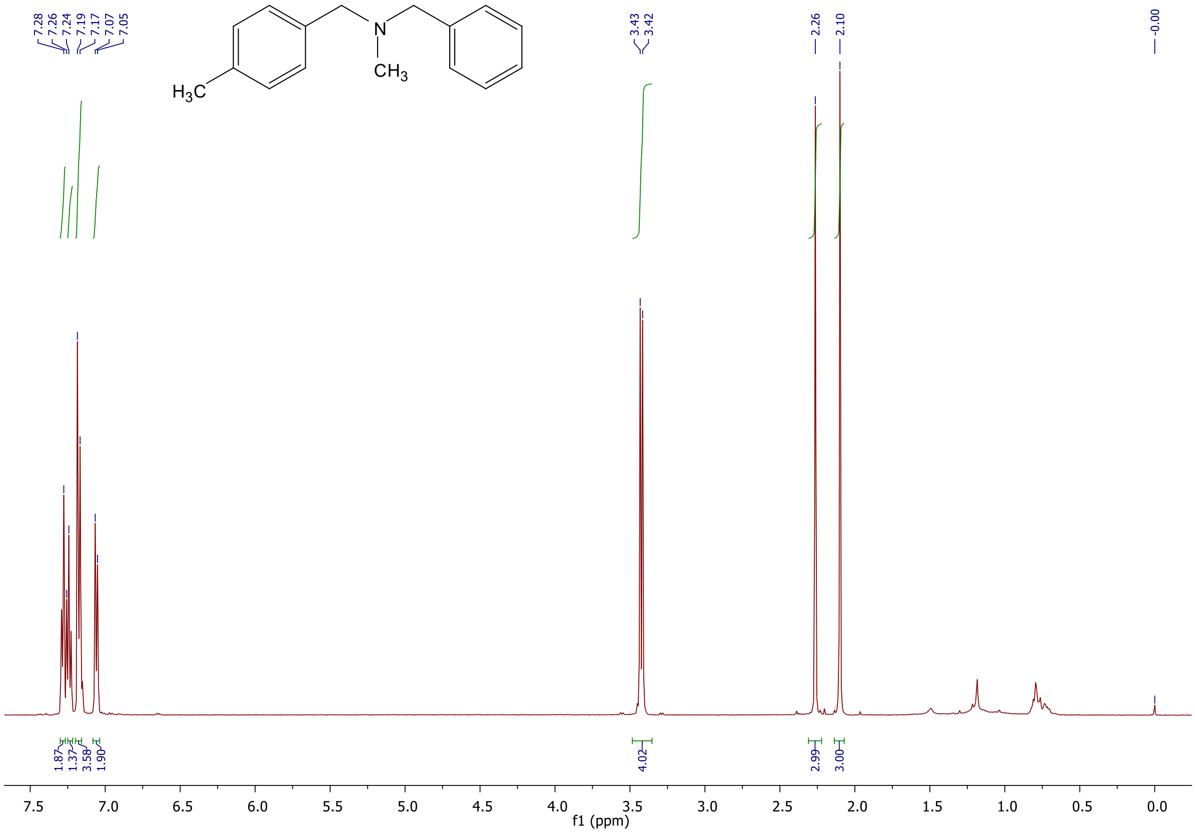

Figure S61. ^{1}H-NMR spectrum of amine **5i**.

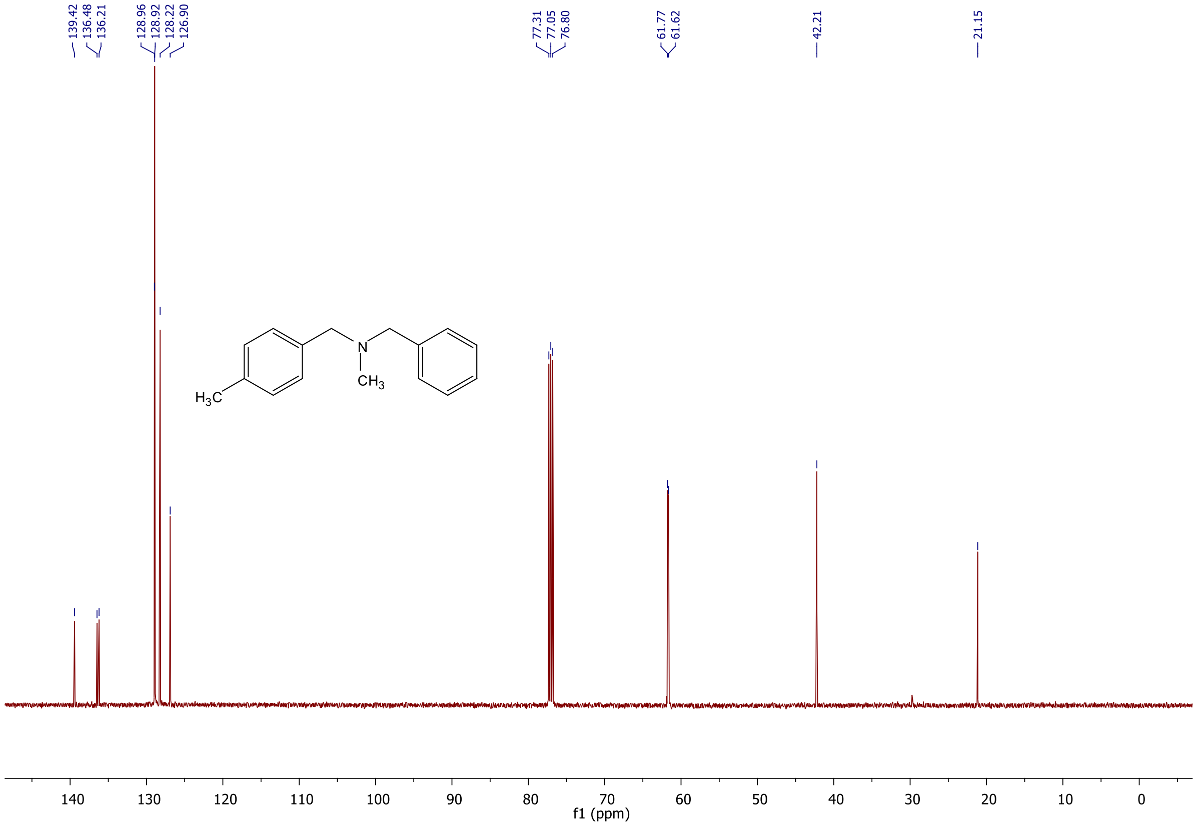

Figure S62. ^{13}C-NMR spectrum of amine **5i**.

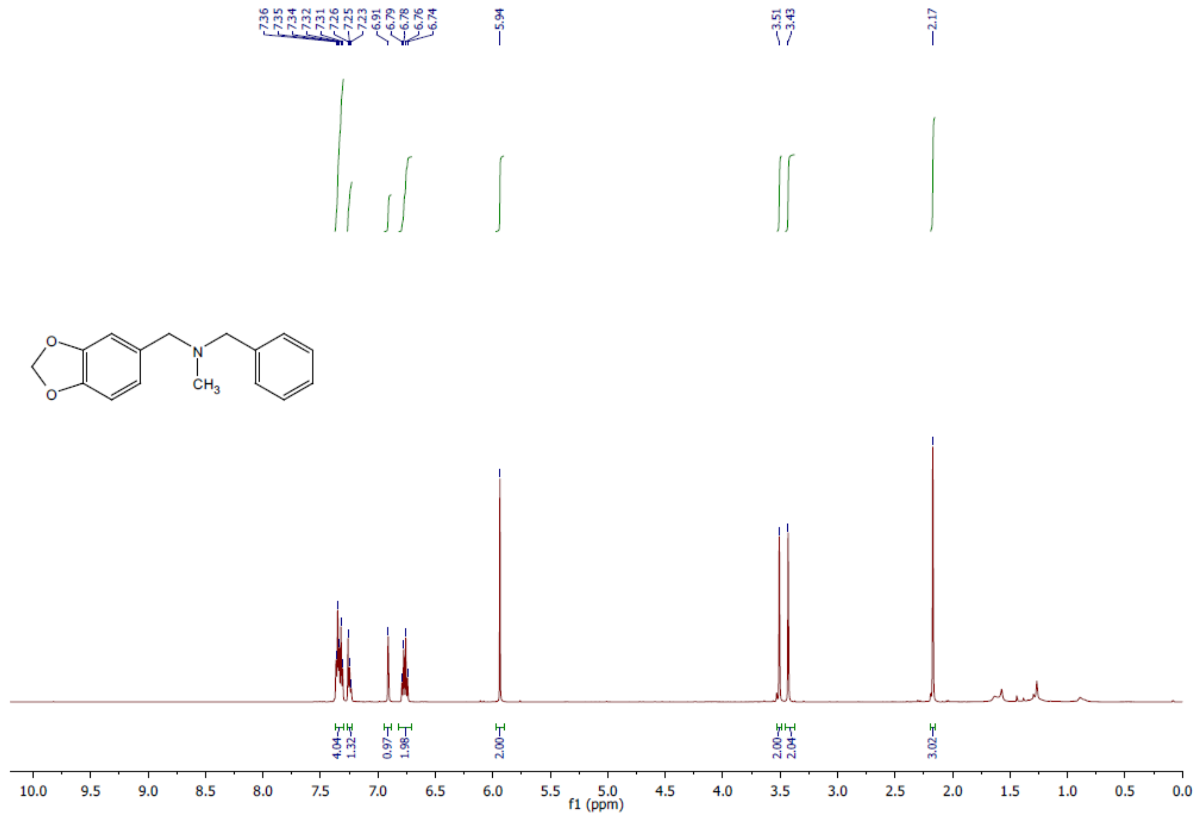

Figure S63. ^{1}H-NMR spectrum of amine **5j**.

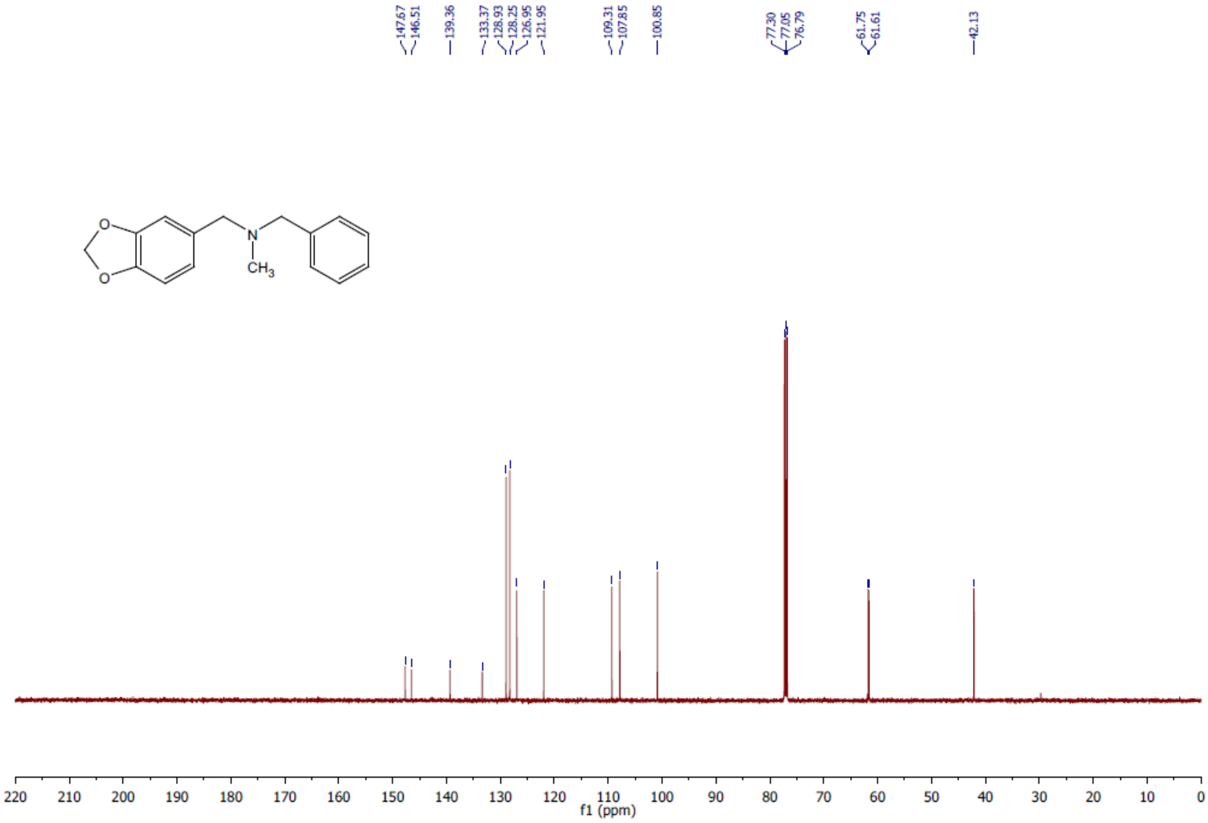

Figure S64. ^{13}C-NMR spectrum of amine **5j**.

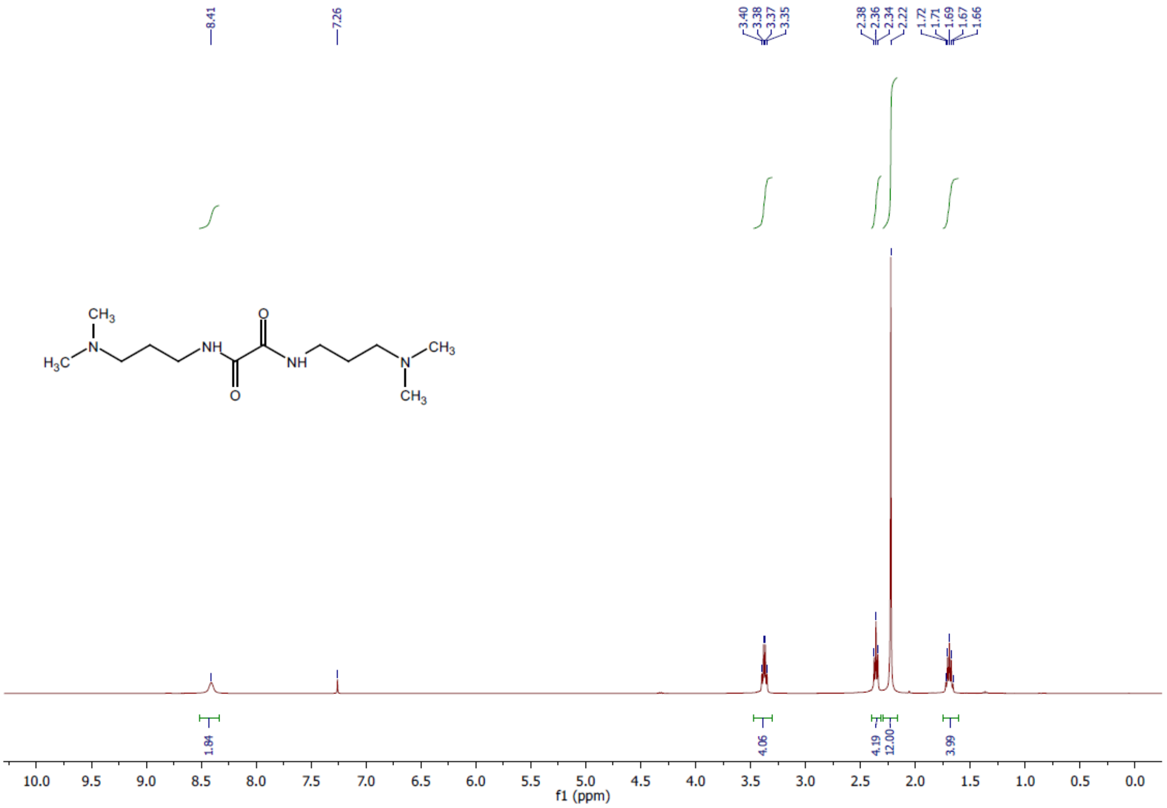

Figure S65: ^{1}H-NMR spectrum of N^1,N^2-bis(3-(dimethylamino)propyl)oxalamide (**5**).

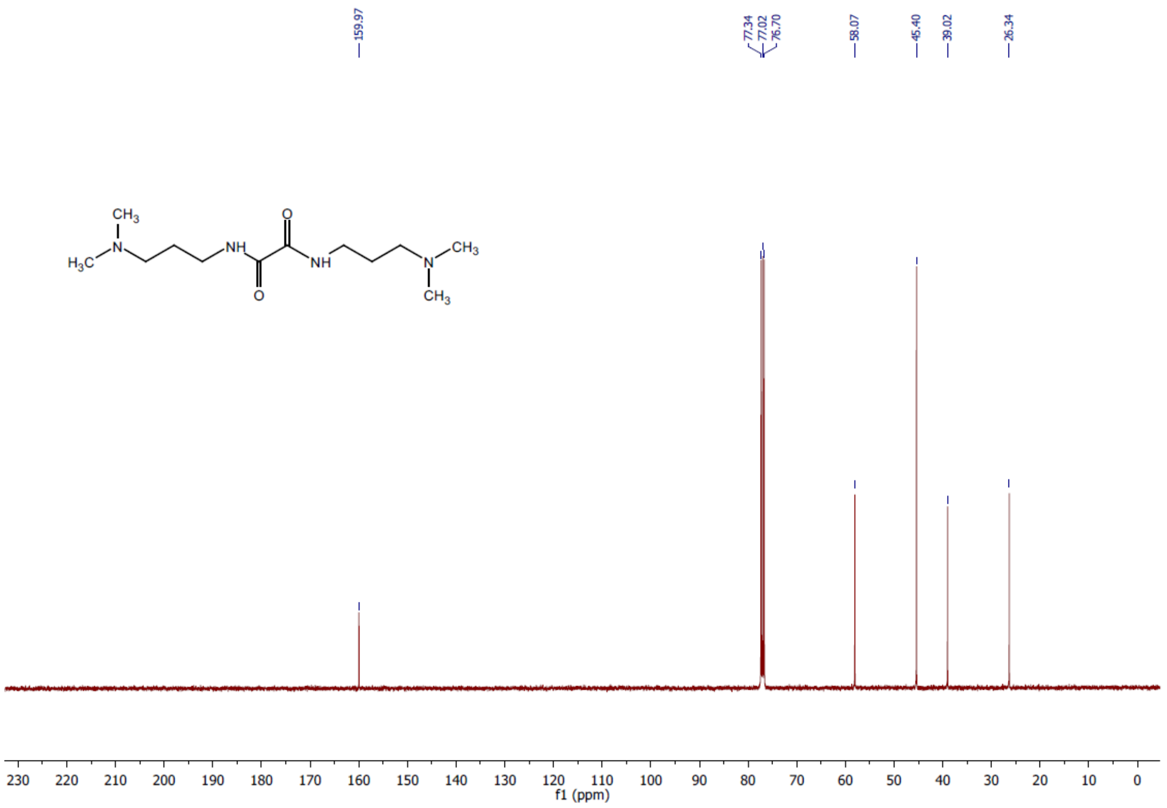

Figure S66: ^{13}C-NMR spectrum of N^1,N^2-bis(3-(dimethylamino)propyl)oxalamide (**5**).

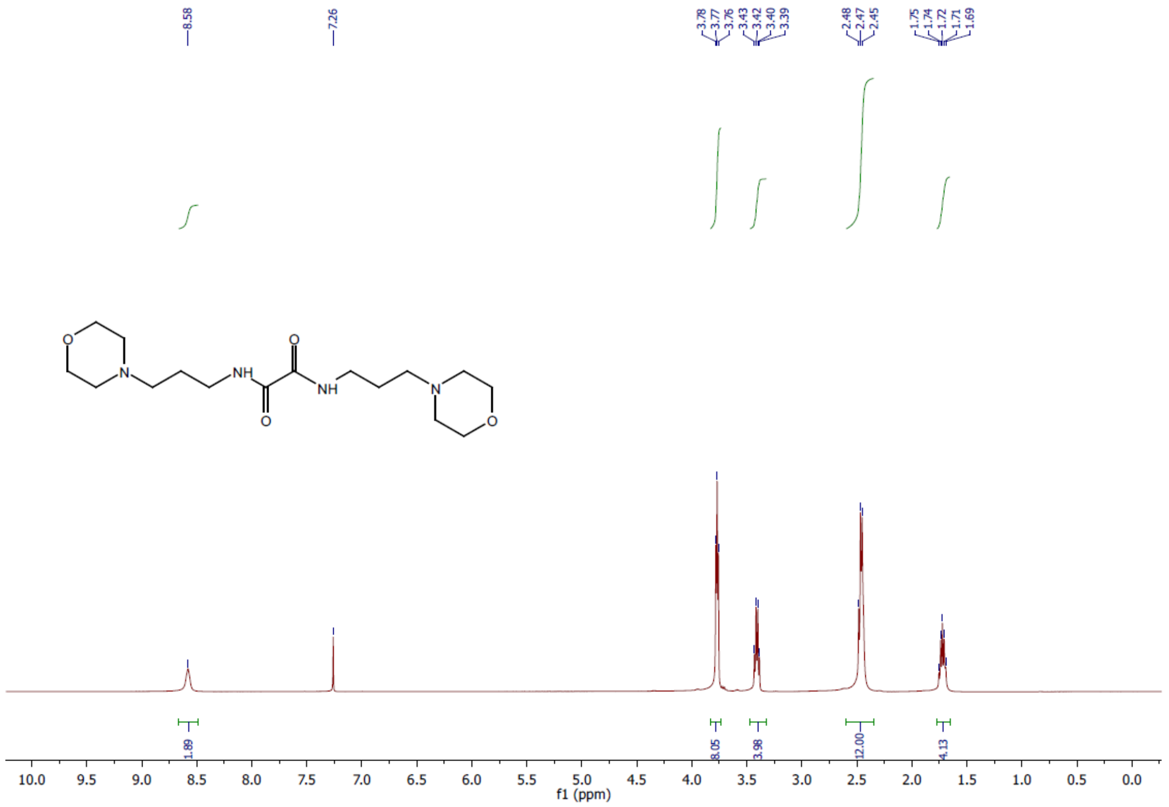

Figure S67: ^{1}H-NMR spectrum of N^{1},N^{2}-bis(3-morpholinopropyl)oxalamide (**6**).

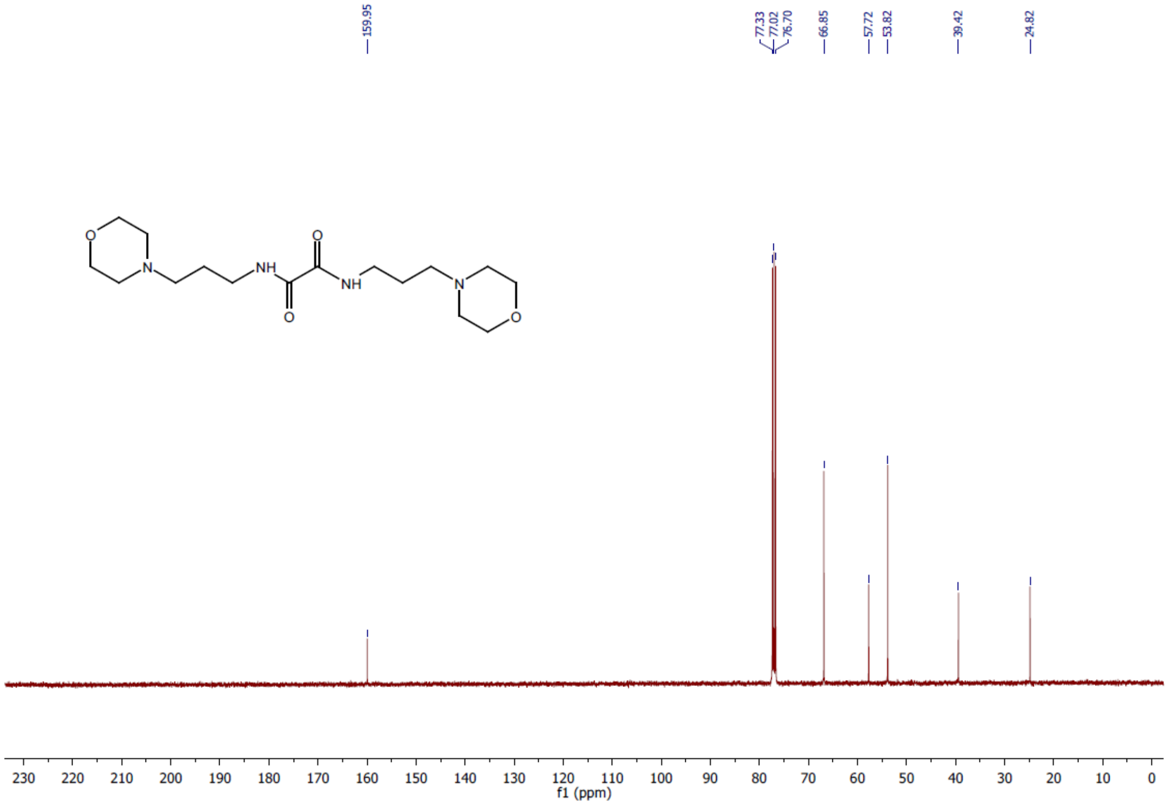

Figure S68: ^{13}C-NMR spectrum of N^{1},N^{2}-bis(3-morpholinopropyl)oxalamide (**6**).

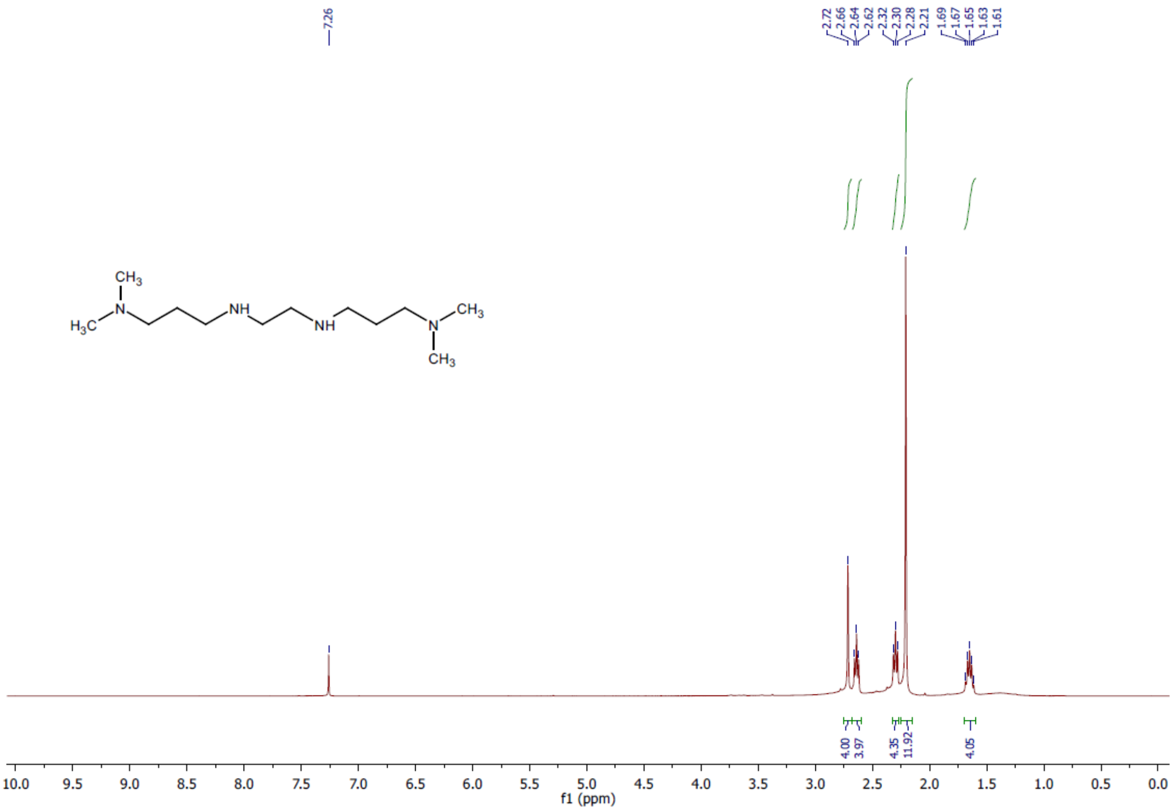

Figure S69: ^{1}H-NMR spectrum of N^{1},$N^{1'}$-(ethane-1,2-diyl)bis(N^{3},N^{3}-dimethylpropane-1,3-diamine) (**3**).

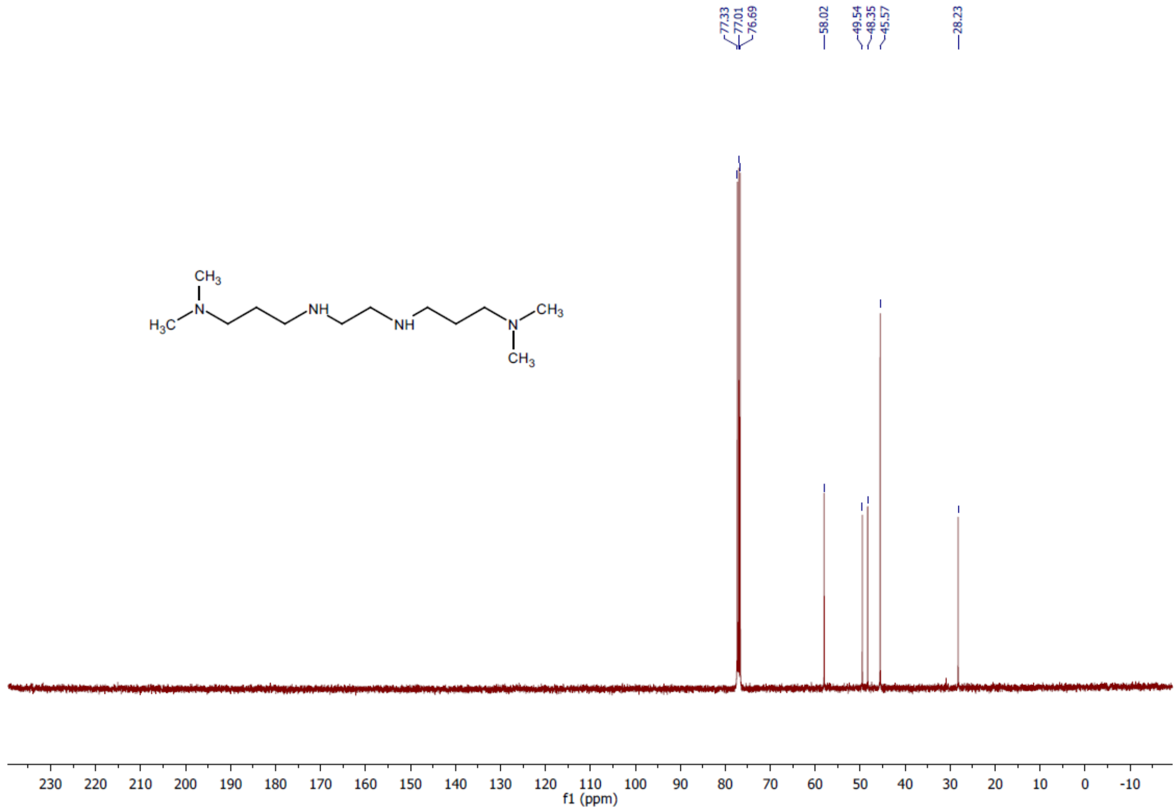

Figure S70: ^{13}C-NMR spectrum of N^{1},$N^{1'}$-(ethane-1,2-diyl)bis(N^{3},N^{3}-dimethylpropane-1,3-diamine) (**3**).

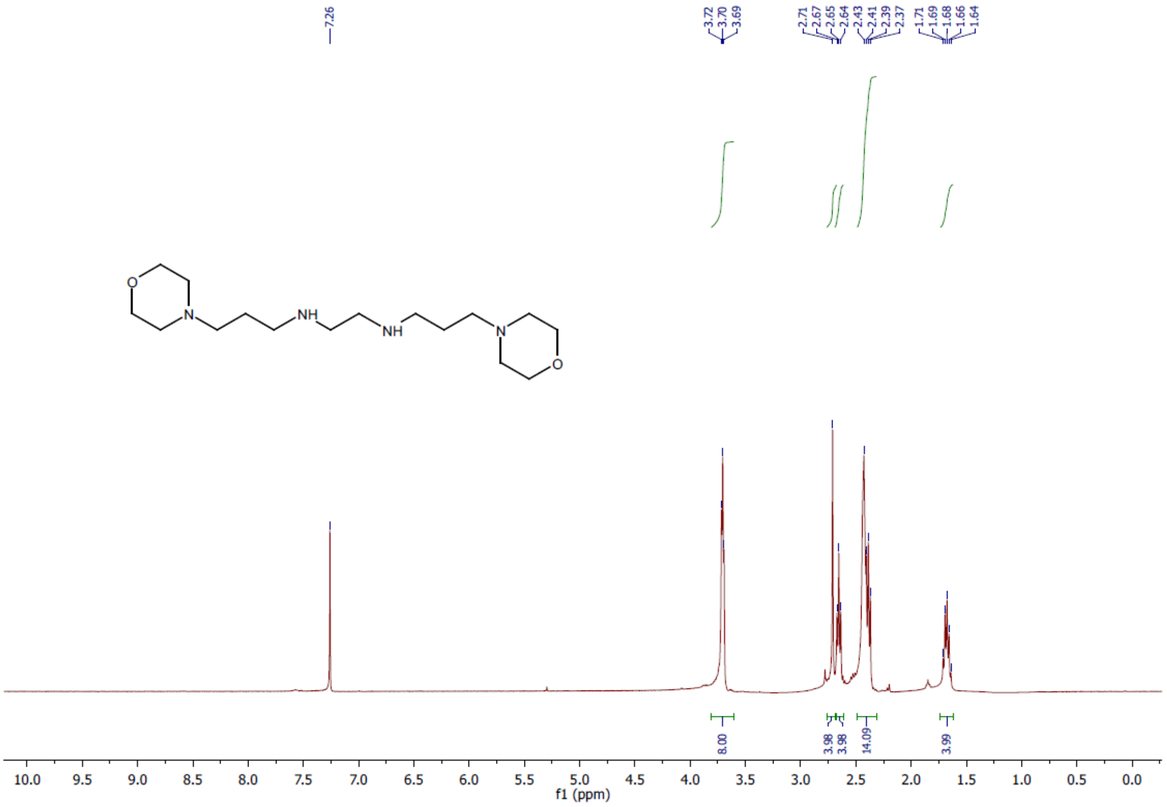

Figure S71: ^{1}H-NMR spectrum of N^1,N^2-bis(3-morpholinopropyl)ethane-1,2-diamine (**4**).

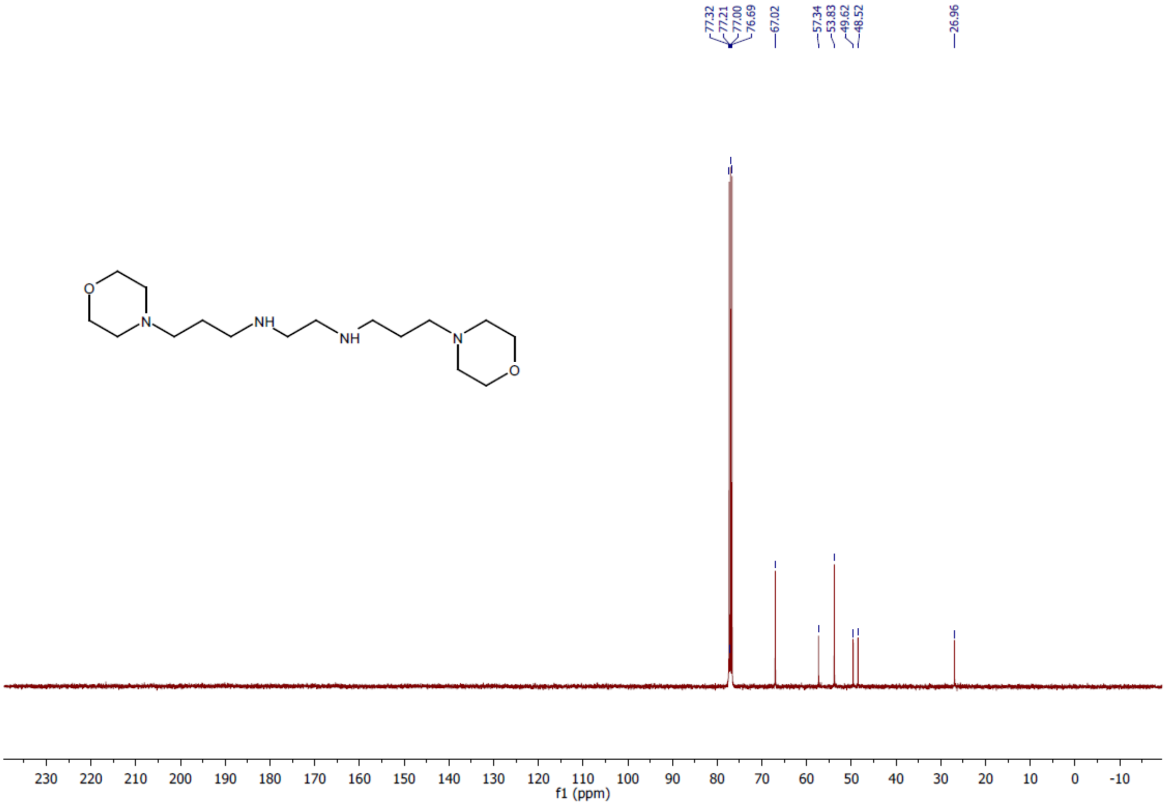

Figure S72: ^{13}C-NMR spectrum of N^1,N^2-bis(3-morpholinopropyl)ethane-1,2-diamine (**4**).

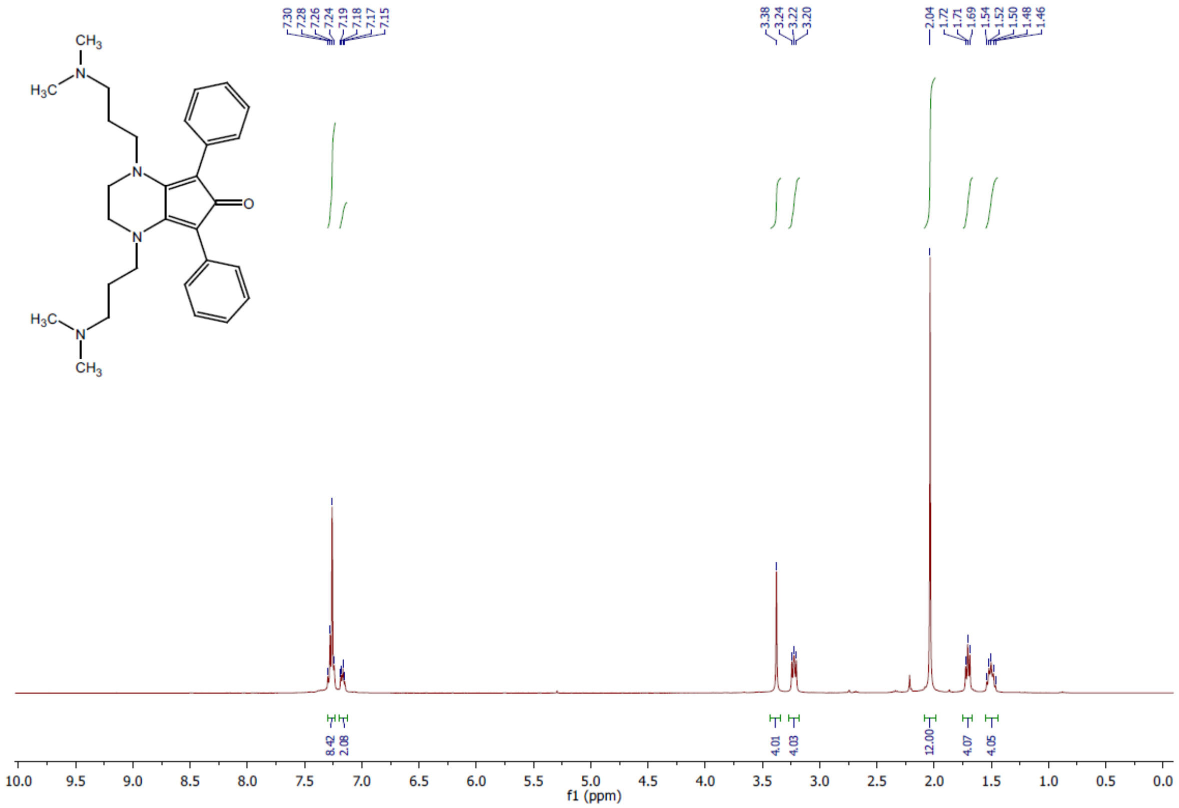

Figure S73: ^{1}H-NMR spectrum of 1,4-bis(3-(dimethylamino)propyl)-5,7-diphenyl-1,2,3,4-tetrahydro-6*H*-cyclopenta[*b*]pyrazin-6-one (**1**).

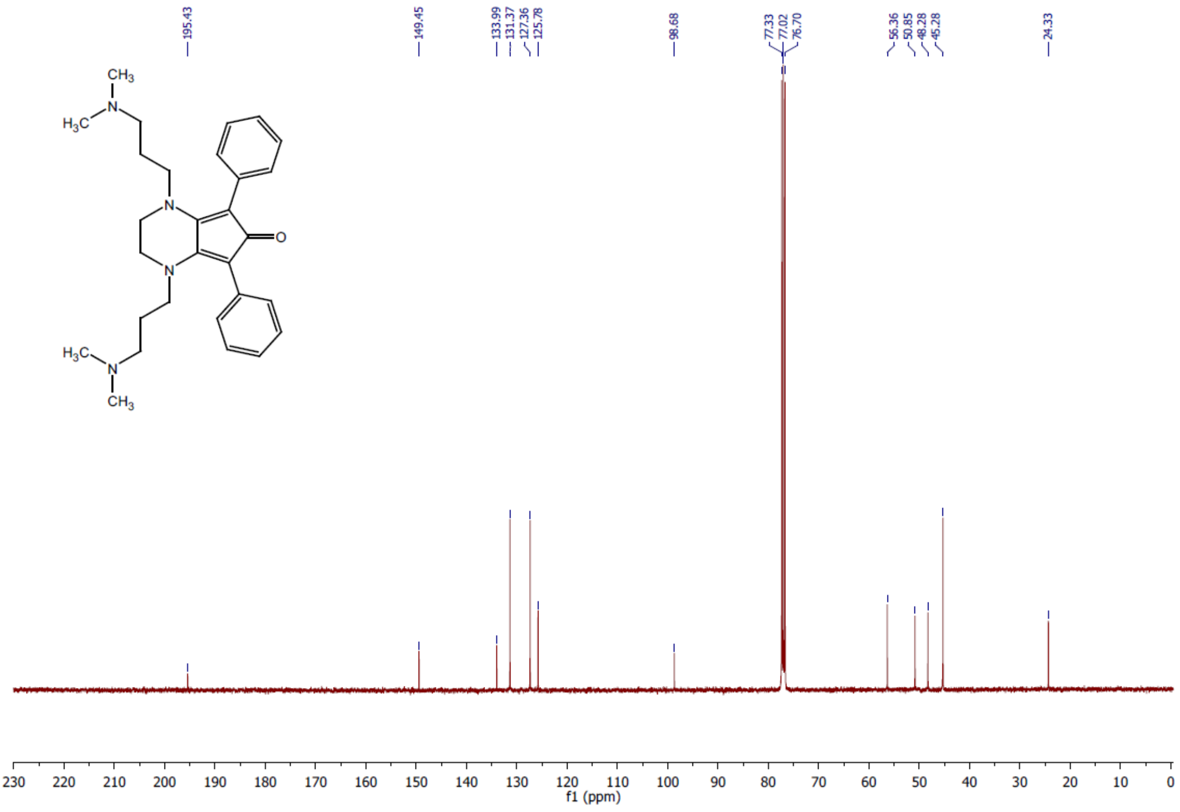

Figure S74: ^{13}C-NMR spectrum of 1,4-bis(3-(dimethylamino)propyl)-5,7-diphenyl-1,2,3,4-tetrahydro-6*H*-cyclopenta[*b*]pyrazin-6-one (**1**).

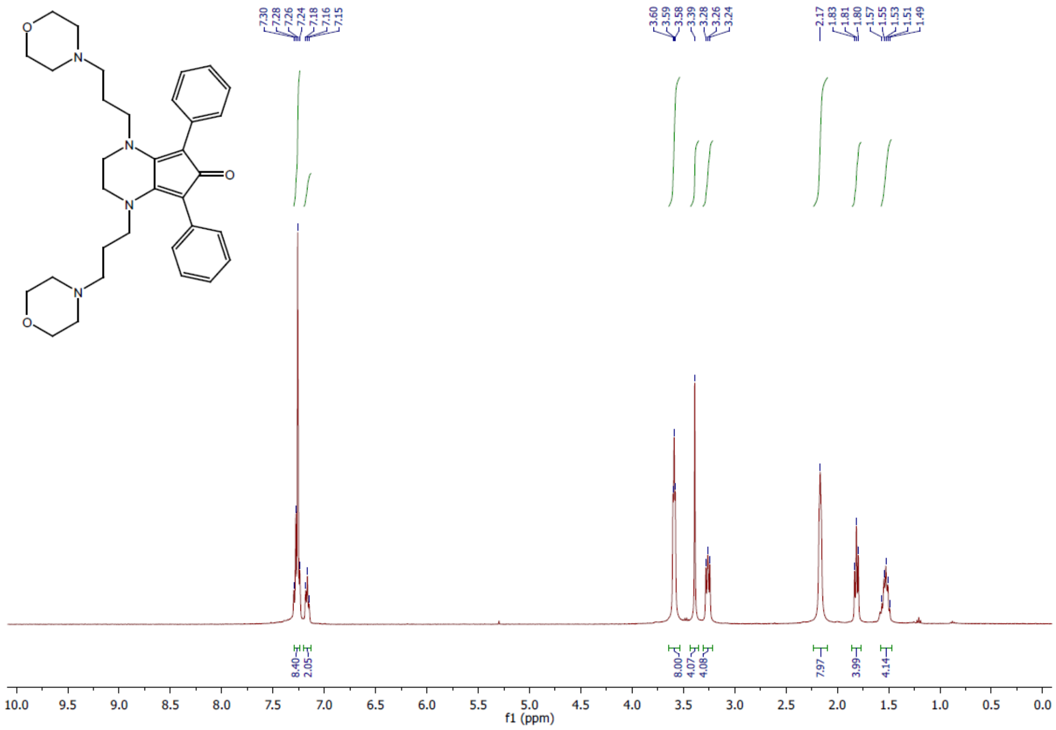

Figure S75: ^{1}H-NMR spectrum of 1,4-bis(3-morpholinopropyl)-5,7-diphenyl-1,2,3,4-tetrahydro-6*H*-cyclopenta[*b*]pyrazin-6-one (**2**).

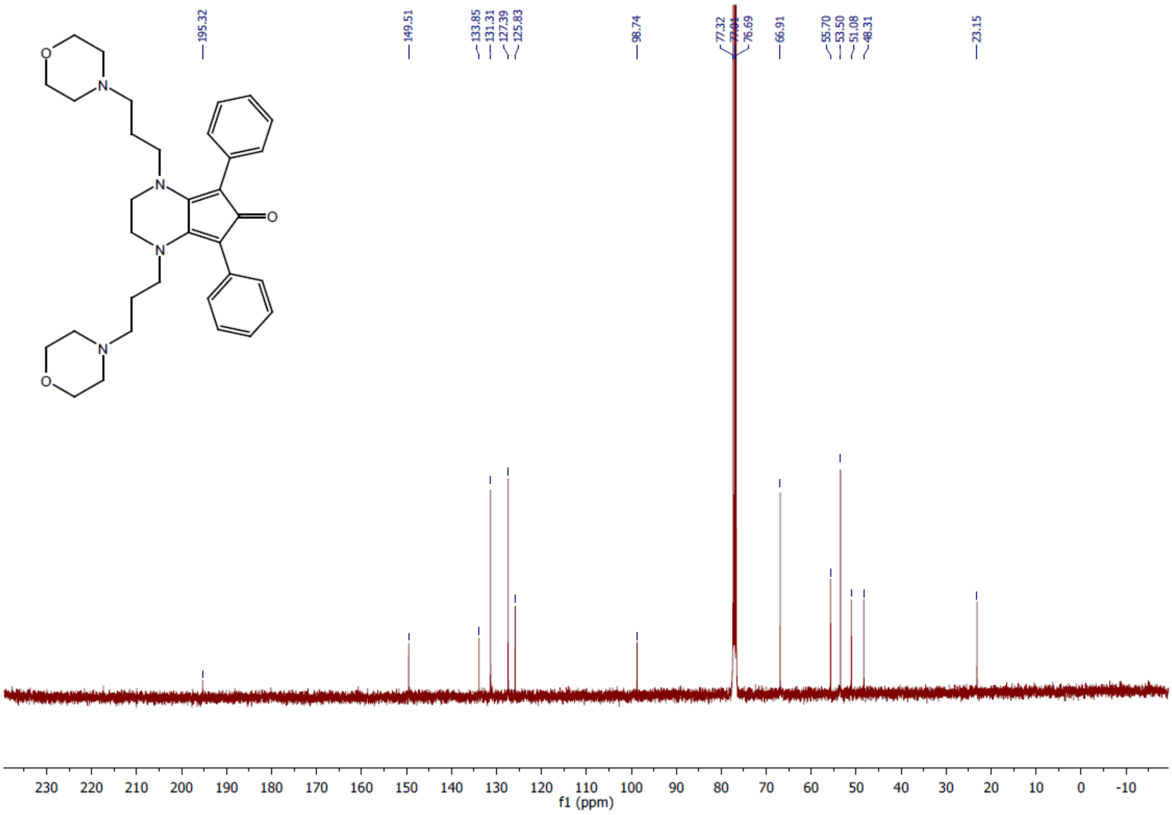

Figure S76: ^{13}C-NMR spectrum of 1,4-bis(3-morpholinopropyl)-5,7-diphenyl-1,2,3,4-tetrahydro-6*H*-cyclopenta[*b*]pyrazin-6-one (**2**).

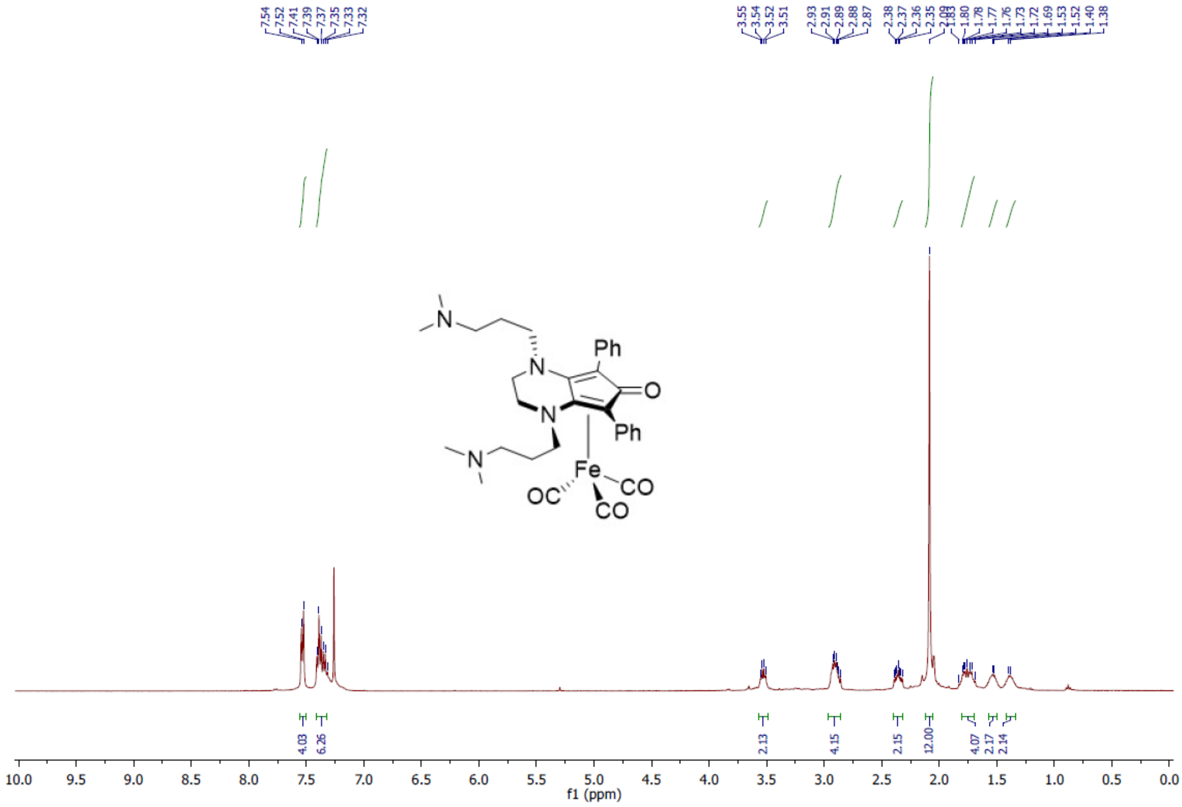

Figure S77: ^{1}H-NMR spectrum of iron complex **Fe6**.

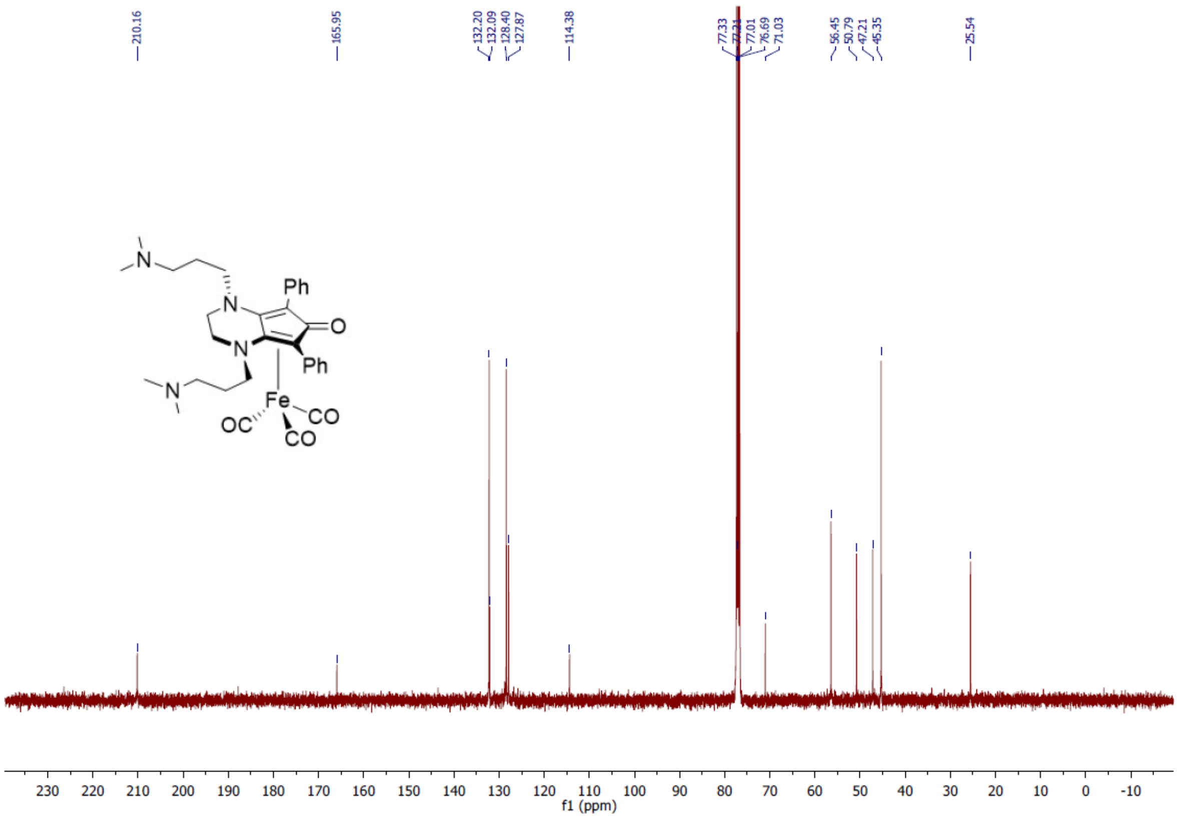

Figure S78: ^{13}C-NMR spectrum of iron complex **Fe6**.

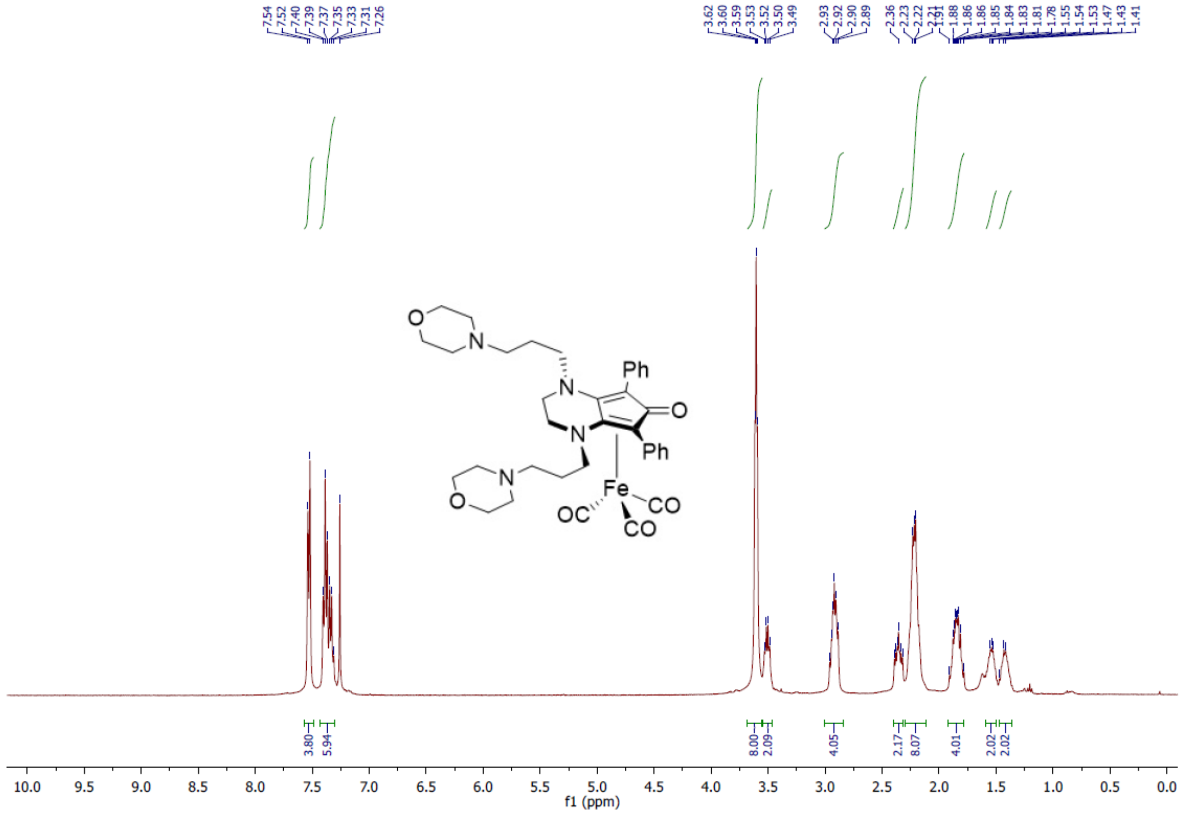

Figure S79: ^{1}H-NMR spectrum of iron complex **Fe7**.

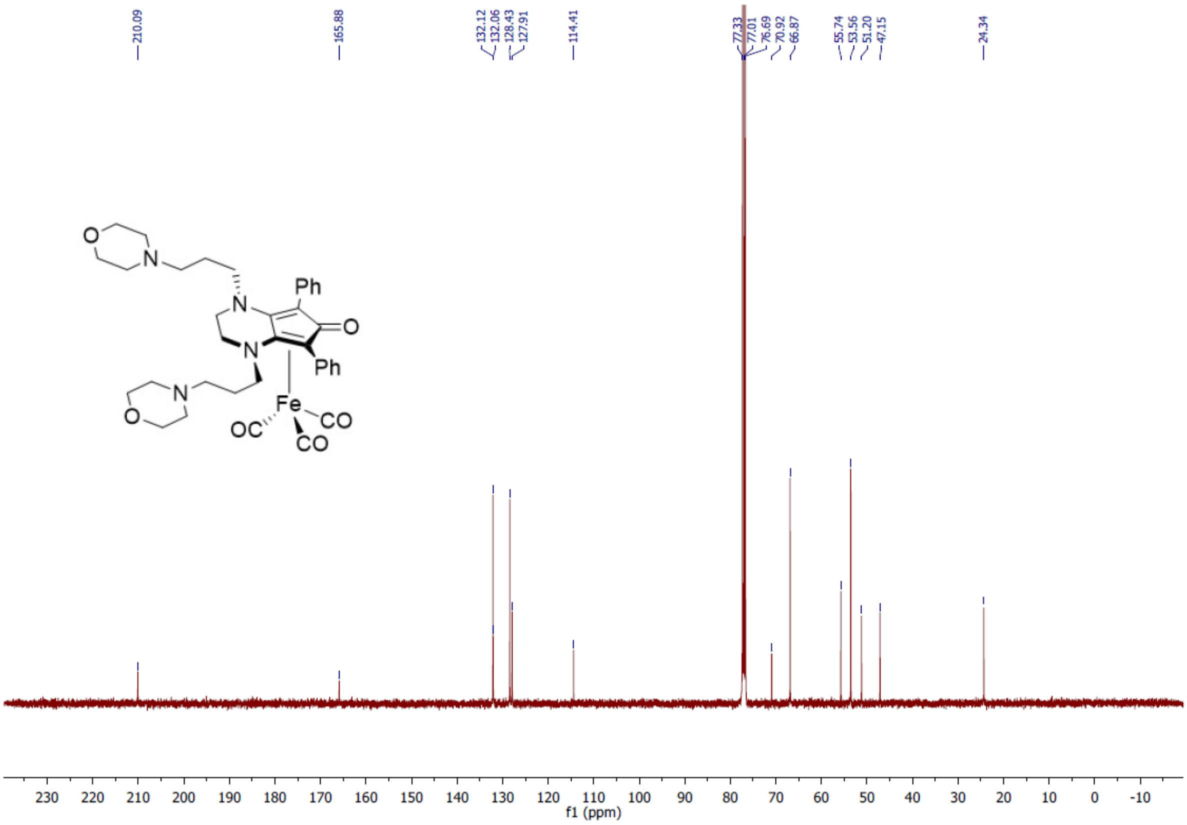

Figure S80: ^{13}C-NMR spectrum of iron complex **Fe7**.

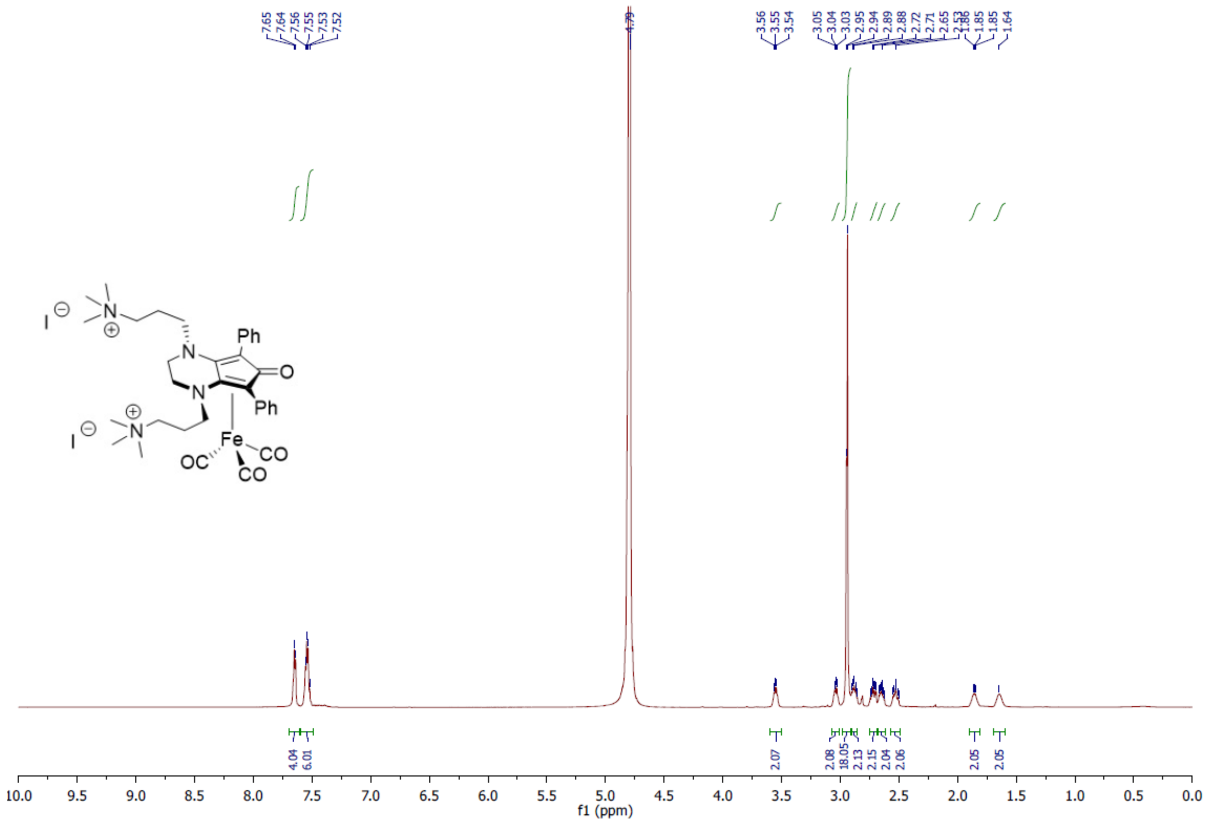

Figure S81: ^{1}H-NMR spectrum of water-soluble iron complex **Fe4.**

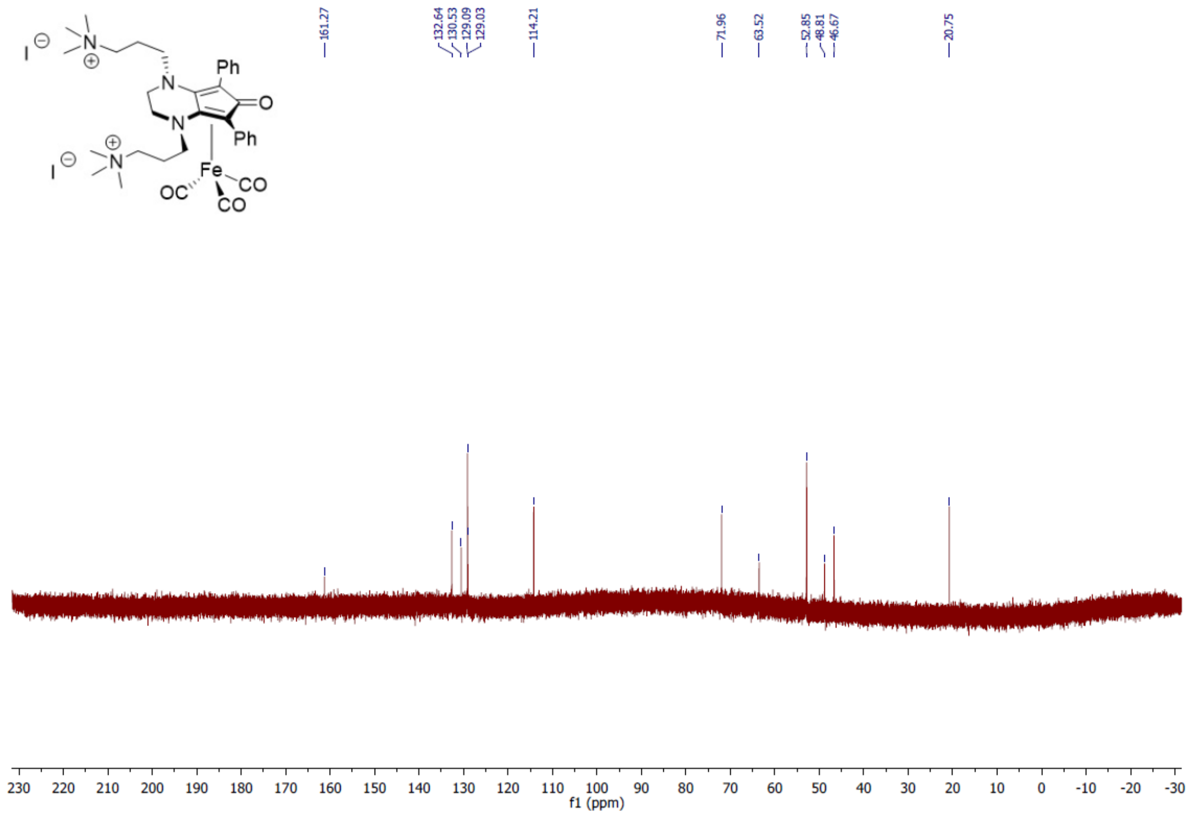

Figure S82: ^{13}C-NMR spectrum of water-soluble iron complex **Fe4.**

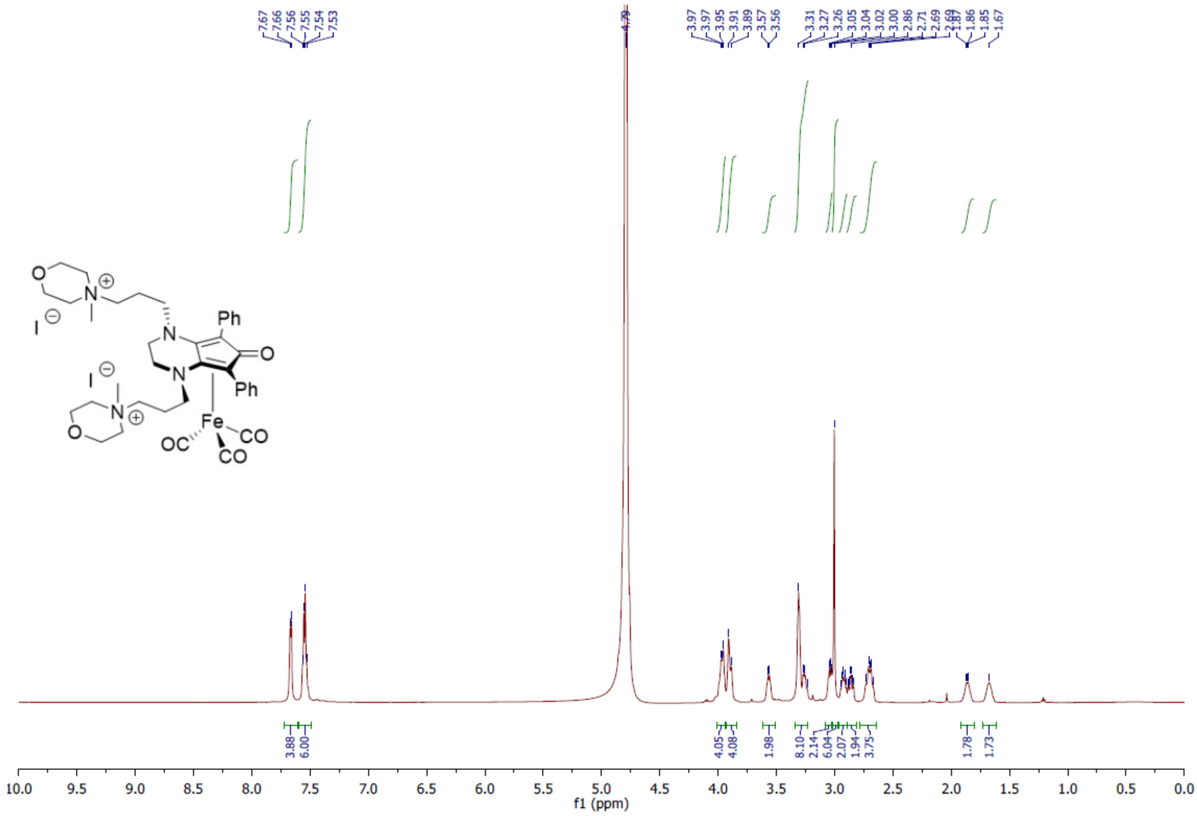

Figure S83: ^{1}H-NMR spectrum of water-soluble iron complex **Fe5.**

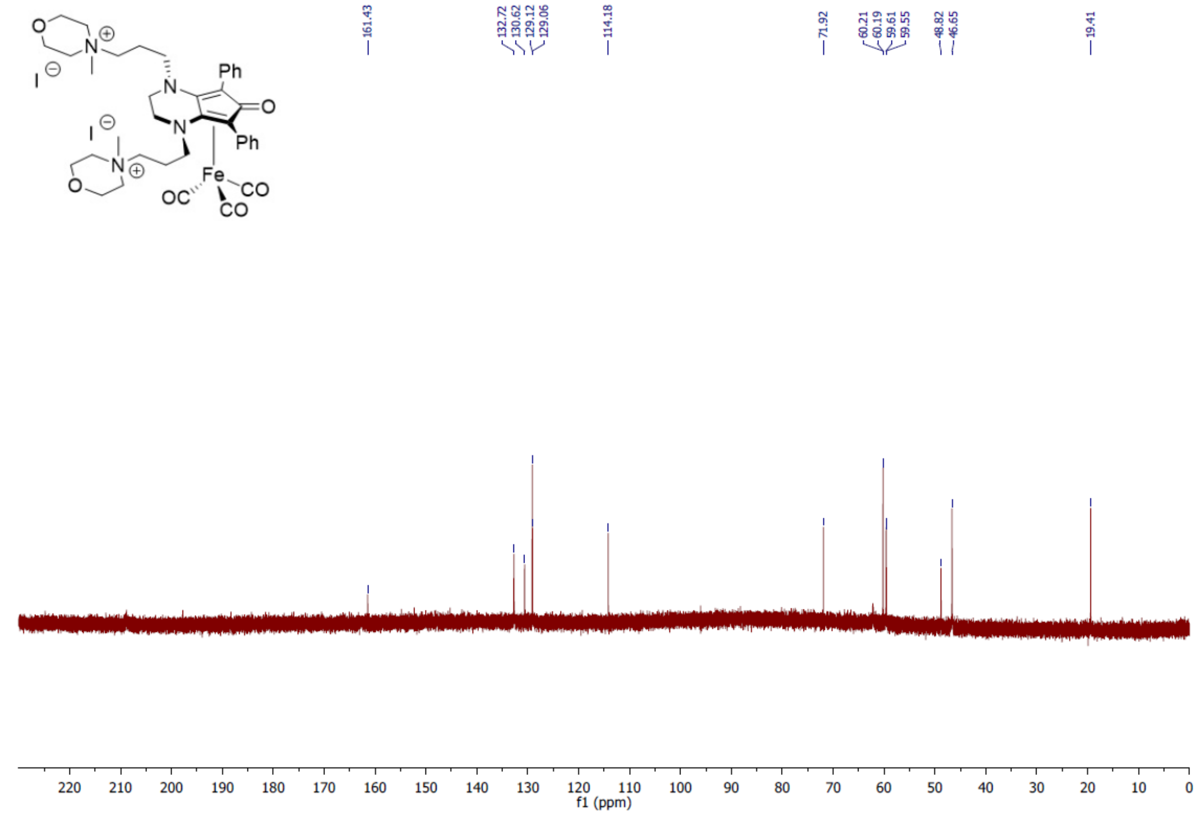

Figure S84: ^{13}C-NMR spectrum of water-soluble iron complex **Fe5.**
